# Global, regional, and national burden of colorectal cancer and its risk factors, 1990–2019: a systematic analysis for the Global Burden of Disease Study 2019

**DOI:** 10.1016/S2468-1253(22)00044-9

**Published:** 2022-04-07

**Authors:** GBD 2019 Colorectal Cancer Collaborators, Rajesh Sharma, Mohsen Abbasi-Kangevari, Rami Abd-Rabu, Hassan Abidi, Eman Abu-Gharbieh, Juan Manuel Acuna, Sangeet Adhikari, Shailesh M Advani, Muhammad Sohail Afzal, Mohamad Aghaie Meybodi, Bright Opoku Ahinkorah, Sajjad Ahmad, Ali Ahmadi, Sepideh Ahmadi, Haroon Ahmed, Luai A Ahmed, Muktar Beshir Ahmed, Hanadi Al Hamad, Fares Alahdab, Fahad Mashhour Alanezi, Turki M Alanzi, Fadwa Alhalaiqa Naji Alhalaiqa, Yousef Alimohamadi, Vahid Alipour, Syed Mohamed Aljunid, Motasem Alkhayyat, Sami Almustanyir, Rajaa M Al-Raddadi, Saba Alvand, Nelson Alvis-Guzman, Saeed Amini, Robert Ancuceanu, Amir Anoushiravani, Ali Arash Anoushirvani, Alireza Ansari-Moghaddam, Jalal Arabloo, Armin Aryannejad, Mohammad Asghari Jafarabadi, Seyyed Shamsadin Athari, Floriane Ausloos, Marcel Ausloos, Atalel Fentahun Awedew, Mamaru Ayenew Awoke, Tegegn Mulatu Ayana, Sina Azadnajafabad, Hiva Azami, Mohammadreza Azangou-Khyavy, Amirhossein Azari Jafari, Ashish D Badiye, Sara Bagherieh, Saeed Bahadory, Atif Amin Baig, Jennifer L Baker, Maciej Banach, Amadou Barrow, Alemshet Yirga Berhie, Sima Besharat, Devidas S Bhagat, Akshaya Srikanth Bhagavathula, Neeraj Bhala, Krittika Bhattacharyya, Vijayalakshmi S Bhojaraja, Sadia Bibi, Ali Bijani, Antonio Biondi, Tone Bjørge, Belay Boda Abule Bodicha, Dejana Braithwaite, Hermann Brenner, Daniela Calina, Chao Cao, Yin Cao, Giulia Carreras, Felix Carvalho, Ester Cerin, Raja Chandra Chakinala, William C S Cho, Dinh-Toi Chu, Joao Conde, Vera Marisa Costa, Natália Cruz-Martins, Omid Dadras, Xiaochen Dai, Lalit Dandona, Rakhi Dandona, Anna Danielewicz, Feleke Mekonnen Demeke, Getu Debalkie Demissie, Rupak Desai, Deepak Dhamnetiya, Mostafa Dianatinasab, Daniel Diaz, Mojtaba Didehdar, Saeid Doaei, Linh Phuong Doan, Milad Dodangeh, Fatemeh Eghbalian, Debela Debela Ejeta, Michael Ekholuenetale, Temitope Cyrus Ekundayo, Iman El Sayed, Muhammed Elhadi, Daniel Berhanie Enyew, Tahir Eyayu, Rana Ezzeddini, Ildar Ravisovich Fakhradiyev, Umar Farooque, Hossein Farrokhpour, Farshad Farzadfar, Ali Fatehizadeh, Hamed Fattahi, Nima Fattahi, Masood Fereidoonnezhad, Eduarda Fernandes, Getahun Fetensa, Irina Filip, Florian Fischer, Masoud Foroutan, Peter Andras Gaal, Mohamed M Gad, Silvano Gallus, Tushar Garg, Tamiru Getachew, Seyyed-Hadi Ghamari, Ahmad Ghashghaee, Nermin Ghith, Maryam Gholamalizadeh, Jamshid Gholizadeh Navashenaq, Abraham Tamirat Gizaw, James C Glasbey, Mahaveer Golechha, Pouya Goleij, Kebebe Bekele Gonfa, Giuseppe Gorini, Avirup Guha, Sapna Gupta, Veer Bala Gupta, Vivek Kumar Gupta, Rasool Haddadi, Nima Hafezi-Nejad, Arvin Haj-Mirzaian, Rabih Halwani, Shafiul Haque, Sanam Hariri, Ahmed I Hasaballah, Soheil Hassanipour, Simon I Hay, Claudiu Herteliu, Ramesh Holla, Mohammad-Salar Hosseini, Mehdi Hosseinzadeh, Mihaela Hostiuc, Mowafa Househ, Junjie Huang, Ayesha Humayun, Ivo Iavicoli, Olayinka Stephen Ilesanmi, Irena M Ilic, Milena D Ilic, Farhad Islami, Masao Iwagami, Mohammad Ali Jahani, Mihajlo Jakovljevic, Tahereh Javaheri, Ranil Jayawardena, Rime Jebai, Ravi Prakash Jha, Tamas Joo, Nitin Joseph, Farahnaz Joukar, Jacek Jerzy Jozwiak, Ali Kabir, Rohollah Kalhor, Ashwin Kamath, Neeti Kapoor, Ibraheem M Karaye, Amirali Karimi, Joonas H Kauppila, Asma Kazemi, Mohammad Keykhaei, Yousef Saleh Khader, Himanshu Khajuria, Rovshan Khalilov, Javad Khanali, Maryam Khayamzadeh, Mahmoud Khodadost, Hanna Kim, Min Seo Kim, Adnan Kisa, Sezer Kisa, Ali-Asghar Kolahi, Hamid Reza Koohestani, Jacek A Kopec, Rajasekaran Koteeswaran, Ai Koyanagi, Yuvaraj Krishnamoorthy, G Anil Kumar, Manoj Kumar, Vivek Kumar, Carlo La Vecchia, Faris Hasan Lami, Iván Landires, Caterina Ledda, Sang-woong Lee, Wei-Chen Lee, Yeong Yeh Lee, Elvynna Leong, Bingyu Li, Stephen S Lim, Stany W Lobo, Joana A Loureiro, Raimundas Lunevicius, Farzan Madadizadeh, Ata Mahmoodpoor, Azeem Majeed, Mohammad-Reza Malekpour, Reza Malekzadeh, Ahmad Azam Malik, Fariborz Mansour-Ghanaei, Lorenzo Giovanni Mantovani, Miquel Martorell, Sahar Masoudi, Prashant Mathur, Jitendra Kumar Meena, Entezar Mehrabi Nasab, Walter Mendoza, Alexios-Fotios A Mentis, Tomislav Mestrovic, Junmei Miao Jonasson, Bartosz Miazgowski, Tomasz Miazgowski, Gelana Fekadu Worku Mijena, Seyyedmohammadsadeq Mirmoeeni, Mohammad Mirza-Aghazadeh-Attari, Hamed Mirzaei, Sanjeev Misra, Karzan Abdulmuhsin Mohammad, Esmaeil Mohammadi, Saeed Mohammadi, Seyyede Momeneh Mohammadi, Abdollah Mohammadian-Hafshejani, Shafiu Mohammed, Teroj Abdulrahman Mohammed, Nagabhishek Moka, Ali H Mokdad, Zeinab Mokhtari, Mariam Molokhia, Sara Momtazmanesh, Lorenzo Monasta, Ghobad Moradi, Rahmatollah Moradzadeh, Paula Moraga, Joana Morgado-da-Costa, Sumaira Mubarik, Francesk Mulita, Mohsen Naghavi, Mukhammad David Naimzada, Hae Sung Nam, Zuhair S Natto, Biswa Prakash Nayak, Javad Nazari, Ehsan Nazemalhosseini-Mojarad, Ionut Negoi, Cuong Tat Nguyen, Son Hoang Nguyen, Nurulamin M Noor, Maryam Noori, Seyyed Mohammad Ali Noori, Virginia Nuñez-Samudio, Chimezie Igwegbe Nzoputam, Bogdan Oancea, Oluwakemi Ololade Odukoya, Ayodipupo Sikiru Oguntade, Hassan Okati-Aliabad, Andrew T Olagunju, Tinuke O Olagunju, Sokking Ong, Samuel M Ostroff, Alicia Padron-Monedero, Reza Pakzad, Adrian Pana, Anamika Pandey, Fatemeh Pashazadeh Kan, Urvish K Patel, Uttam Paudel, Renato B Pereira, Navaraj Perumalsamy, Richard G Pestell, Zahra Zahid Piracha, Richard Charles G Pollok, Akram Pourshams, Naeimeh Pourtaheri, Akila Prashant, Mohammad Rabiee, Navid Rabiee, Amir Radfar, Sima Rafiei, Mosiur Rahman, Amir Masoud Rahmani, Vahid Rahmanian, Nazanin Rajai, Aashish Rajesh, Vajiheh Ramezani-Doroh, Kiana Ramezanzadeh, Kamal Ranabhat, Sina Rashedi, Amirfarzan Rashidi, Mahsa Rashidi, Mohammad-Mahdi Rashidi, Mandana Rastegar, David Laith Rawaf, Salman Rawaf, Reza Rawassizadeh, Mohammad Sadegh Razeghinia, Andre M N Renzaho, Negar Rezaei, Nima Rezaei, Saeid Rezaei, Mohsen Rezaeian, Sahba Rezazadeh-Khadem, Gholamreza Roshandel, Maha Mohamed Saber-Ayad, Bahar Saberzadeh-Ardestani, Basema Saddik, Hossein Sadeghi, Umar Saeed, Maryam Sahebazzamani, Amirhossein Sahebkar, Amir Salek Farrokhi, Amir Salimi, Hamideh Salimzadeh, Pouria Samadi, Mehrnoosh Samaei, Abdallah M Samy, Juan Sanabria, Milena M Santric-Milicevic, Muhammad Arif Nadeem Saqib, Arash Sarveazad, Brijesh Sathian, Maheswar Satpathy, Ione Jayce Ceola Schneider, Mario Šekerija, Sadaf G Sepanlou, Allen Seylani, Feng Sha, Sayed Mohammad Shafiee, Zahra Shaghaghi, Saeed Shahabi, Elaheh Shaker, Maedeh Sharifian, Javad Sharifi-Rad, Sara Sheikhbahaei, Jeevan K Shetty, Reza Shirkoohi, Parnian Shobeiri, Sudeep K Siddappa Malleshappa, Diego Augusto Santos Silva, Guilherme Silva Julian, Achintya Dinesh Singh, Jasvinder A Singh, Md Shahjahan Siraj, Gholam Reza Sivandzadeh, Valentin Yurievich Skryabin, Anna Aleksandrovna Skryabina, Bogdan Socea, Marco Solmi, Mohammad Sadegh Soltani-Zangbar, Suhang Song, Viktória Szerencsés, Miklós Szócska, Rafael Tabarés-Seisdedos, Elnaz Tabibian, Majid Taheri, Yasaman TaheriAbkenar, Amir Taherkhani, Iman M Talaat, Ker-Kan Tan, Abdelghani Tbakhi, Bekele Tesfaye, Amir Tiyuri, Daniel Nigusse Tollosa, Mathilde Touvier, Bach Xuan Tran, Biruk Shalmeno Tusa, Irfan Ullah, Saif Ullah, Marco Vacante, Sahel Valadan Tahbaz, Massimiliano Veroux, Bay Vo, Theo Vos, Cong Wang, Ronny Westerman, Melat Woldemariam, Seyed Hossein Yahyazadeh Jabbari, Lin Yang, Fereshteh Yazdanpanah, Chuanhua Yu, Deniz Yuce, Ismaeel Yunusa, Vesna Zadnik, Mazyar Zahir, Iman Zare, Zhi-Jiang Zhang, Mohammad Zoladl

## Abstract

**Background:**

Colorectal cancer is the third leading cause of cancer deaths worldwide. Given the recent increasing trends in colorectal cancer incidence globally, up-to-date information on the colorectal cancer burden could guide screening, early detection, and treatment strategies, and help effectively allocate resources. We examined the temporal patterns of the global, regional, and national burden of colorectal cancer and its risk factors in 204 countries and territories across the past three decades.

**Methods:**

Estimates of incidence, mortality, and disability-adjusted life years (DALYs) for colorectal cancer were generated as a part of the Global Burden of Diseases, Injuries and Risk Factors Study (GBD) 2019 by age, sex, and geographical location for the period 1990–2019. Mortality estimates were produced using the cause of death ensemble model. We also calculated DALYs attributable to risk factors that had evidence of causation with colorectal cancer.

**Findings:**

Globally, between 1990 and 2019, colorectal cancer incident cases more than doubled, from 842 098 (95% uncertainty interval [UI] 810 408–868 574) to 2·17 million (2·00–2·34), and deaths increased from 518 126 (493 682–537 877) to 1·09 million (1·02–1·15). The global age-standardised incidence rate increased from 22·2 (95% UI 21·3–23·0) per 100 000 to 26·7 (24·6–28·9) per 100 000, whereas the age-standardised mortality rate decreased from 14·3 (13·5–14·9) per 100 000 to 13·7 (12·6–14·5) per 100 000 and the age-standardised DALY rate decreased from 308·5 (294·7–320·7) per 100 000 to 295·5 (275·2–313·0) per 100 000 from 1990 through 2019. Taiwan (province of China; 62·0 [48·9–80·0] per 100 000), Monaco (60·7 [48·5–73·6] per 100 000), and Andorra (56·6 [42·8–71·9] per 100 000) had the highest age-standardised incidence rates, while Greenland (31·4 [26·0–37·1] per 100 000), Brunei (30·3 [26·6–34·1] per 100 000), and Hungary (28·6 [23·6–34·0] per 100 000) had the highest age-standardised mortality rates. From 1990 through 2019, a substantial rise in incidence rates was observed in younger adults (age <50 years), particularly in high Socio-demographic Index (SDI) countries. Globally, a diet low in milk (15·6%), smoking (13·3%), a diet low in calcium (12·9%), and alcohol use (9·9%) were the main contributors to colorectal cancer DALYs in 2019.

**Interpretation:**

The increase in incidence rates in people younger than 50 years requires vigilance from researchers, clinicians, and policy makers and a possible reconsideration of screening guidelines. The fast-rising burden in low SDI and middle SDI countries in Asia and Africa calls for colorectal cancer prevention approaches, greater awareness, and cost-effective screening and therapeutic options in these regions.

**Funding:**

Bill & Melinda Gates Foundation.

## Introduction

In 2019, colorectal cancer was the third leading cause of cancer deaths and the second leading cause of disability-adjusted life years (DALYs) for cancer worldwide.^1^ Around 70–75% of colorectal cancer cases occur sporadically and are associated with modifiable risk factors, whereas 25–30% of cases are linked to non-modifiable risk factors such as genetic factors, a personal history of polyps or adenoma, or a family history of colorectal cancer or hereditary risk (eg, Lynch syndrome or familial adenomatous polyposis).^2^, ^3^, ^4^, ^5^ Because of the increased prevalence of modifiable risk factors such as smoking, alcohol consumption, unhealthy diets, sedentary behaviour, physical inactivity, obesity, increasing life expectancy, increasing awareness and affordability of colorectal cancer screening, and increasing screening capacity, incident cases of colorectal cancer are growing rapidly in low-income and middle-income countries (LMICs).^6^, ^7^

The UN Sustainable Development Goal (SDG) target 3.4 focuses on reduction of premature mortality from non-communicable diseases (including cancers) by a third by 2030.^8^ Colorectal cancer can be prevented by ameliorating modifiable risk factors, and deaths can be prevented through early detection of polyps with proven screening interventions;^9^, ^10^, ^11^ therefore, addressing the global colorectal cancer burden must serve as one of the considerations towards progress on SDG 3.4 relating to non-communicable diseases. In this endeavour, recent changes in the colorectal cancer burden should be tracked at the global, regional, and national levels to identify those countries making progress and those countries and regions where more work is needed.

**Research in context**

**Evidence before this study**

Colorectal cancer is one of the leading causes of cancer deaths worldwide. Previously, the Global Burden of Diseases, Injuries, and Risk Factors Study (GBD) 2017 provided estimates for colorectal cancer incidence, deaths, and disability-adjusted life years (DALYs) for the period 1990–2017. Apart from GBD 2017, the International Agency for Research on Cancer (IARC) provided estimates for colorectal cancer for 2020 under the GLOBOCAN project. The present study was done as a part of GBD 2019, which produced estimates for 302 causes of death, 369 diseases and injuries, and 87 risk factors for 204 countries and territories for 1990–2019.

**Added value of this study**

In this study, we provide age-sex-location-specific estimates of colorectal cancer incidence, deaths, and DALYs for 204 countries and territories between 1990 and 2019. GBD 2019 produced estimates with technical collaboration from WHO, which has led to the inclusion of nine more WHO member countries. 28 714 site-years of data were used to estimate colorectal cancer incidence, deaths, and DALYs in GBD 2019, 16% more than in GBD 2017. In comparison with GLOBOCAN 2020, which provided colorectal cancer estimates for 2020, we provide estimates for full time series through 1990 to 2019 for all 204 countries and territories included in GBD 2019. Apart from estimates of incident cases, deaths, and age-standardised rates, as was done in GLOBOCAN 2020, we also estimated the burden of deaths and disability quantified with DALYs. The colorectal cancer burden was also examined in the light of country-level socioeconomic development measured by Socio-demographic Index (SDI). The contribution of the main risk factors to colorectal cancer DALYs was also examined by sex in 21 world regions.

**Implications of all the available evidence**

Incident cases of colorectal cancer doubled or more than doubled in 16 of 21 world regions, and the number of deaths doubled or more than doubled in 15 of 21 world regions in the past three decades. The age-standardised incidence and death rates (per 100 000 person-years) either remained the same or decreased in high SDI quintiles and increased in low SDI and middle SDI quintiles. Large increases in colorectal cancer incidence rates were observed in middle SDI countries, as well as in people aged 20–49 years in high SDI countries. Further research is required to understand the causes of the colorectal cancer burden in younger adults (aged <50 years) and the main risk factors, including obesity, physical inactivity, alcohol consumption, smoking, and an altered gut microbiome, that might have led to the rise in the colorectal cancer burden. The increasing incidence of colorectal cancer in people younger than 50 years in high SDI countries also necessitates reconsideration of screening recommendations to include younger age groups (ie, those aged 40–49 years). The public health interventions for colorectal cancer awareness, screening, and prevention through containment of modifiable risk factors such as alcohol, smoking, an unhealthy diet (high in processed meat and fat, and low in fruits and vegetables), and obesity are key to stemming the tide of colorectal cancer worldwide.

This study aimed to investigate the global, regional, and national burden of colorectal cancer in 204 countries and territories from 1990 to 2019. We examined the age-sex-location-specific burden of colorectal cancer using estimates from the Global Burden of Diseases, Injuries, and Risk Factors Study (GBD) 2019.^1^, ^12^, ^13^, ^14^ The colorectal cancer burden was examined in light of the development status of countries measured by the Socio-demographic Index (SDI). We also aimed to quantify health loss due to colorectal cancer using DALYs, which encompasses the burden of a disease due to both deaths and disabilities caused by it. Last, we also examined the risk-attributable burden of the main risk factors for colorectal cancer. Apart from GBD, the International Agency for Research on Cancer (IARC) produced cancer estimates for 2020; however, GBD estimates facilitate examination of temporal patterns at global, regional, and national levels. An assessment of recent trends at the global, regional, and national levels, and the associated risk factors for countries at different levels of development, can help track progress, map resource requirements, and help in policy making and implementation towards prevention and tackling the growing burden of colorectal cancer.

This manuscript was produced as part of the GBD Collaborator Network and in accordance with the GBD Protocol.

## Methods

### Overview

The GBD 2019 estimates were generated for 286 causes of death, 369 causes of non-fatal burden, and 87 risk factors.^1^, ^12^, ^13^, ^14^ In comparison with the 195 countries and territories included in GBD 2017, nine more countries (Cook Islands, Monaco, San Marino, Nauru, Niue, Palau, Saint Kitts and Nevis, Tokelau, and Tuvalu) were included in GBD 2019, providing full time-series estimates from 1990 to 2019 for 204 countries and territories, which were grouped under seven super-regions and 21 regions. The colorectal cancer estimates mapped to the International Classification of Diseases (ICD) codes are available in the appendix (p 14).^15^ The GBD estimation framework and calculation of all the metrics are detailed in GBD 2019 capstone publications and the supplementary appendices published with these reports.^1^, ^12^, ^13^, ^14^

### Data sources

For GBD 2019, input data from various sources such as vital registration, verbal autopsy, and cancer registries were used to generate the colorectal cancer estimates. 28 714 site-years of data (22 849 site-years for vital registration, 516 site-years for verbal autopsy, and 5349 site-years for cancer registries) were used to estimate the colorectal cancer burden in GBD 2019, 3962 site-years (16%) more than GBD 2017. The vital registration system records data of vital events in a person's life (birth, death, and cause of death). Verbal autopsy is a data collection method generally used in populations without a complete vital registration system; in this method, a trained interviewer uses a questionnaire to collect information about signs, symptoms, and demographic characteristics of a recently deceased person from someone familiar with the deceased. Cancer registries are a data collection system that record and manage the data relating to a person with cancer. The information on various input sources of data used in GBD 2019 can be obtained from the GBD 2019 Data Input Sources Tool.

### Mortality estimation

GBD estimation begins with mortality estimation in multiple steps. The mortality data from cancer registries might be sparse, although incidence data can be available; therefore, to maximise data availability, mortality-to-incidence ratios (MIRs) were generated from the cancer registries that contained both incidence and mortality data. In the first step, incidence and mortality data from cancer registries were processed before they were matched by cancer, age, sex, year, and location to generate crude MIRs. Final MIRs were estimated by use of spatio-temporal Gaussian Process Regression (ST-GPR) using the Healthcare Access and Quality (HAQ) index, age, and sex^16^ as covariates. The MIR estimates from the ST-GPR model were multiplied with incidence data to generate crude mortality estimates. The final mortality estimates were produced with the Cause of Death Ensemble Model (CODEm) using crude mortality estimates from the last step and those from vital registration and verbal autopsy as inputs along with other variables taken as covariates.^1^, ^15^ Only those variables were chosen that have been found to have a plausible relation with death due to colorectal cancer. The list of covariates at different levels used in CODEm for colorectal cancer is presented in the appendix (pp 15–16).^1^, ^15^ The final mortality estimates from CODEm were then divided by the MIRs from ST-GPR to generate age-sex-location-specific estimates for incident cases.

### Non-fatal estimation

The mortality estimates generated from CODEm were combined with reference life tables to generate estimates for years of life lost (YLLs).^12^ The 10-year prevalence was divided into four sequelae of a fixed duration based on expected person-time spent in each of the sequela: diagnosis and primary therapy (4·0 months), metastatic phase (9·7 months), and terminal phase (1·0 month), with the remaining duration assigned to the controlled phase.^15^ Sequela-specific years lived with disability (YLDs) were calculated by multiplying disability weights with sequelae-specific prevalence. Total YLDs were calculated by summing the sequela-specific YLDs. The sum of YLLs and YLDs produced the DALYs estimates, with one DALY being equivalent to 1 year of healthy life lost.^17^

The age-specific rates of incidence, mortality, and DALYs were expressed per 100 000 person-years and calculated with GBD population estimates,^12^ and the age-standardised rates were calculated as weighted averages of age-specific rates per 100 000 people, in which weights are the proportion of people in corresponding age groups as per the GBD world population age standard.^12^ All GBD estimates in this Article are provided with 95% uncertainty intervals (UIs). For each computational step, 1000 draws are generated; 95% UIs are calculated by taking values at the 2·5th and 97·5th percentile from the 1000 draws, and are provided alongside the mean estimates.^12^

### Socio-demographic Index

The colorectal cancer burden was evaluated against country-level development measured with the SDI,^12^, ^15^ which is a composite indicator of three indicators: lag-distributed income per capita, average educational attainment for people aged 15 years and older, and the total fertility rate (in people aged <25 years). Each of these indicators was first rescaled on a scale of 0 (lowest) to 1 (highest) based on country-specific values. The geometric mean of these three indices provided the final value of the country-level SDI. Based on SDI values, the 204 countries and territories were categorised into five groups: low SDI (<0·45), low-middle SDI (≥0·45 and <0·61), middle SDI (≥0·61 and <0·69), high-middle SDI (≥0·69 and <0·80), and high SDI (≥0·80).

### Risk factors

Estimation of GBD risk factors is based on a comparative risk assessment framework and involves six steps. The first is identification of risk-outcome pairs: only those risk-outcomes that have convincing or plausible evidence, as per World Cancer Research Fund criteria,^18^ are included in GBD risk factor estimation. The second is estimation of relative risk (RR) as a function of exposure for each risk-outcome pair. The third is distribution of exposure for each risk factor by age, sex, location, and year. The fourth is determining the theoretical minimum risk exposure level (TMREL). The fifth is estimation of the population attributable fraction (PAF) and attributable burden. The RR for each risk-outcome pair, exposure levels, and TMREL are used to model the PAF.^13^ The PAF of a particular risk factor is multiplied by colorectal cancer DALYs to generate the DALYs attributable to that risk factor. The sixth is estimating the PAF and attributable burden for the combination of risk factors.

The details of each of these steps and the underlying methodology are provided elsewhere.^13^ In this GBD iteration, 87 risk factors were included, of which ten (alcohol use, diet high in processed meat, diet high in red meat, diet low in calcium, diet low in fibre, diet low in milk, high body-mass index [BMI], high fasting plasma glucose, low physical activity, and smoking) have a non-zero contribution to colorectal cancer deaths and DALYs. We assessed the percentage contribution of these ten risk factors to colorectal cancer DALYs in 2019. The total number of input data sources used for the exposure of different risk factors in GBD 2019 are detailed elsewhere,^13^ of which we present the number of input data sources for the ten risk factors related to colorectal cancer (appendix p 17).

The percentage changes between 1990 and 2019 were interpreted as statistically significant if the 95% UI did not include zero. All data analysis and data visualisation in this study were done with statistical software R (version 4.1.1), Stata (version 13.1), and Python (version 3.8.8).

### Role of the funding source

The funders of the study had no role in study design, data collection, data analysis, data interpretation, or writing of the report.

## Results

### Overview of the global burden

**Figure 1:**
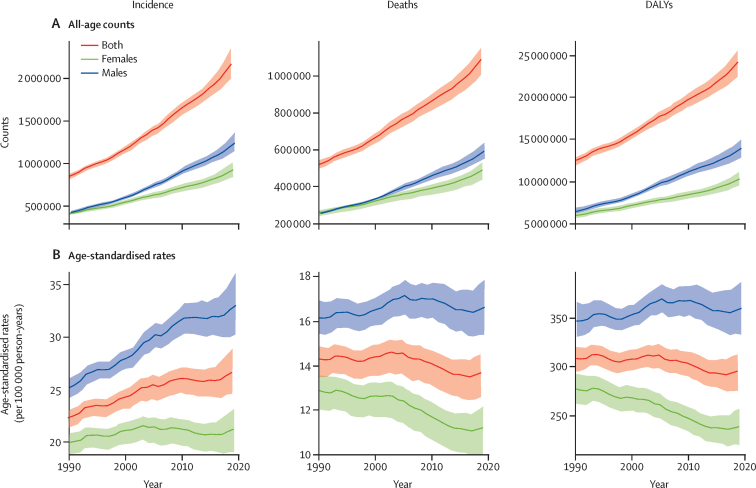
Global temporal patterns of colorectal cancer burden, 1990–2019 (A) All-age counts. (B) Age-standardised rates. Data source: Global Burden of Diseases, Injuries, and Risk Factors Study 2019. DALYs=disability-adjusted life-years.

Globally, for both sexes combined, incident cases of colorectal cancer more than doubled, from 842 098 (95% UI 810 408–868 574) in 1990 to 2·17 million (2·00–2·34) in 2019 (figure 1A). Between 1990 and 2019, deaths due to colorectal cancer increased from 518 126 (493 682–537 877) to 1·09 million (1·02–1·15), and DALYs increased from 12·4 million (11·9–12·9) to 24·3 million (22·6–25·7). By 2019, 95·6% of colorectal cancer DALYs were due to YLLs and 4·4% were due to YLDs (appendix p 5). The global age-standardised incidence rate of colorectal cancer increased from an estimated 22·2 (21·3–23·0) per 100 000 to 26·7 (24·6–28·9) per 100 000 from 1990 through 2019 (figure 1B). By contrast, the global age-standardised mortality rate decreased from 14·3 (13·5–14·9) per 100 000 to 13·7 (12·6–14·5) per 100 000, and the age-standardised DALY rate decreased from 308·5 (294·7–320·7) per 100 000 to 295·5 (275·2–313·0) per 100 000 from 1990 through 2019.

Males experienced greater increases in colorectal cancer incidence, deaths, and DALYs, than females in terms of absolute counts, and the age-standardised rates increased in males and remained similar throughout or decreased in females (figure 1). In 2019, males accounted for 57·2% (1·2 million [95% UI 1·1–1·4]) of colorectal cancer incident cases, and 54·9% (594 176 [550 959–638 031]) of deaths due to colorectal cancer. In 2019, the age-standardised incidence rate was 1·5-times higher in males than in females (33·1 [30·2– 36·1] per 100 000 *vs* 21·2 [19·0–23·2] per 100 000), with a similar disparity between males and females in terms of the age-standardised mortality rate (16·6 [15·4–17·8] per 100 000 in males *vs* 11·2 [10·0–12·2] per 100 000 in females) and the age-standardised DALY rate (360·0 [333·1–387·8] per 100 000 in males *vs* 237·9 [218·7–257·1] per 100 000 in females; figure 1). The number of incident cases increased in all SDI quintiles, whereas the age-standardised incidence rate decreased in the high SDI quintile only (appendix p 6). Similarly, deaths and DALYs increased in all SDI quintiles, but age-standardised mortality rates stagnated or decreased only in high-middle SDI and high SDI quintiles (appendix pp 7–8). Notably, the rise in all-age counts was steeper in all SDI quintiles than in the high SDI quintile such that the share of low, low-middle, middle, and high-middle SDI quintiles in the total colorectal cancer burden increased from 47·3% to 62·1% in terms of incident cases, from 57·1% to 69·8% in terms of deaths, and from 62·4% to 74·6% in terms of DALYs between 1990 and 2019 (appendix pp 6–8).

### Colorectal cancer burden by region

In 2019, east Asia was the worst-affected region, with 637 096 (95% UI 548 895–738 549) new cases, 275 604 (238 238–317 886) deaths, and 6·7 million (5·8–7·7) DALYs due to colorectal cancer (table). Australasia had the highest age-standardised incidence rate (48·3 [39·6–59·1] per 100 000) and central Europe had the highest age-standardised mortality rate (23·6 [20·8–26·4] per 100 000) across 21 regions in 2019. The age-standardised incidence rate was the lowest in central sub-Saharan Africa (7·7 [5·9–10·1] per 100 000) and south Asia (8·3 [7·2–9·4] per 100 000). South Asia also had the lowest age-standardised mortality rate (7·3 [6·2–8·3] per 100 000). The age-standardised DALY rate was the highest in central Europe (512·6 [448·7–577·9] per 100 000) and lowest in south Asia (165·1 [141·7–189·9] per 100 000) in 2019 (table).

**Table:** Region-wise colorectal cancer burden in 2019

	**Incidence (95% UI)**	**Mortality (95% UI)**	**DALYs (95% UI)**	**Age-standardised incidence rate (95% UI)**	**Age-standardised mortality rate (95% UI)**	**Age-standardised DALY rate (95% UI)**
Global	2 166 168 (1 996 298–2 342 842)	1 085 797 (1 002 795–1 149 679)	24 284 087 (22 614 920–25 723 221)	26·7 (24·6–28·9)	13·7 (12·6–14·5)	295·5 (275·2–313·0)
Andean Latin America	11 094 (8935–13 467)	5630 (4593–6791)	125 578 (101 753–151 796)	20·0 (16·1–24·2)	10·3 (8·4–12·4)	220·8 (179·0–266·3)
Australasia	23 671 (19 439–28 848)	8382 (7575–8978)	163 248 (150 872–173 959)	48·3 (39·6–59·1)	16·2 (14·8–17·3)	348·6 (324·2–370·7)
Caribbean	13 813 (11 813–15 959)	7995 (6935–9176)	172 016 (147 186–200 175)	26·7 (22·9–30·9)	15·5 (13·4–17·7)	333·3 (285·2–387·9)
Central Asia	10 949 (9999–12 008)	7467 (6822–8166)	199 841 (182 012–219 941)	15·2 (13·9–16·6)	11·2 (10·3–12·2)	256·8 (234·4–281·1)
Central Europe	84474 (74 551–95 453)	51 567 (45 636–57 749)	1 052 146 (922 923–1 184 246)	39·9 (35·2–45·1)	23·6 (20·8–26·4)	512·6 (448·7–577·9)
Central Latin America	37 542 (32 211–43 870)	22 470 (19 542–25 997)	539 638 (465 200–627 069)	15·9 (13·7–18·6)	9·7 (8·4–11·2)	223·7 (193·1–259·5)
Central sub-Saharan Africa	3957 (3015–5113)	3544 (2705–4609)	100 988 (75 749–131 447)	7·7 (5·9–10·1)	7·4 (5·7–9·9)	169·3 (129·2–220·2)
East Asia	637 096 (548 895–738 549)	275 604 (238 238–317 886)	6 712 862 (5 774 277–7 735 907)	30·9 (26·8–35·7)	14·1 (12·2–16·2)	325·2 (280·7–373·2)
Eastern Europe	106 017 (96 250–117 074)	63 476 (57 180–70 011)	1 419 105 (1 287 540–1 571 374)	31·1 (28·2–34·4)	18·3 (16·5–20·2)	423·7 (384·0–469·3)
Eastern sub-Saharan Africa	14 227 (12 130–16 886)	12 717 (10 940–15 001)	356 433 (301 931–425 606)	8·8 (7·6–10·4)	8·5 (7·4–9·9)	193·9 (166·0–229·6)
High-income Asia Pacific	196 371 (166 417–225 643)	76 929 (64 821–83 603)	1 327 823 (1 186 117–1 414 814)	44·6 (38·4–51·1)	15·3 (13·4–16·4)	323·9 (298·6–342·1)
High-income North America	260 911 (229 909–295 693)	95 664 (88 321–99 688)	1 987 109 (1 895 869–2 059 774)	42·7 (37·6–48·6)	14·9 (13·9–15·5)	339·9 (325·9–351·9)
North Africa and Middle East	60 010 (53 354–67 555)	39 147 (34 761–44 107)	1 013 634 (896 161–1 146 526)	13·9 (12·3–15·6)	9·8 (8·7–11)	218·7 (194·1–246·5)
Oceania	691 (555–855)	551 (443–682)	16 315 (12 915–20 556)	10·0 (8·2–12·1)	8·8 (7·2–10·7)	203·6 (163·6–252·5)
South Asia	113 711 (98 190–129 352)	94 846 (81 524–109 075)	2 419 098 (2 078 019–2 782 570)	8·3 (7·2–9·4)	7·3 (6·2–8·3)	165·1 (141·7–189·9)
Southeast Asia	117 010 (96 631–136 244)	82 024 (67 617–94 606)	2 142 434 (1 780 490–2 482 287)	19·3 (16–22·4)	14·4 (11·9–16·6)	334·0 (276·6–386·4)
Southern Latin America	26 866 (21 480–33 612)	17 930 (16 774–18 975)	366 436 (347 729–385 441)	32·2 (25·7–40·4)	21·2 (19·9–22·4)	447·6 (424·7–470·5)
Southern sub-Saharan Africa	7106 (6389–7882)	5922 (5329–6580)	147 780 (132 439–165 539)	13·1 (11·8–14·5)	11·5 (10·4–12·7)	250·4 (225·1–279·3)
Tropical Latin America	42 891 (40 118–44 928)	27 704 (25 668–29 090)	660 129 (625 562–687 740)	17·8 (16·6–18·6)	11·7 (10·8–12·3)	268·3 (253·7–279·8)
Western Europe	382 442 (332 800–432 448)	172 454 (155 345–181 815)	3 008 234 (2 815 060–3 152 895)	42·4 (37·1–48·3)	17·3 (15·8–18·1)	351·2 (332·0–366·8)
Western sub-Saharan Africa	15 321 (12 895–17 824)	13 773 (11 698–16 069)	353 242 (295 571–420 704)	8·7 (7·4–10·0)	8·4 (7·3–9·7)	176·1 (149·0–206·2)

Numbers in parenthesis represent 95% uncertainty intervals (UIs). DALYs=disability-adjusted life years. The age-standardised incidence rate, age-standardised mortality rate, and age-standardised DALY rate are shown per 100 000 person-years. Source: Global Burden of Diseases, Injuries, and Risk Factors Study 2019.

**Figure 2:**
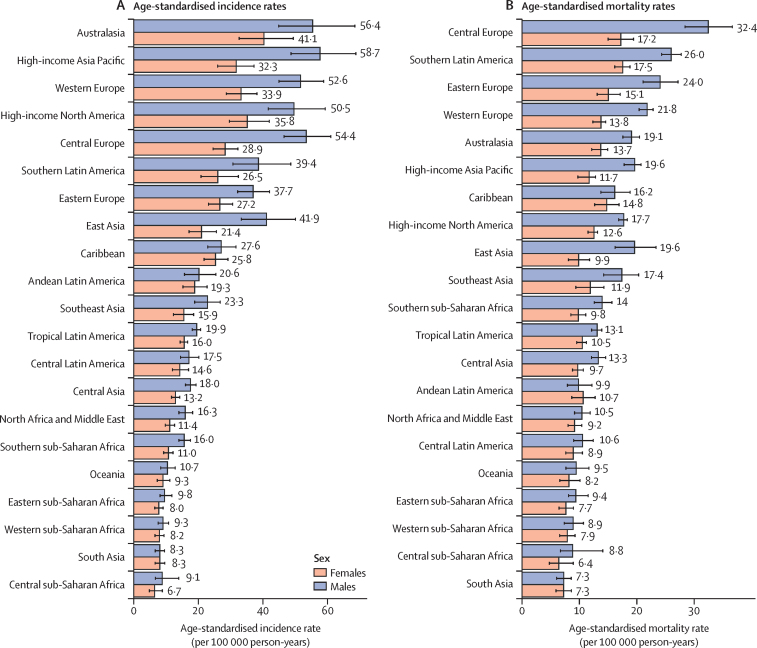
Age-standardised rates of colorectal cancer in 2019, by sex and region (A) Age-standardised incidence rate (per 100 000 person-years). (B) Age-standardised mortality rate (per 100 000 person-years). Error bars denote 95% uncertainty intervals. Data source: Global Burden of Diseases, Injuries, and Risk Factors Study 2019.

The preponderance of colorectal cancer in males in 2019, in terms of both age-standardised incidence rates and age-standardised mortality rates, was more apparent in developed regions (eg, Australasia, central Europe, and the high-income Asia Pacific) and differences between males and females were smaller in south Asia and regions of Africa (eg, eastern sub-Saharan Africa and western sub-Saharan Africa; figure 2).

Three patterns emerged from region-wise temporal trends of age-standardised rates in GBD regions (appendix p 9). First, in regions with already high incidence rates in 1990 (eg, Australasia and the high-income Asia Pacific), the age-standardised incidence rates either stagnated or decreased and age-standardised mortality rates decreased in the past three decades. Second, concomitant rises in age-standardised incidence and mortality rates were observed in a few regions of Asia, Africa (eg, western sub-Saharan Africa and south Asia), and Oceania. Third, large increases occurred in age-standardised incidence rates, whereas a smaller increase was observed in age-standardised mortality rates in a few regions (eg, east Asia and south east Asia).

**Figure 3:**
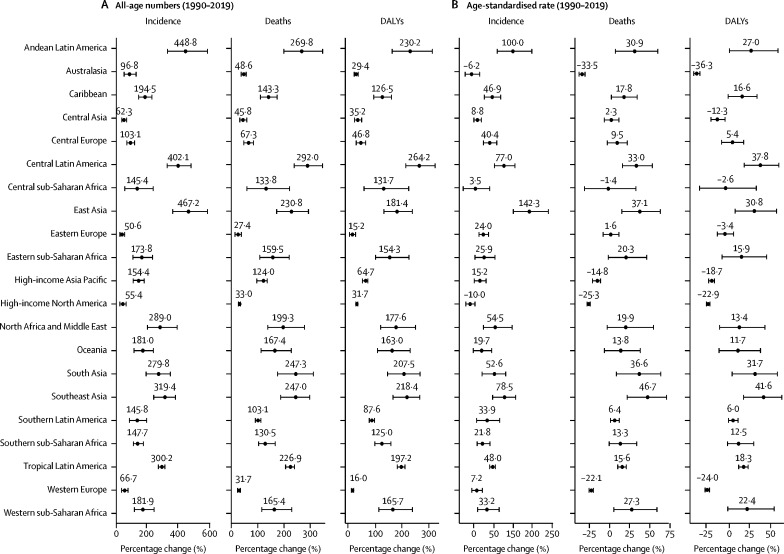
Region-wise percentage change in colorectal cancer burden, 1990–2019 (A) All-age numbers. B) Age-standardised rate (per 100 000). Data source: Global Burden of Diseases, Injuries, and Risk Factors Study 2019. Error bars denote 95% uncertainty intervals. DALYs=disability-adjusted life-years.

From 1990 to 2019, incident cases doubled or more than doubled in 16 of 21 regions, deaths doubled or more than doubled in 15 of 21 regions, and DALYs doubled or more than doubled in 13 of 21 regions, led by regions of Latin America and Asia (figure 3A). The changes in age-standardised rates were modest, with age-standardised incidence rates increasing by 50% or more in six GBD regions (east Asia, Andean Latin America, southeast Asia, central Latin America, North Africa and the Middle East, and south Asia; figure 3B). Age-standardised incidence rates decreased, although these decreases were not statistically significant, in high-income North America (−10·0% [95% UI −21·1 to 2·5]) and Australasia (−6·2% [–22·7 to 14·7]) between 1990 and 2019. Age-standardised mortality rates increased the most in southeast Asia (46·7% [21·8 to 70·0]) followed by east Asia (37·1% [15·1 to 62·3]), and decreased in Australasia (−33·5% [–29·8 to −37·2]) and high-income North America (−25·3% [–23·5 to −27·3]) from 1990 through 2019, with a couple of other high-income regions experiencing a reduction (−22·1% [–25·0 to −19·6] in western Europe and −14·8% [–20·6 to −10·9] in the high-income Asia Pacific). Due to large increases in incidence and death rates, the age-standardised DALY rate increased the most in regions of Asia and Latin America, led by southeast Asia (41·6% [18·2 to 62·7]) and central Latin America (37·8% [19·0 to 59·4]) and decreased in high-income regions and regions of Europe (figure 3B).

### Colorectal cancer burden by country

**Figure 4:**
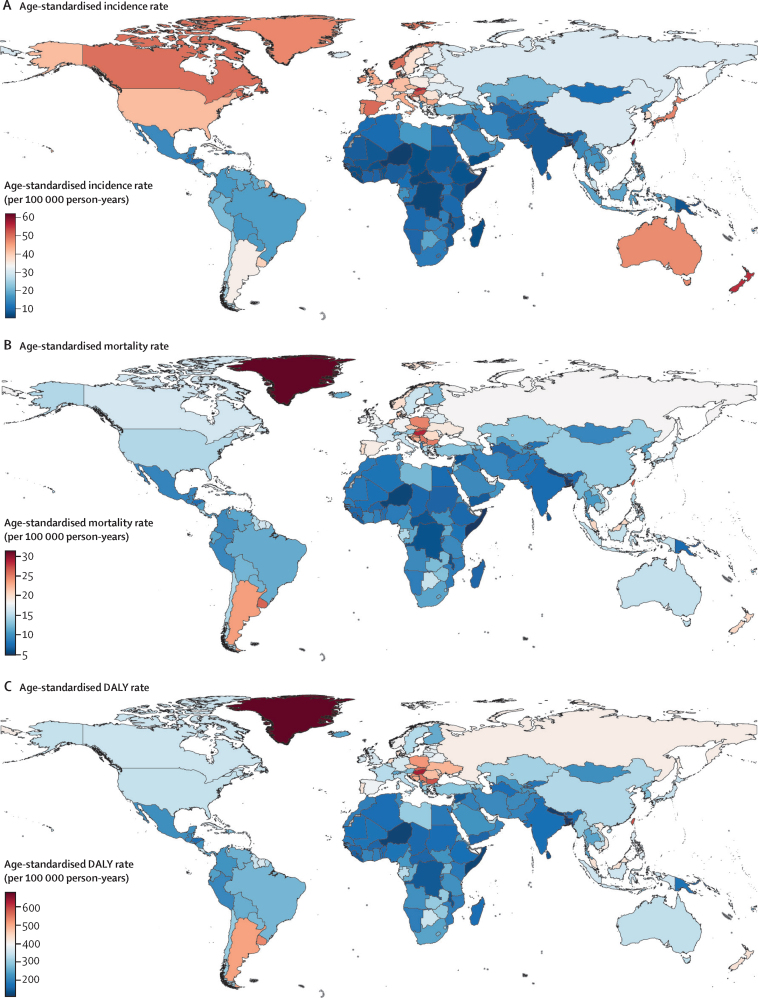
Geographical distribution of age-standardised rates of colorectal cancer in 2019 (A) Age-standardised incidence rate. (B) Age-standardised mortality rate. (C) Age-standardised DALY rate. Data source: Global Burden of Diseases, Injuries, and Risk Factors Study 2019. DALY=disability-adjusted life-year.

China, the USA, and Japan had the highest all-age incident counts for both sexes combined, with 607 900 (95% UI 521 805–708 420) new cases in China, 227 242 (197 022–261 375) new cases in the USA, and 160 211 (130 730–186 831) new cases in Japan, in 2019 (appendix pp 18–43). China (261 777 [224 403–303 318]), India (79 098 [67 137–92 723]), and the USA (84 026 [77 987–87 516]) had the highest death counts. Somalia (5·0 [3·1–9·2] per 100 000), Niger (5·6 [4·2–7·6] per 100 000), and Bangladesh (5·6 [3·9–8·0] per 100 000) had the lowest age-standardised incidence rates, whereas Taiwan (province of China; 62·0 [48·9–80·0] per 100 000), Monaco (60·7 [48·5–73·6] per 100 000), and Andorra (56·6 [42·8–71·9] per 100 000) had the highest age-standardised incidence rates (figure 4A; appendix pp 18–43). Greenland (31·4 [26·0–37·1] per 100 000), Brunei (30·3 [26·6–34·1] per 100 000), and Hungary (28·6 [23·6–34·0] per 100 000) had the highest age-standardised mortality rates, whereas Bangladesh (4·9 [3·4–7·1] per 100 000), Somalia (5·0 [3·2–9·3]), and Nepal (5·4 [3·9–7·4] per 100 000) had the lowest age-standardised mortality rates among 204 countries and territories in 2019 (figure 4B; appendix pp 18–43). Age-standardised DALY rates varied from 107·4 (74·6–152·7) per 100 000 in Bangladesh to 680·3 (555·7–812·4) per 100 000 in Greenland in 2019 (figure 4C; appendix pp 18–43).

Between 1990 and 2019, for both sexes combined, incident cases doubled or more than doubled in 157 of 204 countries and territories and deaths doubled or more than doubled in 129 of 204 countries and territories (appendix pp 44–66). Austria was the only country that reported a significant reduction in the number of colorectal cancer deaths (−20·5% [–26·4 to −14·8]) and DALYs (−29·7% [–34·4 to −25·0]) between 1990 and 2019. The changes in age-standardised rates were modest across countries in comparison with changes in absolute counts. From 1990 through 2019, the largest increase in age-standardised rates occurred in Cape Verde (age-standardised incidence rate 180·6% [121·1 to 237·3], age-standardised mortality rate 152·9% [97·3 to 204·0], and age-standardised DALY rate 114·2% [71·8 to 159·4]) and the largest reduction occurred in Austria (age-standardised incidence rate −34·1% [–46·7 to −19·1], age-standardised mortality rate −50·2% [–53·3 to −46·8], and age-standardised DALY rate −53·2% [–56·2 to −50·0]; appendix pp 44–66).

The relationship between country-level age-standardised rates of colorectal cancer and SDI in 2019 is shown in the appendix (p 10). SDI seemed to exert a positive relationship with age-standardised rates, with the slope becoming steeper in the upper end of the development spectrum for age-standardised incidence rates, whereas a positive relationship between age-standardised DALY rates and SDI seemed to taper off slightly towards high SDI countries.

### Colorectal cancer burden by age group

**Figure 5:**
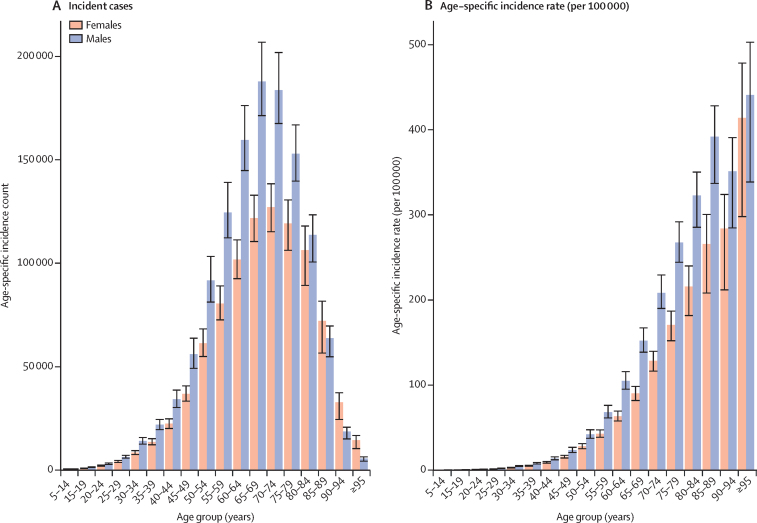
Age-specific burden of colorectal cancer in 2019 (A) Incident cases. (B) Age-specific incidence rate (per 100 000). Error bars denote 95% uncertainty intervals. Data source: Global Burden of Diseases, Injuries, and Risk Factors Study 2019.

Figure 5 illustrates the colorectal cancer incidence count and age-specific rates (per 100 000 person-years) in 2019. The incidence count followed a bell-shaped distribution with a peak in individuals aged 60–74 years for both males and females. Incident cases were higher in males than in females in all age groups up to age 80–84 years, with a greater number of new cases in females aged 85 years and older (figure 5A). Unlike incident cases, incidence rates continued to increase with age, increasing much faster in those aged 50–54 years and older (figure 5B). Between 1990 and 2019, all age groups experienced a rise in incident cases, with the highest increases occurring in those aged 85 years and older (appendix p 11). Furthermore, incidence rates increased in younger age groups (20–49 years) and decreased in older age groups (50–80 years) only in the high SDI quintile (appendix p 12). Percentage changes in incidence rates were higher in females compared to males only in low SDI quintiles; in all other SDI quintiles, males had higher percentage changes in incidence rates. Moving up in the development spectrum, the contrast between increases (or decreases) in younger age groups (20–49 years) and older age groups (50–74 years) became more pertinent. For instance, in the high SDI quintile, the incidence rates either remained unchanged or decreased in older age groups (50–74 years), whereas large increases were observed in younger age groups (20–49 years; appendix p 12).

### Risk factors

**Figure 6:**
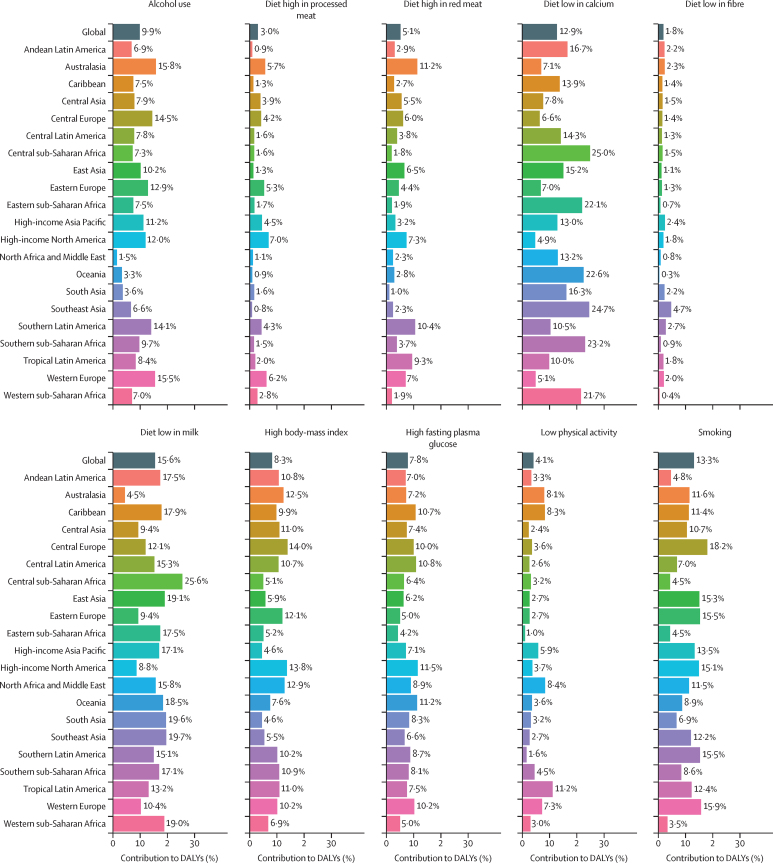
Percentage contribution of risk factors to all-age DALYs of colorectal cancer in 2019, for both sexes, globally and by regions Data source: Global Burden of Diseases, Injuries, and Risk Factors Study 2019. DALYs=disability-adjusted life-years.

Figure 6 depicts the contribution of ten risk factors to all-age DALYs due to colorectal cancer, for both sexes combined, for 21 GBD regions in 2019. At the global level, a diet low in milk (15·6%), smoking (13·3%), a diet low in calcium (12·9%), and alcohol use (9·9%) were the main contributors to colorectal cancer DALYs, with the relative contribution of different risk factors varying as per the region's development status. In sub-Saharan Africa and Asia (barring the high-income Asia Pacific), a diet low in calcium and milk were the main risk factors, whereas smoking and alcohol use were the main risk factors in high-income regions (figure 6). At the global level, high BMI contributed only 8·3% of DALYs, with a higher contribution in comparatively high-income regions (eg, central Europe [14·0%] and high-income North America [13·8%]). The contribution of smoking to DALYs was greater than 15% in regions of Europe (eg, 18·2% in central Europe, 15·9% in western Europe, and 15·5% in eastern Europe) as well as in southern Latin America, east Asia, and high-income North America.

The severity of risk factors also varied by sex, with alcohol use contributing 13·9% of global DALYs and smoking contributing 18·9% of global DALYs in males, whereas alcohol use contributed only 4·5% of global DALYs and smoking contributed only 5·7% of global DALYs in females (appendix p 13). In females, a diet low in milk (15·5%), a diet low in calcium (12·0%), and high fasting plasma glucose (7·5%) were the major contributors to colorectal cancer DALYs. Similar differences were observed between sexes with regard to the contribution of high BMI, which contributed 11·1% of DALYs in males and 4·6% in females. The differences between sexes in the contribution of risk factors to colorectal cancer DALYs were also apparent in all GBD regions. The distribution of inadequate dietary risk factors (eg, a diet low in milk and calcium) was similar across the two sexes across regions of sub-Saharan Africa and Asia (appendix p 13).

## Discussion

Between 1990 and 2019, new cases of colorectal cancer doubled or more than doubled in 157 of 204 countries and territories, and deaths due to colorectal cancer doubled or more than doubled in 129 of 204 countries and territories, and large increases were experienced in low SDI and middle SDI countries. Incidence and mortality rates mostly decreased in high SDI countries, whereas several low SDI and middle SDI countries and regions saw increases in age-standardised rates. The incident cases shared by low SDI, low-middle SDI, middle SDI, and high-middle SDI countries increased from 47·3% to 62·1%, the share of death counts rose from 57·1% to 69·8%, and the share of DALYs increased from 62·4% to 74·6% from 1990 through 2019.

We found large increases in age-standardised incidence rates in Asia (eg, 142·3% in east Asia and 78·5% in southeast Asia) and Latin America (eg, 100·0% in Andean Latin America, and 77·0% in central Latin America) between 1990 and 2019. Due to fast economic growth and rapid industrialisation, the thriving middle class in developing countries is adopting a westernised lifestyle characterised by an unhealthy diet (low in fruits and vegetables and high in red meat and processed meat), sedentary behaviour (eg, spending a long time watching television), and less physical activity, along with substance abuse (alcohol and smoking).^19^, ^20^ These behavioural changes have resulted in an increased incidence of lifestyle-related illnesses, including colorectal cancer.

The age-standardised mortality rate decreased in high-income regions (eg, −33·5% [95% UI −29·8 to −37·2] in Australasia and −25·3% [–23·5 to −27·3] in high-income North America) between 1990 and 2019. The reduction in incident cases and deaths, particularly among individuals older than 50 years, in high-SDI regions and countries suggests early detection of colorectal cancer due to screening, cancer registries, and technological improvements, as well as normalisation of early referral to physicians. In most high SDI countries, 50–75 years is also the age group in which colorectal cancer screening is generally recommended for early detection of adenomatous polyps and adenomas.^21^ In the USA, the impact of colorectal cancer screening in reduction of deaths due to colorectal cancer is notable given the considerable increases in colonoscopy screening in the 2000s.^22^

We found more colorectal cancer cases and deaths among males than females, with male age-standardised rates being 1·5–2·0-times higher than females in different GBD regions and the difference between males and females growing over time. In the 1960s, when the first international reports were published on colorectal cancer, the age-standardised incidence rate used to be higher for females than males, but the incidence has since risen faster for males than for females.^23^ The predominance of males in the global colorectal cancer burden has been attributed to the high prevalence of visceral fat,^24^ higher smoking prevalence,^19^ and greater alcohol consumption among this demographic.^25^ We also found a greater contribution of alcohol and smoking to colorectal cancer DALYs in males than in females. Some studies have also suggested protective effects of endogenous oestrogen against colorectal carcinogenesis in females.^26^ Besides endogenous oestrogens, oral contraceptives might also contribute to the lowered colorectal cancer risk in females compared to males.^27^ Besides the difference in incidence, there are also differences in mortality between males and females, with females tending to have better survival outcomes.^28^, ^29^ Sex apparently modulates the circadian clocks of males and females differently, with respect to the effects of chemotherapy in colorectal cancer, which might also contribute to the sex-based differences in survival and mortality.^30^ In terms of the age-specific burden of colorectal cancer in males versus females, the findings of GBD 2019 differ from a previous study in which colorectal cancer incidence and mortality were higher in females than in males in individuals aged 65 years and older, implying that colorectal cancer is a major health threat in older females;^31^ in the present study, however, such differences were mainly observed in individuals aged 80 years and older.

In line with previous studies,^32^, ^33^, ^34^, ^35^, ^36^ we observed large increases in new cases and age-specific rates in individuals aged 20–49 years between 1990 and 2019, especially in high SDI countries. In the USA, a previous study^37^ also noted substantial increases in colorectal cancer incidence rates in individuals aged 20–49 years from 1975 to 2010 and the incidence rate of colon cancers is expected to increase by an estimated 90%, and the incidence rate of rectal cancers is expected to increase by 140%, by 2030.^37^, ^38^ In most high-income countries, screening is recommended from the age of 50 years onwards; however, the recent trends in growing colorectal cancer incidence in younger adults (<50 years) have led to calls for a reconsideration of screening recommendations to include individuals aged 40–49 years. In 2019, the American Cancer Society recommended colorectal cancer screening from the age of 45 years onwards,^36^ which can be further modified to include younger people, especially targeting those with a high risk of colorectal cancer (eg, personal history of polyps or adenoma or family history of colorectal cancer or hereditary risk, males, smokers, and those with a high BMI).

The exact reasons for the increasing incidence of colorectal cancer in people younger than 50 years are less clear, but one possible reason could be due to the birth cohort effect, such that those born in the second half of the 20th century are increasingly exposed to potentially modifiable behavioural risk factors such as unhealthy diets, obesity, sedentary behaviour, low physical activity,^18^ and increased smoking prevalence in early adulthood.^32^, ^33^, ^39^, ^40^ However, most of these risk factors have been implicated on the basis of evidence generated from patients with colorectal cancer aged 50 years or older, so the exact mechanisms or underlying risk factors remain less clear.^40^, ^41^, ^42^ A previous study has attributed prolonged sedentary television viewing time to early onset of colorectal cancer, particularly rectal cancer.^43^ Early-onset colorectal cancer has also been attributed to the rising obesity prevalence in younger adults.^44^, ^45^, ^46^ A prospective cohort study of 94 217 women showed that dietary and lifestyle factors leading to hyperinsulinaemia are associated with an increased risk of colorectal cancer in younger women in the USA.^47^ Binge drinking (episodic heavy alcohol consumption) is also implicated as one of the risk factors, with binge drinking being higher among adults younger than 50 years than in adults aged 50 years and older (ie, those of screening age).^48^

The GBD 2019 colorectal cancer estimates (2·17 million incident cases and 1·09 million deaths) are higher than GLOBOCAN estimates for 2020 (1·9 million incident cases and 935 173 deaths).^49^ The global age-standardised rates estimated by GBD are also higher (age-standardised incidence rate 26·7 per 100 000; age-standardised mortality rate 13·7 per 100 000) than those for GLOBOCAN (age-standardised incidence rate 19·5 per 100 000 and age-standardised mortality rate 9·0 per 100 000). Both GBD and GLOBOCAN report high incidence and mortality rates in high-income regions and countries in Europe, North America, and Asia, and low incidence and mortality rates in LMICs of sub-Saharan Africa and south Asia, yet there are a few differences in the estimates, which stem from the different methodologies and data sources used. One of the main differences is that GLOBOCAN mostly estimates cancer incidence and deaths from cancer registry data, whereas GBD also models estimates from data sources such as vital registration, cancer registries, verbal autopsy, and sample registration systems. Another key difference between GBD and GLOBOCAN estimates stems from the redistribution of deaths from unknown or non-specific cancers to other cancers in GBD estimates.^15^ The GBD estimation framework has two key advantages over GLOBOCAN. In addition to producing incidence and mortality estimates, GBD produces estimates for YLLs, YLDs, and DALYs, which encompasses the disease burden due to both death and disability caused by the disease. Second, GLOBOCAN provides estimates for a single year (eg, 2002, 2008, 2012, 2018, and 2020) and there are some time series available for selected countries up to 2012 (the CI5Plus: Cancer Incidence in Five Continents Time Trends series); however, continuous time series of estimates of the colorectal cancer burden are not available from GLOBOCAN for sufficiently long time periods at the global and regional levels for all countries and territories. A track of temporal patterns can provide useful information; for instance, the rises in colorectal cancer incidence among younger adults (<50 years) in the past three decades, particularly in high-income regions, can allow researchers to examine the changing risk factors, set hypotheses, and help clinicians and policy makers to be more vigilant about the changing trends of colorectal cancer.

The major limitation of this study was the non-availability of data from cancer registries in many countries in Africa, the Caribbean, and Asia. Although GBD uses all available data from sources such as vital registration, verbal autopsy, and cancer registries, the accuracy of GBD estimates crucially depends upon cancer incidence and mortality data from cancer registries. Many LMICs in sub-Saharan Africa and Asia either do not have population-based cancer registries or the existing registries have insufficient coverage. Under-reporting also occurs because of poor documentation of cases, a shortage of medical facilities and adequately trained medical personnel, lack of a well established oncology centre, and as a result of misdiagnosis. Moreover, the 95% UIs reported in the study do not take into account several sources of bias, including measurement bias, selection bias due to missing data, and model specification bias. Second, the disease registration system was inadequate 30 years ago, especially in LMICs, and although it has improved in several LMICs in recent years, the estimates for the early years of the analysis (ie, the 1990s) are expected to be more biased, which is also reflected in the wider 95% UIs of the mean estimates and percentage changes. Third, there is a lag in the official reporting of cancer data for 2019 even in high SDI countries so the estimates for the most recent years are generated on the basis of recent trends and are provided with wider uncertainty intervals. GBD is an iterative estimation framework providing temporal estimates; the official data for 2019 will be updated as they become available and will be reflected in future GBD iterations.

In conclusion, colorectal cancer incident cases and deaths have more than doubled worldwide in the past three decades. Reducing the prevalence of modifiable risk factors and increasing screening uptake are key to reducing deaths from colorectal cancer, in line with achieving target 3.4 of the SDGs.^8^ Low SDI and middle SDI (including low-middle, middle, and high-middle SDI) countries, which together comprise close to 75% of colorectal cancer DALYs, are expected to experience further increases in colorectal cancer incidence as a result of population ageing and increasing life expectancy, improved screening and detection, and changing lifestyles. Strategies such as dietary and lifestyle modifications, early screening among high-risk individuals, access to high-quality health care, and better treatment modalities (including improved personalised therapy and access to clinical trials) are imperative to face this global challenge. Population-based cancer registries are important for monitoring colorectal cancer incidence and survival. The increase in colorectal cancer incidence in people younger than 50 years should serve as an early warning signal, necessitating greater awareness of these trends among clinicians, researchers, and policy makers, as well as more research into the risk factors and mechanisms that underpin these trends.

The data generated through GBD are an important resource for people and health-care providers, as they provide information on the effect of current treatment strategies, the effects of previous interventions, and the need for preventive measures. The findings from GBD 2019 can be used by policy makers and provide new perspectives for scientists and physicians throughout the world. These results provide comprehensive and comparable estimates that can inform efforts for equitable colorectal cancer control worldwide, with the larger goal of reducing the global burden of cancer.

## Data sharing

To download the data used in these analyses, please visit the Global Health Data Exchange GBD 2019 results website.

## Declaration of interests

R Ancuceanu reports consulting fees from AbbVie; payment or honoraria for lectures, presentations, speaker's bureaus, manuscript writing, or educational events from AbbVie, Sandoz, and B Braun; all outside the submitted work. M Ausloos reports grants from the Romanian National Authority for Scientific Research and Innovation, CNDS-UEFISCDI, project number PN-III-P4-ID-PCCF-2016-0084 “Understanding and modelling time-space patterns of psychology-related inequalities and polarization” (October, 2018, to September, 2022), outside the submitted work. J Conde reports grants from the European Research Council Starting Grant (ERC-StG-2019-848325); patents planned, issued or pending for functionalised nanoparticles and compositions for cancer treatment and methods (US Application No. 62/334538), and TRPV2 Antagonists WO Application No. PCT/PT2018/050035; all outside the submitted work. I Fillip reports consulting fees from Avicenna Medical and Clinical Research Institute, outside the submitted work. N Ghith reports grants from Novo Nordisk Foundation as salary payment (NNF16OC0021856), outside the submitted work. A Guha reports grants from the American Heart Association as the Strategically Focused Research Network Grant in Disparities in Cardio-Oncology (#847740 and #863620), outside the submitted work. C Herteliu and A Pana report grants or contracts from Romanian National Authority for Scientific Research and Innovation, CNDS-UEFISCDI, project number PN-III-P4-ID-PCCF-2016-0084 (October, 2018, to September, 2022) “Understanding and modelling time-space patterns of psychology-related inequalities and polarization” and Project number PN-III-P2-2·1-SOL-2020-2-0351 (June to October, 2020) “Approaches within public health management in the context of COVID-19 pandemic”, all outside the submitted work. C Herteliu reports grants from the Ministry of Labour and Social Justice, Romania, project number 30/PSCD/2018, “Agenda for skills Romania 2020–2025;” outside the submitted work. J Jozwiak reports payment or honoraria for lectures, presentations, speaker's bureaus, manuscript writing, or educational events from Teva, Amgen, Synexus, Boehringer Ingelheim, Alab Laboratories, and Zentiva as personal fees, all outside the submitted work. J H Kauppila reports grants from the Sigrid Juselius Foundation and Finnish Cancer Foundation as research grants paid to their institutions; all outside the submitted work. J A Loureiro reports support for the present manuscript from Fundação para a Ciência e Técnologia (FCT) as a salary payment under the Scientific Employment Stimulus (CEECINST/00049/2018) and from FCT/MCTES (Ministério da Ciência, Tecnologia e Ensino Superior) (PIDDAC) as Base Funding (UIDB/00511/2020 of LEPABE). A-F A Mentis reports grants or contracts from ELIDEK (Hellenic Foundation for Research and Innovation, MIMS-860) and EPANEK - MilkSafe (Τ2ΕΔΚ-02222), all outside the submitted manuscript. O O Odukoya reports support for the present manuscript from the Fogarty International Center of the National Institutes of Health under the award number K43TW010704. The content is solely the responsibility of the authors and does not necessarily represent the official views of the National Institutes of Health. A Radfar reports consulting fees from Avicenna Medical and Clinical Research Institute; leadership or fiduciary role in other board, society, committee, or advocacy group, paid or unpaid with MEDICHEM as a board member; all outside the submitted work. M Šekerija reports payment or honoraria for lectures, presentations, speaker's bureaus, manuscript writing, or educational events from Roche and Johnson & Johnson, outside the submitted work. D A S Silva reports support for the present manuscript in part from the Coordenação de Aperfeiçoamento de Pessoal de Nível Superior—Brazil (CAPES)—Finance Code 001 and in part by Conselho Nacional de Desenvolvimento Científico e Tecnológico, Brazil (CNPq - 302028/2018-8), as payments made to their institution. J A Singh reports consulting fees from Crealta/Horizon, Medisys, Fidia, Two labs, Adept Field Solutions, Clinical Care Options, ClearView Healthcare Partners, Putnam Associates, Focus Forward, Navigant Consulting, Spherix, MedIQ, UBM, Trio Health, Medscape, WebMD, and Practice Point Communications; and the National Institutes of Health and the American College of Rheumatology; payment or honoraria for lectures, presentations, speakers bureaus, manuscript writing, or educational events from Simply Speaking; support for attending meetings or travel, or both from OMERACT, an international organisation that develops measures for clinical trials and receives arm's length funding from 12 pharmaceutical companies, when traveling to OMERACT meetings; participation on a data safety monitoring board or advisory board as a member of the FDA Arthritis Advisory Committee; leadership or fiduciary role in other board, society, committee or advocacy group, paid or unpaid, with OMERACT as a member of the steering committee, with the Veterans Affairs Rheumatology Field Advisory Committee as a member, and with the UAB Cochrane Musculoskeletal Group Satellite Center on Network Meta-analysis as a director and editor; stock or stock options in TPT Global Tech, Vaxart Pharmaceuticals and Charlotte's Web Holdings, and previously owned stock options in Amarin, Viking, and Moderna Pharmaceuticals; all outside the submitted work. M Solmi reports payment or honoraria for lectures, presentations, speaker's bureaus, manuscript writing, or educational events from Lundbeck; and participation on a data safety monitoring board or advisory board with Angelini; all outside the submitted work. T Vos reports support for the present manuscript from the Bill & Melinda Gates Foundation as payment to their institution. All other authors declare no competing interests.
