# Correction to *Lancet Gastroenterol Hepatol* 2022; 7: 627–47

**DOI:** 10.1016/S2468-1253(22)00210-2

**Published:** 2022-07-06

*GBD 2019 Colorectal Cancer Collaborators. Global, regional, and national burden of colorectal cancer and its risk factors, 1990–2019: a systematic analysis for the Global Burden of Disease Study* 2019. Lancet Gastroenterol Hepatol *2022; **7:** 627–47*—The affiliation details for Mihajlo Jakovljevic have been corrected online as of July 6, 2022.

