# Supplementary material for: Global, regional, and national burden of colorectal cancer and its risk factors, 1990–2019: a systematic analysis for the Global Burden of Disease Study 2019
Source: Lancet Gastroenterol Hepatol. 2022 Apr 7;7(7):627–47. doi: 10.1016/S2468-1253(22)00044-9 (PMC9192760; doi:10.1016/S2468-1253(22)00044-9)
Supplement: Supplementary appendix [file mmc1.pdf]

THE LANCET
Gastroenterology & Hepatology

Supplementary appendix

This appendix formed part of the original submission and has been peer reviewed. We post it as supplied by the authors.

Supplement to: GBD 2019 Colorectal Cancer Collaborators. Global, regional, and national burden of colorectal cancer and its risk factors, 1990–2019: a systematic analysis for the Global Burden of Disease Study 2019. *Lancet Gastroenterol Hepatol* 2022; published online April 7. https://doi.org/10.1016/S2468-1253(22)00044-9.

Managing the estimation or publications process
Rajesh Sharma

Writing the first draft of the manuscript
Rajesh Sharma

Primary responsibility for applying analytical methods to produce estimates
Rajesh Sharma

Primary responsibility for seeking, cataloguing, extracting, or cleaning data; designing or coding figures and tables
Rajesh Sharma

Providing data or critical feedback on data sources
Rami Abd-Rabu, Hassan Abidi, Sangeet Adhikari, Shailesh M Advani, Bright Opoku Ahinkorah, Sajjad Ahmad, Ali Ahmadi, Sepideh Ahmadi, Muktar Beshir Ahmed, Hanadi Al Hamad, Fares Alahdab, Fahad Mashhour Alanezi, Turki M Alanzi, Syed Mohamed Aljunid, Motasem Alkhayyat, Sami Almustanyir, Nelson Alvis-Guzman, Saeed Amini, Jalal Arabloo, Seyyed Shamsadin Athari, Floriane Ausloos, Marcel Ausloos, Ashish D Badiye, Sara Bagherieh, Saeed Bahadory, Atif Amin Baig, Maciej Banach, Amadou Barrow, Alemshet Yirga Berhie, Akshaya Srikanth Bhagavathula, Vijayalakshmi S Bhojaraja, Sadia Bibi, Belay Boda Abule Bodicha, Dejana Braithwaite, Daniela Calina, Raja Chandra Chakinala, William C S Cho, Dinh-Toi Chu, Xiaochen Dai, Lalit Dandona, Rakhi Dandona, Rupak Desai, Mostafa Dianatinasab, Mojtaba Didehdar, Saeid Doaei, Linh Phuong Doan, Milad Dodangeh, Fatemeh Eghbalian, Michael Ekholuenetale, Ildar Ravisovich Fakhradiyev, Hossein Farrokhpour, Farshad Farzadfar, Ali Fatehizadeh, Getahun Fetensa, Irina Filip, Peter Andras Gaal, Mohamed M Gad, Tushar Garg, Ahmad Ghashghaee, Jamshid Gholizadeh Navashenaq, Abraham Tamirat Gizaw, James C Glasbey, Mahaveer Golechha, Pouya Goleij, Sapna Gupta, Veer Bala Gupta, Vivek Kumar Gupta, Nima Hafezi-Nejad, Arvin Haj-Mirzaian, Shafiul Haque, Ahmed I Hasaballah, Soheil Hassanipour, Claudiu Herteliu, Mehdi Hosseinzadeh, Mowafa Househ, Mihajlo Jakovljevic, Tahereh Javaheri, Tamas Joo, Farahnaz Joukar, Jacek Jerzy Jozwiak, Rohollah Kalhor, Neeti Kapoor, Joonas H Kauppila, Yousef Saleh Khader, Himanshu Khajuria, Rovshan Khalilov, Hanna Kim, Min Seo Kim, Adnan Kisa, Sezer Kisa, Yuvaraj Krishnamoorthy, G Anil Kumar, Sang-woong Lee, Fariborz Mansour-Ghanaei, Lorenzo Giovanni Mantovani, Jitendra Kumar Meena, Entezar Mehrabi Nasab, Walter Mendoza, Alexios-Fotios A Mentis, Bartosz Miazgowski, Hamed Mirzaei, Karzan Abdulmuhsin Mohammad, Abdollah Mohammadian-Hafshejani, Shafiu Mohammed, Teroj Abdulrahman Mohammed, Nagabhishek Moka, Ali H Mokdad, Mariam Molokhia, Lorenzo Monasta, Ghobad Moradi, Joana Morgado-da-Costa, Sumaira Mubarik, Mohsen Naghavi, Mukhammad David Naimzada, Hae Sung Nam, Biswa Prakash Nayak, Javad Nazari, Ehsan Nazemalhosseini-Mojarad, Ionut Negoi, Cuong Tat Nguyen, Son Hoang Nguyen, Chimezie Igwegbe Nzoputam, Bogdan Oancea, Ayodipupo Sikiru Oguntade, Andrew T Olagunju, Tinuke O Olagunju, Sokking Ong, Adrian Pana, Anamika Pandey, Fatemeh Pashazadeh Kan, Uttam Paudel, Renato B Pereira, Zahra Zahid Piracha, Naeimeh Pourtaheri, Akila Prashant, Amir Radfar, Amir Masoud Rahmani, Vahid Rahmanian, Nazanin Rajai, Kiana Ramezanzadeh, Sina Rashedi, David Laith Rawaf, Salman Rawaf, Reza Rawassizadeh, Andre M N Renzaho, Nima Rezaei, Gholamreza Roshandel, Basema Saddik, Umar Saeed, Amir Salimi, Abdallah M Samy, Juan Sanabria, Milena M Santric-Milicevic, Arash Sarveazad, Brijesh Sathian, Maheswar Satpathy, Mario Šekerija, Allen Seylani, Feng Sha, Sayed Mohammad Shafiee, Saeed Shahabi, Javad Sharifi-Rad, Sara Sheikhbahaei, Jeevan K Shetty, Guilherme Silva Julian, Achintya Dinesh Singh, Jasvinder A Singh, Valentin Yurievich Skryabin, Anna Aleksandrovna Skryabina, Marco Solmi, Mohammad Sadegh Soltani-Zangbar, Viktória Szerencsés, Miklós Szócska, Rafael Tabarés-Seisdedos, Yasaman TaheriAbkenar, Amir Taherkhani, Amir Tiyuri,

Mathilde Touvier, Bach Xuan Tran, Irfan Ullah, Saif Ullah, Sahel Valadan Tahbaz, Bay Vo, Ronny Westerman, Melat Woldemariam, Seyed Hossein Yahyazadeh Jabbari, Chuanhua Yu, Deniz Yuce, Vesna Zadnik, Mazyar Zahir, Iman Zare, Zhi-Jiang Zhang, and Mohammad Zoladl.

Developing methods or computational machinery

Rami Abd-Rabu, Akshaya Srikanth Bhagavathula, Xiaochen Dai, Mostafa Dianatinasab, Saeid Doaei, Fatemeh Eghbalian, Ali Fatehizadeh, Rasool Haddadi, Mehdi Hosseinzadeh, Mowafa Househ, Tahereh Javaheri, Rovshan Khalilov, Adnan Kisa, Sang-woong Lee, Abdollah Mohammadian-Hafshejani, Teroj Abdulrahman Mohammed, Ali H Mokdad, Mohsen Naghavi, Ehsan Nazemalhosseini-Mojarad, Urvish K Patel, Zahra Zahid Piracha, Amir Masoud Rahmani, Vahid Rahmanian, Reza Rawassizadeh, Umar Saeed, Abdallah M Samy, Maheswar Satpathy, and Bay Vo.

Providing critical feedback on methods or results

Rami Abd-Rabu, Hassan Abidi, Eman Abu-Gharbieh, Juan Manuel Acuna, Sangeet Adhikari, Muhammad Sohail Afzal, Mohamad Aghaie Meybodi, Bright Opoku Ahinkorah, Sajjad Ahmad, Ali Ahmadi, Haroon Ahmed, Luai A Ahmed, Muktar Beshir Ahmed, Hanadi Al Hamad, Fares Alahdab, Fahad Mashhour Alanezi, Turki M Alanzi, Fadwa Alhalaiqa Naji Alhalaiqa, Yousef Alimohamadi, Vahid Alipour, Syed Mohamed Aljunid, Motasem Alkhayyat, Sami Almustanyir, Rajaa M Al-Raddadi, Saba Alvand, Nelson Alvis-Guzman, Saeed Amini, Alireza Ansari-Moghaddam, Jalal Arabloo, Armin Aryannejad, Mohammad Asghari Jafarabadi, Seyyed Shamsadin Athari, Floriane Ausloos, Marcel Ausloos, Mamaru Ayenew Awoke, Tegegn Mulatu Ayana, Sina Azadnajafabad, Hiva Azami, Mohammadreza Azangou-Khyavy, Amirhossein Azari Jafari, Ashish D Badiye, Sara Bagherieh, Atif Amin Baig, Jennifer L Baker, Maciej Banach, Amadou Barrow, Devidas S Bhagat, Akshaya Srikanth Bhagavathula, Krittika Bhattacharyya, Vijayalakshmi S Bhojaraja, Sadia Bibi, Ali Bijani, Antonio Biondi, Belay Boda Abule Bodicha, Dejana Braithwaite, Hermann Brenner, Daniela Calina, Chao Cao, Yin Cao, Giulia Carreras, Ester Cerin, Raja Chandra Chakinala, William C S Cho, Dinh-Toi Chu, Joao Conde, Vera Marisa Costa, Natália Cruz-Martins, Omid Dadras, Xiaochen Dai, Lalit Dandona, Rakhi Dandona, Feleke Mekonnen Demeke, Getu Debalkie Demissie, Rupak Desai, Deepak Dhamnetiya, Mostafa Dianatinasab, Daniel Diaz, Mojtaba Didehdar, Saeid Doaei, Linh Phuong Doan, Milad Dodangeh, Fatemeh Eghbalian, Debela Debela Ejeta, Michael Ekholuenetale, Temitope Cyrus Ekundayo, Iman El Sayed, Muhammed Elhadi, Daniel Berhanie Enyew, Tahir Eyayu, Ildar Ravisovich Fakhradiyev, Umar Farooque, Hossein Farrokhpour, Farshad Farzadfar, Ali Fatehizadeh, Hamed Fattahi, Nima Fattahi, Getahun Fetensa, Irina Filip, Florian Fischer, Masoud Foroutan, Peter Andras Gaal, Mohamed M Gad, Tushar Garg, Tamiru Getachew, Ahmad Ghashghaee, Nermin Ghith, Maryam Gholamalizadeh, Jamshid Gholizadeh Navashenaq, Abraham Tamirat Gizaw, James C Glasbey, Mahaveer Golechha, Kebebe Bekele Gonfa, Avirup Guha, Sapna Gupta, Veer Bala Gupta, Vivek Kumar Gupta, Rasool Haddadi, Nima Hafezi-Nejad, Rabih Halwani, Shafiul Haque, Sanam Hariri, Ahmed I Hasaballah, Soheil Hassanipour, Simon I Hay, Claudiu Herteliu, Ramesh Holla, Mohammad-Salar Hosseini, Mehdi Hosseinzadeh, Mihaela Hostiuc, Mowafa Househ, Ayesha Humayun, Olayinka Stephen Ilesanmi, Irena M Ilic, Milena D Ilic, Farhad Islami, Masao Iwagami, Mohammad Ali Jahani, Mihajlo Jakovljevic, Tahereh Javaheri, Ranil Jayawardena, Rime Jebai, Ravi Prakash Jha, Tamas Joo, Nitin Joseph, Farahnaz Joukar, Jacek Jerzy Jozwiak, Ali Kabir, Rohollah Kalhor, Ashwin Kamath, Neeti Kapoor, Ibraheem M Karaye, Amirali Karimi, Joonas H Kauppila, Mohammad Keykhaei, Yousef Saleh Khader, Himanshu Khajuria, Rovshan Khalilov, Javad Khanali, Maryam Khayamzadeh, Mahmoud Khodadost, Hanna Kim, Min Seo Kim, Adnan Kisa, Sezer Kisa, Hamid Reza Koohestani, Jacek A Kopec, Rajasekaran Koteeswaran, Ai Koyanagi, Yuvaraj Krishnamoorthy, G Anil Kumar, Manoj Kumar, Vivek Kumar, Carlo La Vecchia,

Faris Hasan Lami, Caterina Ledda, Sang-woong Lee, Wei-Chen Lee, Elvynna Leong, Bingyu Li, Stephen S Lim, Stany W Lobo, Joana A Loureiro, Farzan Madadizadeh, Ata Mahmoodpoor, Azeem Majeed, Mohammad-Reza Malekpour, Reza Malekzadeh, Ahmad Azam Malik, Fariborz Mansour-Ghanaei, Lorenzo Giovanni Mantovani, Miquel Martorell, Sahar Masoudi, Prashant Mathur, Jitendra Kumar Meena, Entezar Mehrabi Nasab, Walter Mendoza, Alexios-Fotios A Mentis, Tomislav Mestrovic, Junmei Miao Jonasson, Gelana Fekadu Worku Mijena, Seyyedmohammadsadeq Mirmoeeni, Mohammad Mirza-Aghazadeh-Attari, Sanjeev Misra, Karzan Abdulmuhsin Mohammad, Esmaeil Mohammadi, Saeed Mohammadi, Seyyede Momeneh Mohammadi, Abdollah Mohammadian-Hafshejani, Shafiu Mohammed, Teroj Abdulrahman Mohammed, Nagabhishek Moka, Ali H Mokdad, Sara Momtazmanesh, Ghobad Moradi, Rahmatollah Moradzadeh, Joana Morgado-da-Costa, Sumaira Mubarik, Mohsen Naghavi, Hae Sung Nam, Zuhair S Natto, Biswa Prakash Nayak, Javad Nazari, Ehsan Nazemalhosseini-Mojarad, Ionut Negoi, Cuong Tat Nguyen, Son Hoang Nguyen, Nurulamin M Noor, Maryam Noori, Chimezie Igwegbe Nzoputam, Bogdan Oancea, Oluwakemi Ololade Odukoya, Ayodipupo Sikiru Oguntade, Hassan Okati-Aliabad, Andrew T Olagunju, Tinuke O Olagunju, Samuel M Ostroff, Adrian Pana, Anamika Pandey, Fatemeh Pashazadeh Kan, Urvish K Patel, Uttam Paudel, Renato B Pereira, Richard G Pestell, Zahra Zahid Piracha, Richard Charles G Pollok, Akram Pourshams, Akila Prashant, Mohammad Rabiee, Navid Rabiee, Amir Radfar, Sima Rafiei, Mosiur Rahman, Amir Masoud Rahmani, Vahid Rahmanian, Nazanin Rajai, Aashish Rajesh, Vajiheh Ramezani-Doroh, Kamal Ranabhat, Sina Rashedi, Amirfarzan Rashidi, Mahsa Rashidi, Mohammad-Mahdi Rashidi, Mandana Rastegar, David Laith Rawaf, Salman Rawaf, Reza Rawassizadeh, Mohammad Sadegh Razeghinia, Andre M N Renzaho, Negar Rezaei, Nima Rezaei, Mohsen Rezaeian, Sahba Rezazadeh-Khadem, Gholamreza Roshandel, Bahar Saberzadeh-Ardestani, Basema Saddik, Hossein Sadeghi, Umar Saeed, Maryam Sahebazzamani, Amir Salimi, Hamideh Salimzadeh, Pouria Samadi, Mehrnoosh Samaei, Abdallah M Samy, Juan Sanabria, Milena M Santric-Milicevic, Muhammad Arif Nadeem Saqib, Arash Sarveazad, Brijesh Sathian, Maheswar Satpathy, Ione Jayce Ceola Schneider, Sadaf G Sepanlou, Allen Seylani, Sayed Mohammad Shafiee, Saeed Shahabi, Elaheh Shaker, Javad Sharifi-Rad, Sara Sheikhbahaei, Jeevan K Shetty, Parnian Shobeiri, Sudeep K Siddappa Malleshappa, Diego Augusto Santos Silva, Guilherme Silva Julian, Achintya Dinesh Singh, Jasvinder A Singh, Md Shahjahan Siraj, Valentin Yurievich Skryabin, Anna Aleksandrovna Skryabina, Bogdan Socea, Marco Solmi, Suhang Song, Miklós Szócska, Rafael Tabarés-Seisdedos, Elnaz Tabibian, Yasaman TaheriAbkenar, Iman M Talaat, Ker-Kan Tan, Abdelghani Tbakhi, Bekele Tesfaye, Amir Tiyuri, Daniel Nigusse Tollosa, Mathilde Touvier, Bach Xuan Tran, Biruk Shalmeno Tusa, Irfan Ullah, Saif Ullah, Marco Vacante, Sahel Valadan Tahbaz, Massimiliano Veroux, Bay Vo, Theo Vos, Ronny Westerman, Melat Woldemariam, Seyed Hossein Yahyazadeh Jabbari, Lin Yang, Fereshteh Yazdanpanah, Chuanhua Yu, Deniz Yuce, Ismaeel Yunusa, Vesna Zadnik, Mazyar Zahir, Zhi-Jiang Zhang, and Mohammad Zoladl.

Drafting the work or revising is critically for important intellectual content

Mohsen Abbasi-Kangevari, Rami Abd-Rabu, Hassan Abidi, Juan Manuel Acuna, Shailesh M Advani, Muhammad Sohail Afzal, Bright Opoku Ahinkorah, Ali Ahmadi, Sepideh Ahmadi, Luai A Ahmed, Muktar Beshir Ahmed, Fares Alahdab, Fadwa Alhalaiqa Naji Alhalaiqa, Motasem Alkhayyat, Sami Almustanyir, Saba Alvand, Nelson Alvis-Guzman, Saeed Amini, Robert Ancuceanu, Amir Anoushiravani, Ali Arash Anoushirvani, Jalal Arabloo, Floriane Ausloos, Marcel Ausloos, Atalel Fentahun Awedew, Tegegn Mulatu Ayana, Sina Azadnajafabad, Hiva Azami, Mohammadreza Azangou-Khyavy, Amirhossein Azari Jafari, Ashish D Badiye, Atif Amin Baig, Jennifer L Baker, Maciej Banach, Amadou Barrow, Sima Besharat, Devidas S Bhagat, Neeraj Bhala, Krittika Bhattacharyya, Vijayalakshmi S Bhojaraja, Sadia Bibi, Antonio Biondi, Tone Bjørge, Belay Boda Abule Bodicha, Hermann Brenner, Daniela Calina, Chao Cao, Yin Cao, Felix Carvalho, Ester Cerin, William C S Cho, Dinh-Toi Chu, Joao

Conde, Vera Marisa Costa, Natália Cruz-Martins, Anna Danielewicz, Getu Debalkie Demissie, Rupak Desai, Deepak Dhamnetiya, Mostafa Dianatinasab, Daniel Diaz, Mojtaba Didehdar, Saeid Doaei, Linh Phuong Doan, Milad Dodangeh, Fatemeh Eghbalian, Iman El Sayed, Muhammed Elhadi, Tahir Eyayu, Rana Ezzeddini, Ali Fatehizadeh, Masood Fereidoonnezhad, Eduarda Fernandes, Getahun Fetensa, Irina Filip, Florian Fischer, Masoud Foroutan, Peter Andras Gaal, Mohamed M Gad, Silvano Gallus, Tushar Garg, Tamiru Getachew, Seyyed-Hadi Ghamari, Ahmad Ghashghaee, Nermin Ghith, Jamshid Gholizadeh Navashenaq, James C Glasbey, Giuseppe Gorini, Avirup Guha, Sapna Gupta, Veer Bala Gupta, Vivek Kumar Gupta, Nima Hafezi-Nejad, Rabih Halwani, Shafiul Haque, Ahmed I Hasaballah, Simon I Hay, Claudiu Herteliu, Ramesh Holla, Mohammad-Salar Hosseini, Junjie Huang, Ayesha Humayun, Ivo Iavicoli, Olayinka Stephen Ilesanmi, Irena M Ilic, Milena D Ilic, Farhad Islami, Mohammad Ali Jahani, Mihajlo Jakovljevic, Rime Jebai, Ravi Prakash Jha, Nitin Joseph, Jacek Jerzy Jozwiak, Ali Kabir, Ashwin Kamath, Neeti Kapoor, Joonas H Kauppila, Asma Kazemi, Yousef Saleh Khader, Himanshu Khajuria, Rovshan Khalilov, Javad Khanali, Mahmoud Khodadost, Adnan Kisa, Sezer Kisa, Ali-Asghar Kolahi, Ai Koyanagi, Yuvaraj Krishnamoorthy, Manoj Kumar, Vivek Kumar, Carlo La Vecchia, Iván Landires, Yeong Yeh Lee, Elvynna Leong, Joana A Loureiro, Raimundas Lunevicius, Ata Mahmoodpoor, Mohammad-Reza Malekpour, Reza Malekzadeh, Ahmad Azam Malik, Lorenzo Giovanni Mantovani, Miquel Martorell, Jitendra Kumar Meena, Walter Mendoza, Alexios-Fotios A Mentis, Tomislav Mestrovic, Tomasz Miazgowski, Seyyedmohammadsadeq Mirmoeeni, Mohammad Mirza-Aghazadeh-Attari, Shafiu Mohammed, Teroj Abdulrahman Mohammed, Nagabhishek Moka, Ali H Mokdad, Zeinab Mokhtari, Sara Momtazmanesh, Lorenzo Monasta, Paula Moraga, Joana Morgado-da-Costa, Francesk Mulita, Mohsen Naghavi, Mukhammad David Naimzada, Hae Sung Nam, Zuhair S Natto, Biswa Prakash Nayak, Javad Nazari, Ehsan Nazemalhosseini-Mojarad, Ionut Negoi, Cuong Tat Nguyen, Son Hoang Nguyen, Nurulamin M Noor, Seyyed Mohammad Ali Noori, Virginia Nuñez-Samudio, Bogdan Oancea, Oluwakemi Ololade Odukoya, Ayodipupo Sikiru Oguntade, Andrew T Olagunju, Tinuke O Olagunju, Alicia Padron-Monedero, Reza Pakzad, Adrian Pana, Fatemeh Pashazadeh Kan, Urvish K Patel, Uttam Paudel, Renato B Pereira, Navaraj Perumalsamy, Richard G Pestell, Zahra Zahid Piracha, Richard Charles G Pollok, Akila Prashant, Mohammad Rabiee, Navid Rabiee, Amir Radfar, Vahid Rahmanian, Nazanin Rajai, Amirfarzan Rashidi, David Laith Rawaf, Salman Rawaf, Andre M N Renzaho, Nima Rezaei, Saeid Rezaei, Sahba Rezazadeh-Khadem, Gholamreza Roshandel, Maha Mohamed Saber-Ayad, Basema Saddik, Hossein Sadeghi, Umar Saeed, Amirhossein Sahebkar, Amir Salek Farrokhi, Hamideh Salimzadeh, Pouria Samadi, Mehrnoosh Samaei, Abdallah M Samy, Juan Sanabria, Milena M Santric-Milicevic, Maheswar Satpathy, Ione Jayce Ceola Schneider, Mario Šekerija, Sadaf G Sepanlou, Allen Seylani, Sayed Mohammad Shafiee, Zahra Shaghaghi, Maedeh Sharifian, Javad Sharifi-Rad, Sara Sheikhbahaei, Jeevan K Shetty, Reza Shirkoohi, Parnian Shobeiri, Sudeep K Siddappa Malleshappa, Diego Augusto Santos Silva, Guilherme Silva Julian, Achintya Dinesh Singh, Jasvinder A Singh, Gholam Reza Sivandzadeh, Bogdan Socea, Marco Solmi, Suhang Song, Majid Taheri, Yasaman TaheriAbkenar, Iman M Talaat, Ker-Kan Tan, Mathilde Touvier, Bach Xuan Tran, Biruk Shalmeno Tusa, Irfan Ullah, Saif Ullah, Marco Vacante, Sahel Valadan Tahbaz, Massimiliano Veroux, Cong Wang, Ronny Westerman, Seyed Hossein Yahyazadeh Jabbari, Lin Yang, Fereshteh Yazdanpanah, Mazyar Zahir, Iman Zare, and Mohammad Zoladl.

Managing the overall research enterprise

Lalit Dandona, Simon I Hay, Ali H Mokdad, and Mohsen Naghavi.

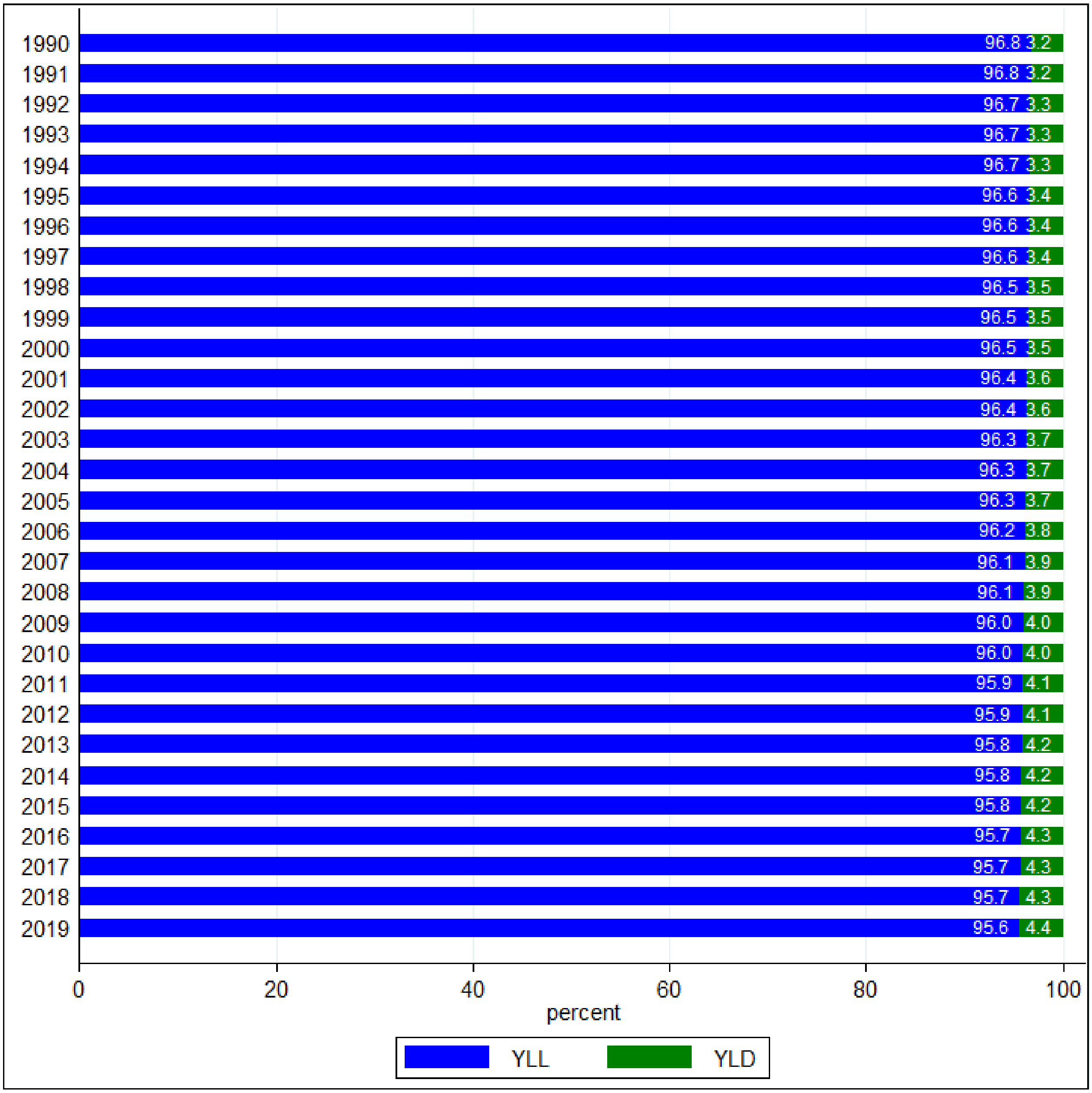

Supplementary Figure 1 Percent Contribution of YLL and YLD in All-age DALYs of Colorectal Cancer at Global Level, 1990-2019

YLL=Years of Life Lost; YLD=Years of Life Lived with Disability; DALYs=Disability-adjusted Life Years. DALYs are the sum of YLLs and YLDs. Data Source: Global Burden of Diseases, Injuries and Risk Factors Study 2019

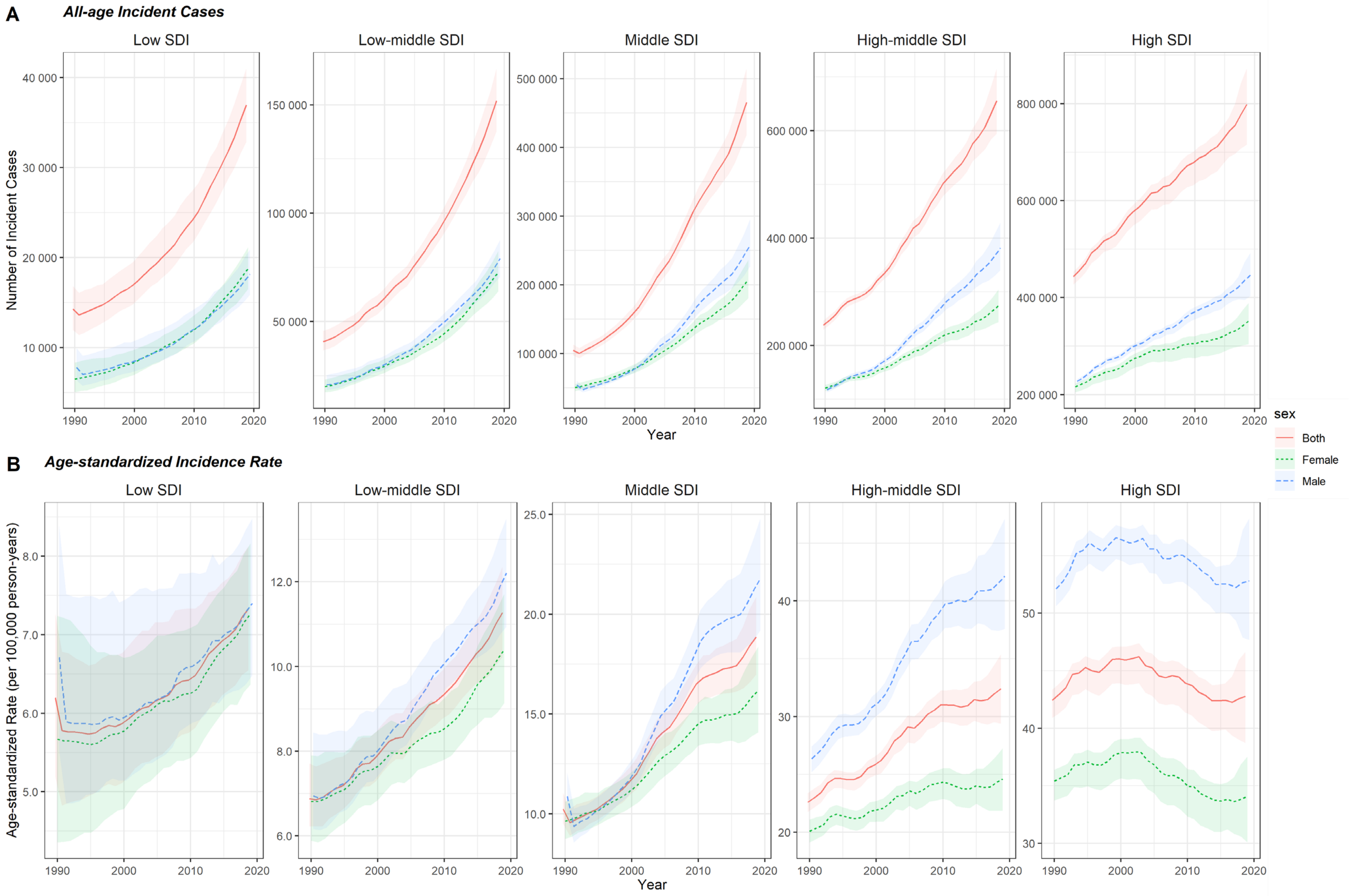

Supplementary Figure 2 Temporal Patterns of Colorectal Cancer Incidence by SDI, 1990-2019 A) All-age Incident Cases B) Age-standardized Incidence Rate (per 100,000 person-years).

SDI=Socio-demographic Index. Data Source: Global Burden of Diseases, Injuries and Risk Factors Study 2019

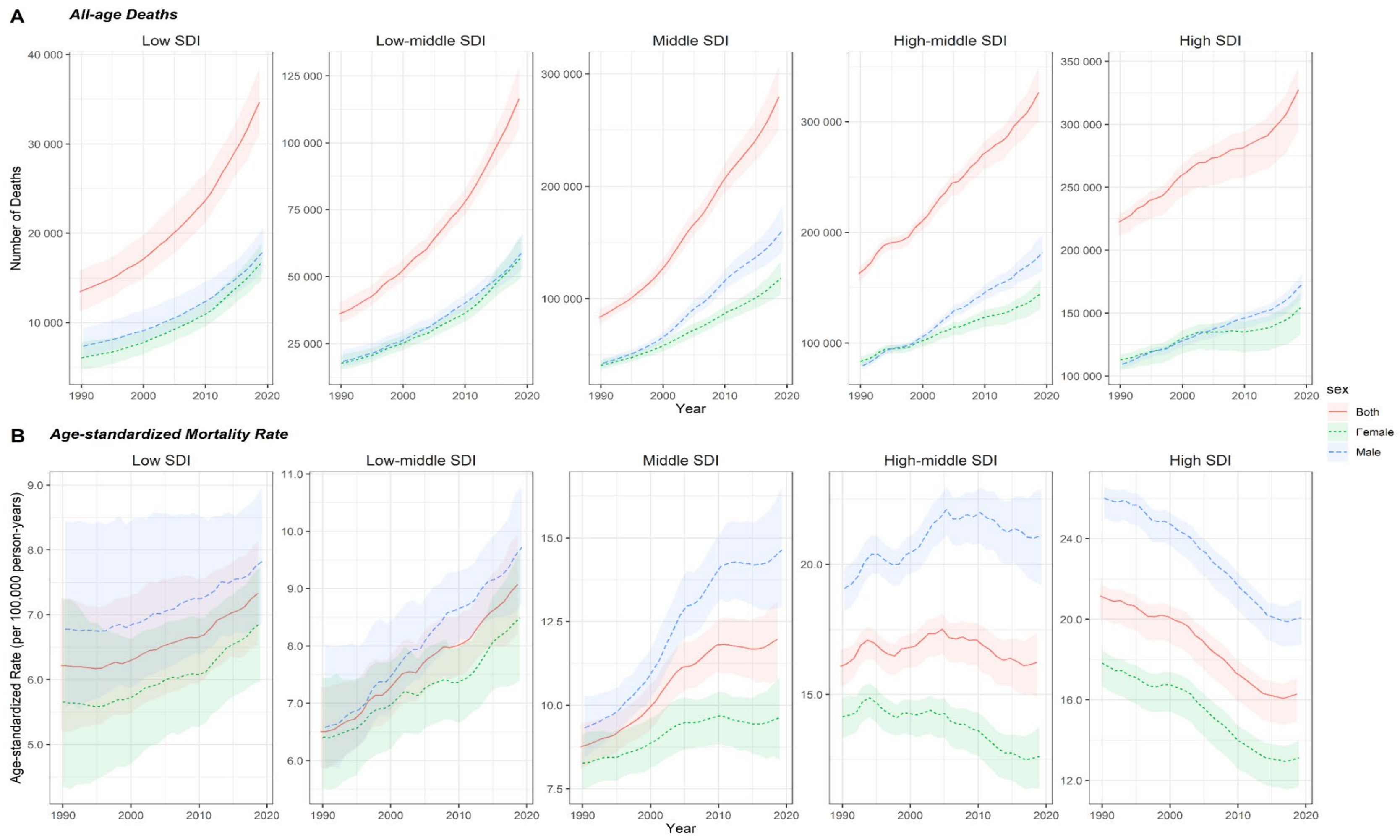

Supplementary Figure 3 Temporal Patterns of Colorectal Cancer Deaths by SDI, 1990-2019 A) All-age Deaths B) Age-standardized Mortality Rate (per 100,000 person-years).

SDI=Socio-demographic Index. Data Source: Global Burden of Diseases, Injuries and Risk Factors Study 2019

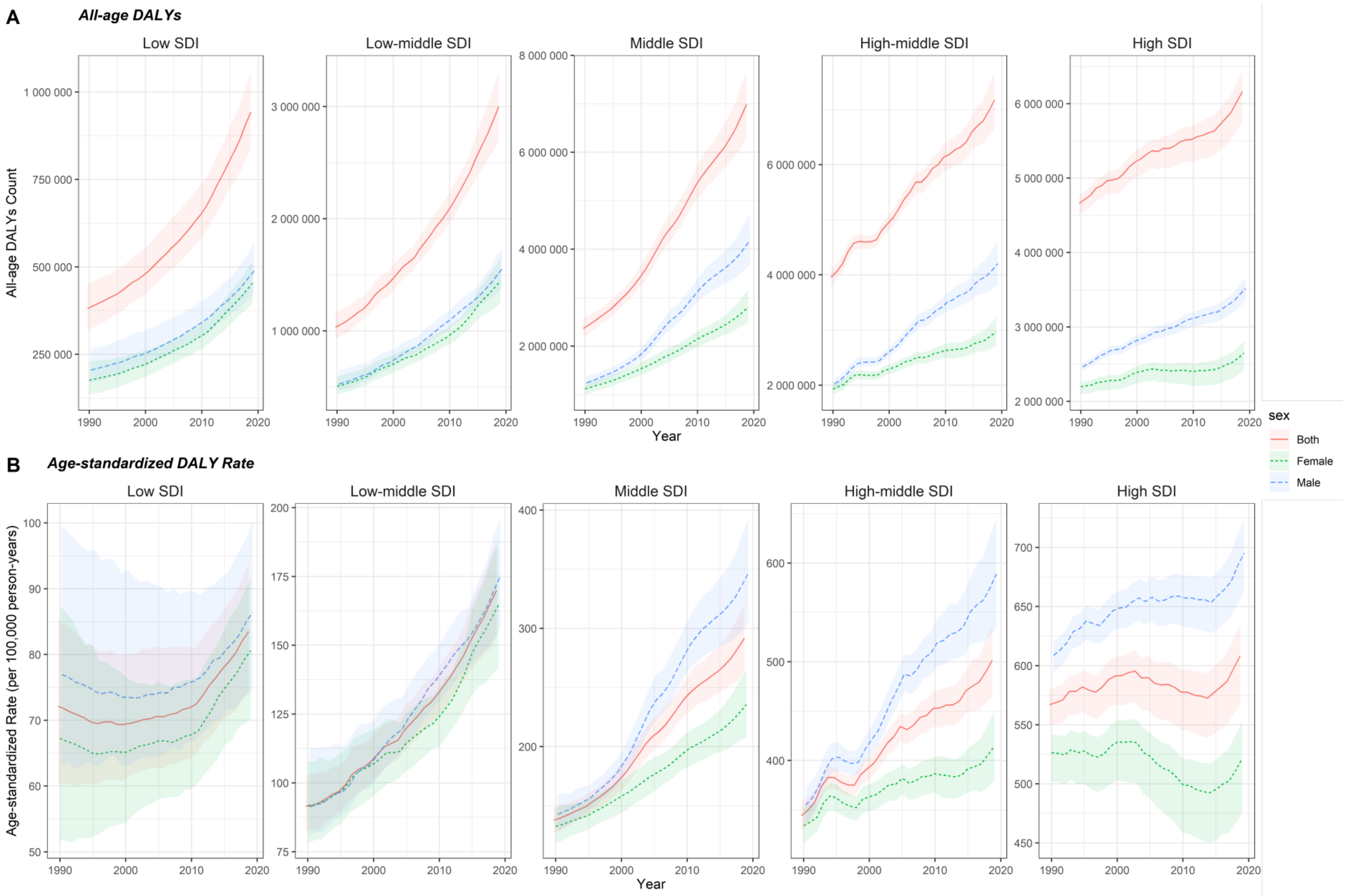

Supplementary Figure 4 Temporal Patterns of Colorectal Cancer DALYs by SDI, 1990-2019 A) All-age DALYs B) Age-standardized DALY Rate (per 100,000 person-years).

DALYs=Disability-adjusted Life Years. DALYs are sum of years of life lost and years lived with disability. SDI: Socio-demographic Index. Data Source: Global Burden of Diseases, Injuries and Risk Factors Study 2019

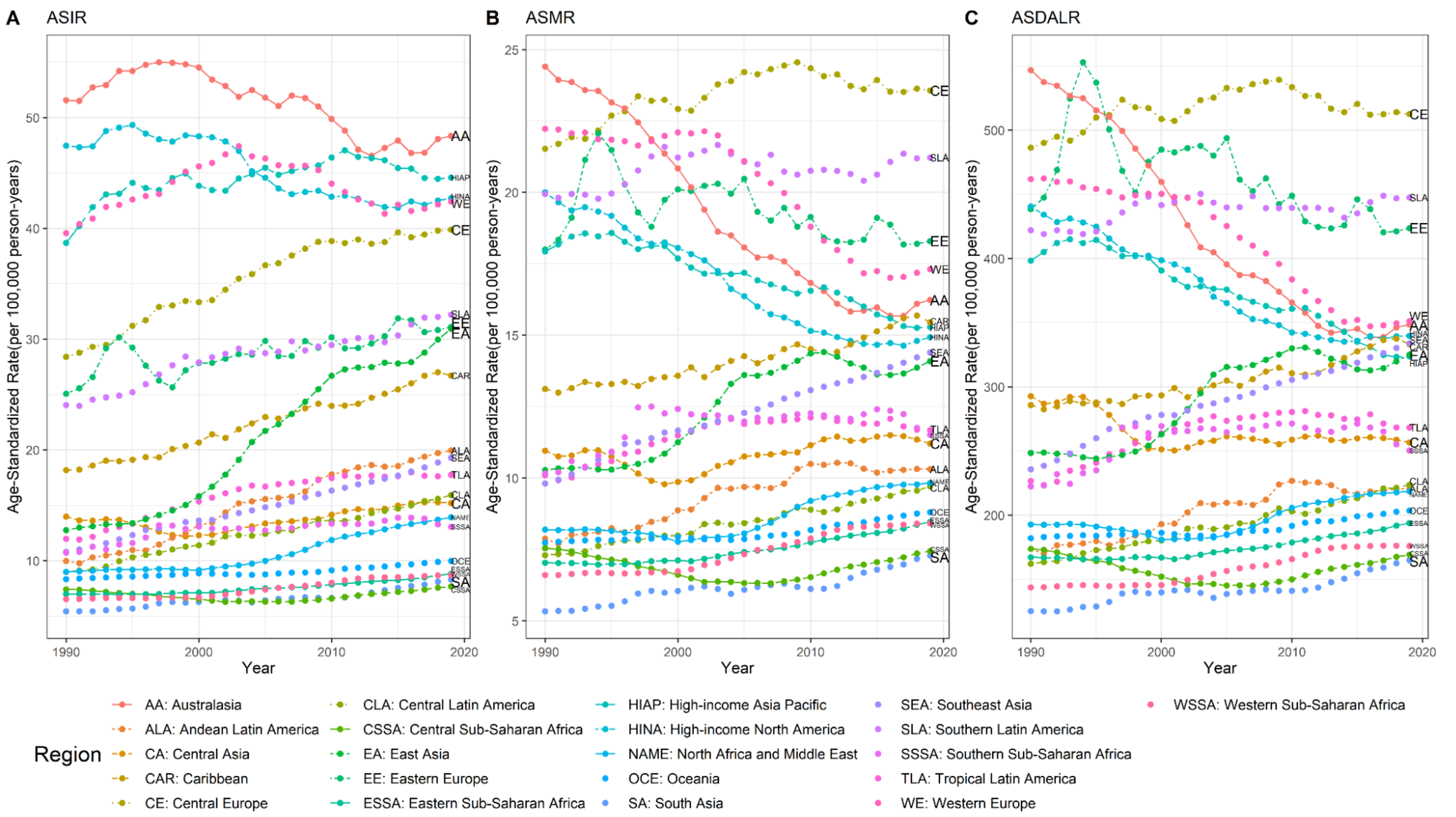

Supplementary Figure 5 Temporal Patterns of Colorectal Cancer Burden by Region, 1990-2019 A) ASIR B) ASMR C) ASDALR
ASIR=Age-standardized Incidence rate (per 100,000 person-years). ASMR=Age-standardized Mortality rate (per 100,000 person-years). ASDALR=Age-standardized DALY rate (per 100,000 person-years). DALYs are the sum of years of life lost and years lived with disability. Data Source: Global Burden of Diseases, Injuries and Risk Factors Study 2019

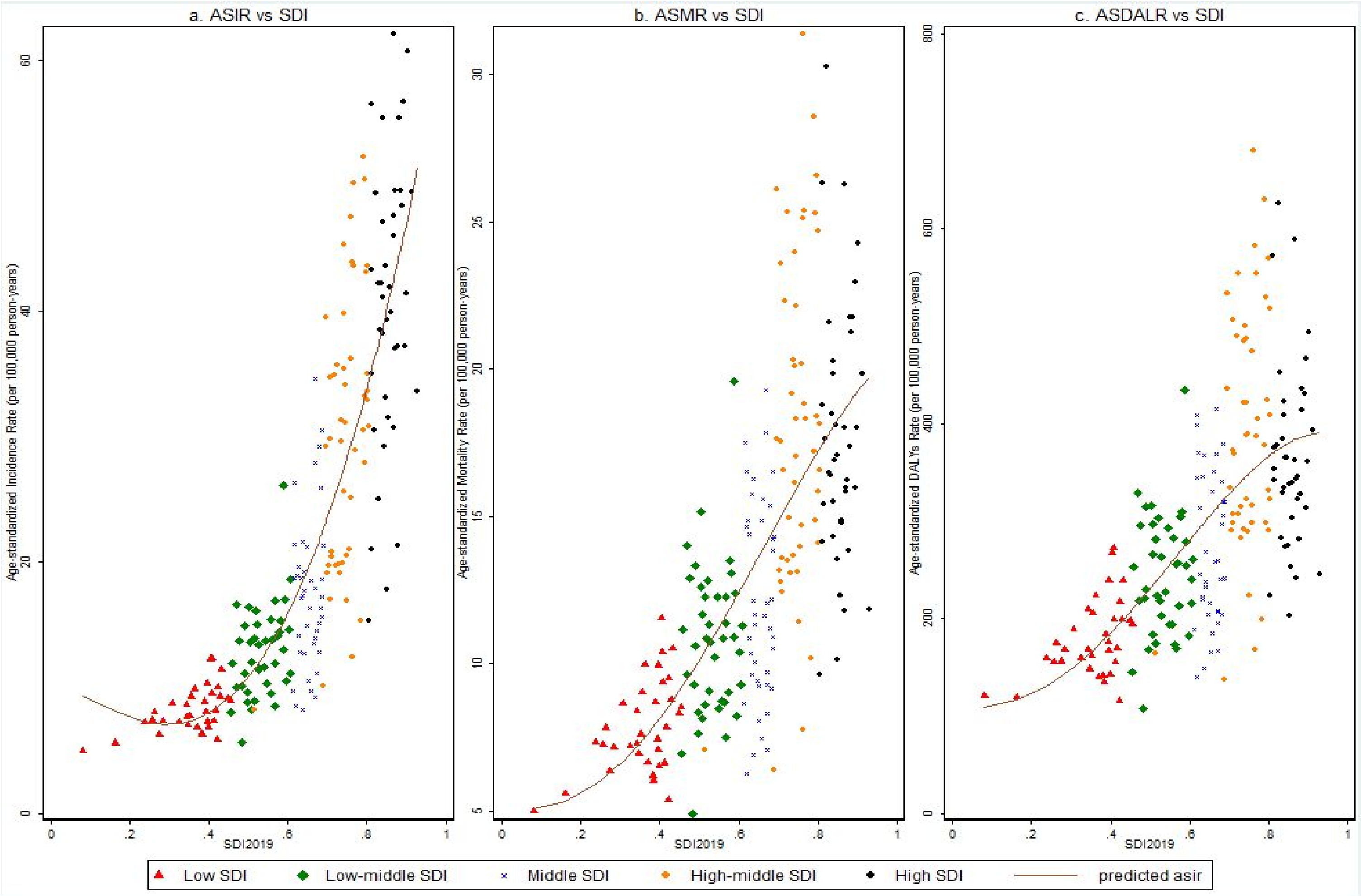

Supplementary Figure 6 Bivariate Association between Age-standardized Rates of Colorectal Cancer and Country-level SDI in 2019 a) ASIR vs SDI b) ASMR vs SDI c) ASDALR vs SDI.

ASIR=Age-standardized Incidence rate (per 100,000 person-years). ASMR=Age-standardized Mortality rate (per 100,000 person-years). ASDALR=Age-standardized DALY rate (per 100,000 person-years). SDI=Socio-demographic Index. The fitting line pertains to fractional polynomial regression. Data Source: Global Burden of Diseases, Injuries and Risk Factors Study 2019

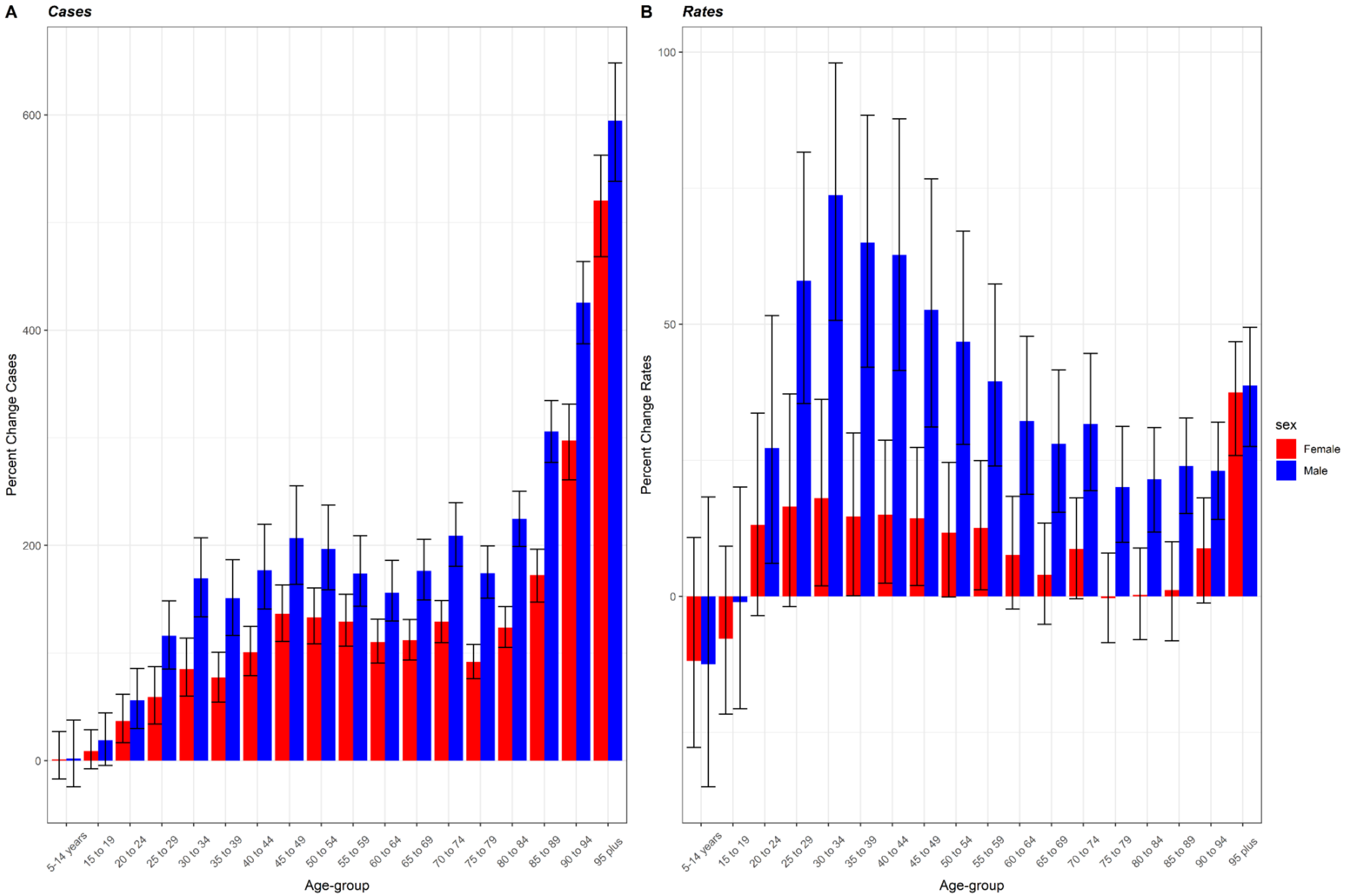

Supplementary Figure 7 Percent Changes in Global Burden of Colorectal Cancer Incidence between 1990 and 2019 by Age and Sex A) Incident Cases B) Age-specific Rate.

Error bars denote 95% uncertainty intervals. Data Source: Global Burden of Diseases, Injuries and Risk Factors Study 2019

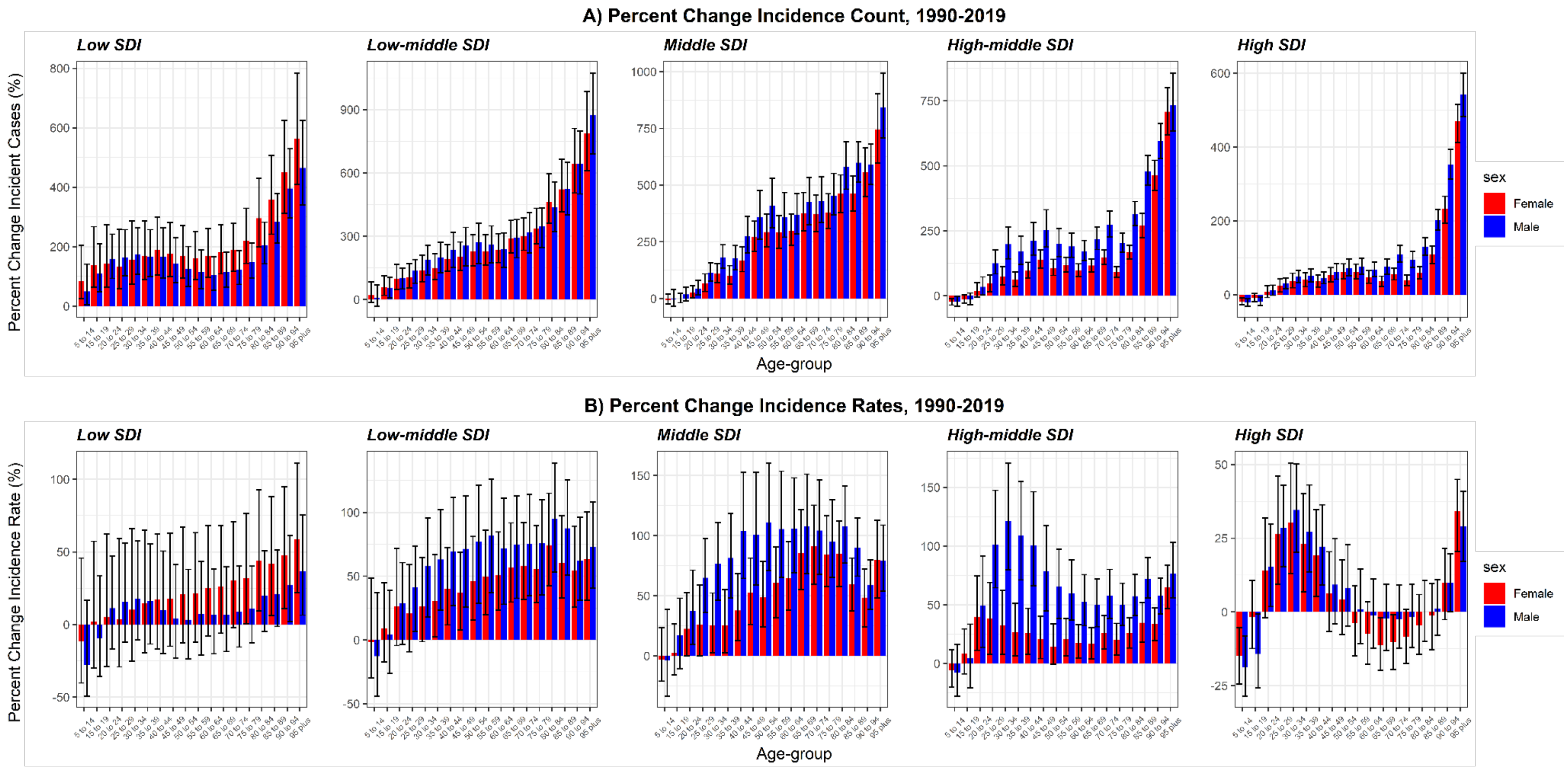

Supplementary Figure 8 SDI wise Percent Changes in Colorectal Cancer Incidence between 1990 and 2019 by Age and Sex A) Incident Cases B) Age-specific Incidence Rate.

Error bars denote 95% uncertainty intervals. Data Source: Global Burden of Diseases, Injuries and Risk Factors Study 2019

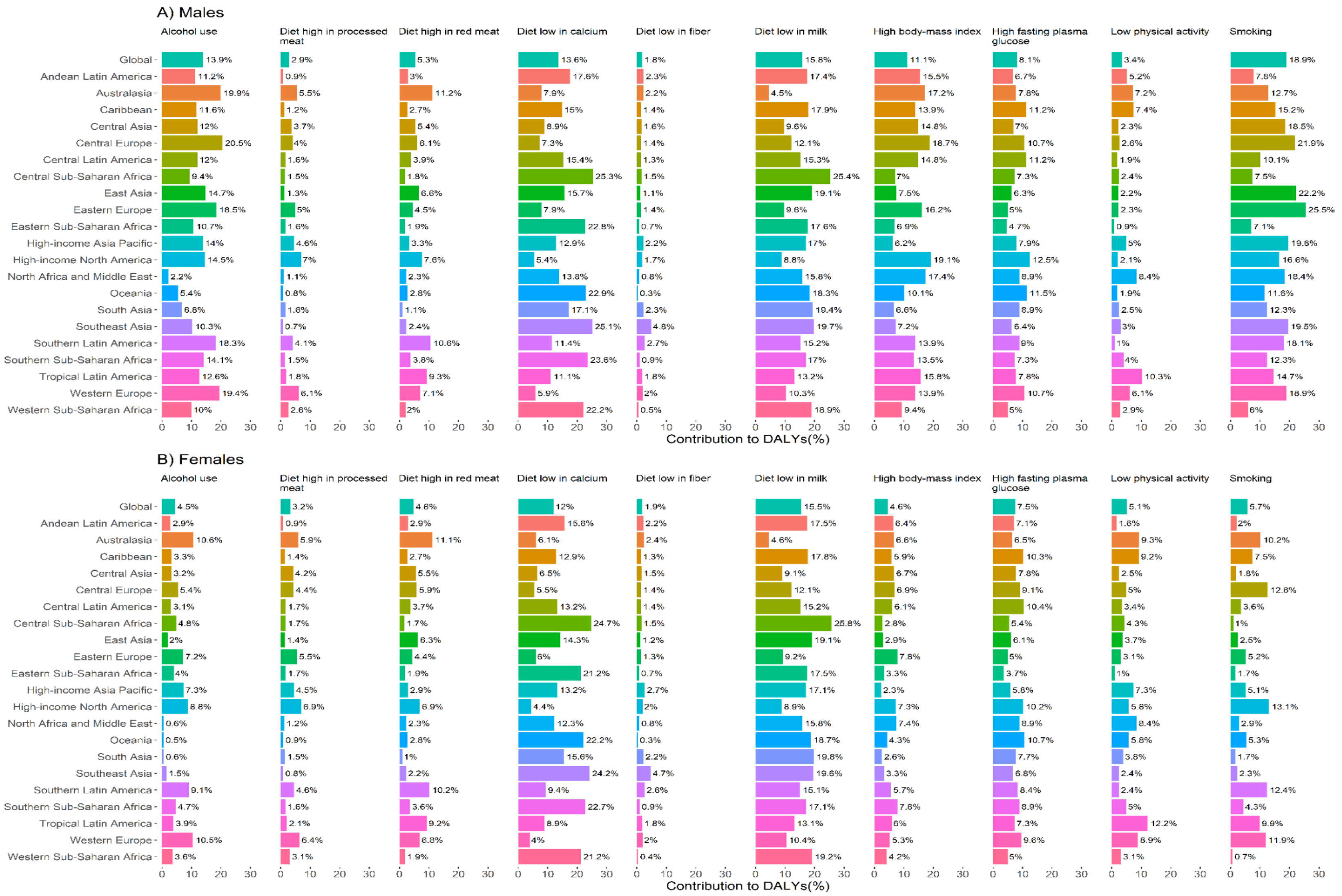

Supplementary Figure 9 Percent Contribution of Risk Factors in Colorectal Cancer DALYs in 2019 A) Males B) Females

DALYs=disability-adjusted life-years. Data Source: Global Burden of Diseases, Injuries and Risk Factors Study 2019

Supplementary Table 1 List of International Classification of Diseases (ICD) codes mapped to the Global Burden of Disease cause list for causes of death for Colorectal Cancer

ICCC3	ICD 10	ICD 9
XIf2, XIf3	C18, C18.0, C18.1, C18.2, C18.3, C18.4, C18.5, C18.6, C18.7, C18.8, C18.9, C19, C19.0, C19.9, C2, C20, C20.0, C20.8, C20.9, C21, C21.0, C21.1, C21.2, C21.8, C21.9	153, 153.0, 153.1, 153.2, 153.3, 153.4, 153.5, 153.6, 153.7, 153.8, 153.9, 154, 154.0, 154.1, 154.2, 154.3, 154.4, 154.8, 154.9, 209.1, 209.10, 209.11, 209.12, 209.13, 209.14, 209.15, 209.16, 209.17, 569.0, 569.43, 569.44, 569.84, 569.85

Supplementary Table 2 Covariates Used in Colorectal Cancer Mortality Estimation in CODEm.

	Males		Females	
**Level**	**Covariate**	**Direction**	**Covariate**	**Direction**
1	Age- and sex-specific summary exposure value (SEV) for High red meat	+	Age- and sex-specific summary exposure value (SEV) for High red meat	+
	Log-transformed SEV scalar: Colorectal Cancer	+	Log-transformed SEV scalar: Colorectal Cancer	+
	Mean Body Mass Index	+	Mean Body Mass Index	+
	Tobacco (Cigarettes per capita)	+	Tobacco (cigarettes per capita)	+
	Total Physical Activity (MET-min/week), Age-specific	-	Total Physical Activity (MET-min/week), Age-specific	-
2	Age- and sex- specific SEV for Low vegetables	+	Age- and sex- specific SEV for Low vegetables	+
	Age- and sex-specific SEV for Low calcium	+	Age- and sex-specific SEV for Low calcium	+
	Age- and sex-specific SEV for Low fiber	+	Age- and sex-specific SEV for Low fiber	+
	Cumulative Cigarettes (20 years)	+	Cumulative cigarettes (5 years)	+

	Males		Females	
**Level**	**Covariate**	**Direction**	**Covariate**	**Direction**
	Diabetes Fasting Plasma Glucose (mmol/L), age-standardized 25+	+	Diabetes Fasting Plasma Glucose (mmol/L), age-standardized 25+	+
	Liters of alcohol consumed per capita	+	Liters of alcohol consumed per capita	+
	Polyunsaturated Fatty Acid adjusted (Percent)	-	Polyunsaturated Fatty Acid adjusted (Percent)	-
	Age- and sex- specific SEV for low fruit	+	Age- and sex- specific SEV for low fruit	+
	Age- and sex-specific SEV for Low milk	+	Age- and sex-specific SEV for Low milk	+
	Age- and sex-specific SEV for Low nuts and seeds	+	Age- and sex-specific SEV for Low nuts and seeds	+
	Education (years per capita)	-	Education (years per capita)	-
	Healthcare Access and Quality Index	-	Healthcare Access and Quality Index	-
	Lag distributed income (I$ per capita)	+	Lag distributed income (I$ per capita)	+
3	Socio-demographic Index	+	Socio-demographic Index	+

MET=Metabolic equivalent of task. The "Direction" column refers to the direction of the association for that covariate.

Supplementary Table 3 Number of data sources used as inputs for calculation of exposure of ten risk factors.

Risk Factor	Number of data sources(site-years)	Countries with data
Alcohol Use	10513	199
Processed Meat	737	66
Red meat	760	178
Calcium	160	178
Fiber	155	180
Milk	1148	177
BMI	2022	190
FPG	529	127
Low physical Activity	255	128
Smoking	3439	201

Source: Global Burden of Diseases, Injuries and Risk Factors Study 2019.

Supplementary Table 3 Country-wise Colorectal Cancer Burden in 2019

Location	Incidence - number of cases (95% UI)	Mortality - number of deaths (95% UI)	DALYs (95% UI)	Age-standardised incidence rate (95% UI)	Age-standardised Mortality rate (95% UI)	Age-standardised DALY rate (95% UI)
**Andean Latin America**	**11094 (8935 - 13467)**	**5630 (4593 - 6791)**	**125578 (101753 - 151796)**	**20.0 (16.1 - 24.2)**	**10.3 (8.4 - 12.4)**	**220.8 (179.0 - 266.3)**
Bolivia	1439 (991 - 1911)	992 (678 - 1314)	23074 (16068 - 30578)	16.9 (11.6 - 22.4)	12.3 (8.4 - 16.2)	256.2 (178.2 - 339.7)
Ecuador	2850 (2269 - 3613)	1603 (1285 - 2008)	36473 (28821 - 46627)	19.3 (15.3 - 24.3)	11.3 (9.1 - 14.2)	237.6 (188.9 – 301.0)
Peru	6805 (5101 - 8908)	3035 (2291 - 3931)	66030 (49368 - 86743)	21.2 (15.9 - 27.8)	9.4 (7.1 - 12.3)	203.9 (152.4 - 267.9)
**Australasia**	**23671 (19439 - 28848)**	**8382 (7575 - 8978)**	**163248 (150872 - 173959)**	**48.3 (39.6 - 59.1)**	**16.2 (14.8 - 17.3)**	**348.6 (324.2 - 370.7)**
Australia	19345 (15224 - 24451)	6734 (6065 - 7265)	131642 (120904 - 141505)	47.0 (36.9 - 59.6)	15.5 (14.1 - 16.7)	334.6 (309.2 - 359.1)
New Zealand	4326 (3591 - 5137)	1648 (1481 - 1780)	31606 (29269 - 33829)	55.3 (45.7 - 65.8)	20.3 (18.3 - 21.8)	423.1 (394.6 - 449.8)
**Caribbean**	**13813 (11813 - 15959)**	**7995 (6935 - 9176)**	**172016 (147186 - 200175)**	**26.7 (22.9 - 30.9)**	**15.5 (13.4 - 17.7)**	**333.3 (285.2 - 387.9)**

Location	Incidence - number of cases (95% UI)	Mortality - number of deaths (95% UI)	DALYs (95% UI)	Age-standardised incidence rate (95% UI)	Age-standardised Mortality rate (95% UI)	Age-standardised DALY rate (95% UI)
Antigua and Barbuda	25<br>(21 - 29)	15<br>(13 - 17)	327<br>(278 - 382)	25.6<br>(22.1 - 29.4)	16.2<br>(14.0 - 18.4)	323.0<br>(276.8 - 374.9)
Bahamas	108<br>(88 - 131)	68<br>(55 - 82)	1709<br>(1390 - 2098)	28.0<br>(22.8 – 34.0)	18.4<br>(15.1 - 22.2)	423.6<br>(346.4 - 515.5)
Barbados	195<br>(162 - 233)	118<br>(99 - 139)	2392<br>(1977 - 2865)	39.8<br>(33.0 - 47.4)	24.0<br>(20.1 - 28.3)	500.6<br>(413.4 - 600.9)
Belize	41<br>(35 - 47)	28<br>(24 - 32)	716<br>(622 - 829)	14.6<br>(12.8 – 17.0)	10.4<br>(9.0 – 12.0)	239.7<br>(209.1 - 276.4)
Bermuda	56<br>(47 - 68)	26<br>(21 - 31)	469<br>(390 - 571)	43.3<br>(36.1 - 52.8)	18.8<br>(15.7 - 22.7)	375.6<br>(311.2 - 456.8)
Cuba	6569<br>(5388 - 7975)	3481<br>(2883 - 4186)	68409<br>(56038 - 83589)	34.6<br>(28.2 – 42.0)	17.8<br>(14.7 - 21.5)	369.0<br>(300.5 - 452.2)
Dominica	18<br>(15 - 22)	14<br>(11 - 16)	277<br>(227 - 342)	19.8<br>(16.3 - 24.2)	15.0<br>(12.4 - 18.1)	314.4<br>(256.7 - 387.3)
Dominican Republic	1579<br>(1133 - 2067)	1115<br>(823 - 1448)	26797<br>(18887 - 35321)	17.1<br>(12.4 - 22.3)	12.4<br>(9.2 - 16.1)	279.2<br>(198.2 - 367.6)
Grenada	30<br>(27 - 33)	20<br>(18 - 22)	466<br>(419 - 517)	27.9<br>(25.1 - 30.7)	19.3<br>(17.4 - 21.1)	414.8<br>(374 - 457.8)

Location	Incidence - number of cases (95% UI)	Mortality - number of deaths (95% UI)	DALYs (95% UI)	Age-standardised incidence rate (95% UI)	Age-standardised Mortality rate (95% UI)	Age-standardised DALY rate (95% UI)
Guyana	116 (90 - 147)	85 (68 - 107)	2295 (1773 - 2936)	18.7 (14.8 - 23.6)	14.7 (11.9 - 18.2)	344.8 (269.2 - 437.9)
Haiti	779 (536 - 1065)	671 (467 - 917)	18317 (12572 - 25414)	11.5 (8.0 - 15.4)	10.5 (7.3 - 14.1)	239.8 (166.0 – 329.0)
Jamaica	772 (622 - 960)	501 (405 - 615)	11263 (8962 - 14208)	26.0 (20.8 - 32.3)	16.5 (13.4 - 20.4)	378.9 (301.5 - 477.6)
Puerto Rico	2379 (1864 - 3026)	1136 (892 - 1429)	22451 (17568 - 28605)	35.0 (27.0 - 44.8)	15.4 (12.2 - 19.5)	353.9 (274.8 - 454.9)
Saint Kitts and Nevis	20 (16 - 23)	11 (9 - 13)	263 (209 - 315)	31.1 (26.3 - 36.4)	18.3 (15.8 - 21.1)	389.5 (318.9 - 463.1)
Saint Lucia	38 (32 - 45)	25 (21 - 29)	558 (467 - 662)	18.0 (15.1 - 21.2)	12.1 (10.2 - 14.1)	259.5 (218.1 - 307.9)
Saint Vincent and the Grenadines	26 (23 - 30)	19 (16 - 21)	422 (367 - 487)	19.7 (17.2 - 22.6)	14.4 (12.7 - 16.5)	313.8 (274.1 - 360.9)
Suriname	128 (105 - 152)	94 (78 - 111)	2236 (1839 - 2690)	21.6 (17.9 - 25.6)	16.3 (13.6 - 19.2)	367.4 (302.8 - 440.1)
Trinidad and Tobago	387 (296 - 498)	253 (194 - 326)	5802 (4361 - 7586)	21.1 (16.1 - 27.1)	14.0 (10.7 – 18.0)	315.4 (237.5 – 412.0)

Location	Incidence - number of cases (95% UI)	Mortality - number of deaths (95% UI)	DALYs (95% UI)	Age-standardise d incidence rate (95% UI)	Age-standardised Mortality rate (95% UI)	Age-standardised DALY rate (95% UI)
United States Virgin Islands	79 (66 - 92)	48 (40 - 55)	1018 (832 - 1203)	43.0 (35.7 - 50.6)	26.6 (22.3 – 31.0)	569.1 (463.8 – 678.0)
**Central Asia**	**10949 (9999 - 12008)**	**7467 (6822 - 8166)**	**199841 (182012 - 219941)**	**15.2 (13.9 - 16.6)**	**11.2 (10.3 - 12.2)**	**256.8 (234.4 - 281.1)**
Armenia	880 (738 - 1035)	585 (492 - 681)	13091 (10974 - 15396)	21.4 (17.8 – 25.0)	14.3 (12.1 - 16.6)	319.7 (267.4 - 375.2)
Azerbaijan	1558 (1280 - 1911)	1063 (875 - 1304)	30658 (25244 - 37396)	16.3 (13.3 - 20.1)	12.2 (9.8 - 15.2)	296.9 (244.9 - 362.7)
Georgia	1096 (920 - 1284)	782 (659 - 913)	18328 (15328 - 21549)	19.1 (16.0 - 22.5)	13.2 (11.1 - 15.4)	333.8 (279.7 - 393.7)
Kazakhstan	3378 (2927 - 3878)	2190 (1906 - 2491)	54853 (47240 - 63021)	19.7 (17.2 - 22.5)	13.5 (11.8 - 15.3)	307.7 (267.0 - 351.7)
Kyrgyzstan	472 (412 - 541)	346 (304 - 394)	8817 (7649 - 10118)	10.5 (9.2 – 12.0)	8.2 (7.2 - 9.3)	182.5 (158.8 - 208)
Mongolia	256 (199 - 333)	193 (150 - 248)	5658 (4366 - 7438)	11.1 (8.8 - 14.1)	9.3 (7.4 - 11.7)	215.5 (169.2 - 277.8)
Tajikistan	551 (443 - 674)	428 (347 - 526)	12797 (10254 - 15802)	11.6 (9.5 - 14.1)	10.2 (8.4 - 12.3)	227.6 (184.5 - 277.3)

Location	Incidence - number of cases (95% UI)	Mortality - number of deaths (95% UI)	DALYs (95% UI)	Age-standardised incidence rate (95% UI)	Age-standardised Mortality rate (95% UI)	Age-standardised DALY rate (95% UI)
Turkmenistan	354 (284 - 439)	254 (207 - 314)	6955 (5526 - 8711)	9.2 (7.5 - 11.3)	7.1 (5.8 - 8.7)	166.6 (134.3 - 206.9)
Uzbekistan	2404 (2026 - 2826)	1626 (1381 - 1901)	48683 (41086 - 57806)	12.8 (11.1 - 14.7)	10.4 (9.1 - 11.8)	219.6 (188.3 - 253.4)
**Central Europe**	**84474 (74551 - 95453)**	**51567 (45636 - 57749)**	**1052146 (922923 - 1184246)**	**39.9 (35.2 - 45.1)**	**23.6 (20.8 - 26.4)**	**512.6 (448.7 - 577.9)**
Albania	630 (470 - 828)	385 (291 - 502)	8225 (6122 - 10808)	15.2 (11.4 - 19.9)	9.1 (7.0 - 11.9)	204.5 (153.1 - 268.2)
Bosnia and Herzegovina	2058 (1619 - 2571)	1319 (1044 - 1639)	28249 (22241 - 35385)	34.9 (27.5 - 43.5)	22.3 (17.8 - 27.7)	490.1 (386.1 - 613.8)
Bulgaria	6199 (4975 - 7641)	3672 (2991 - 4477)	78043 (62257 - 96600)	43.8 (35.0 - 54.2)	25.1 (20.4 - 30.6)	582.3 (462.4 - 724.9)
Croatia	4409 (3503 - 5472)	2308 (1843 - 2847)	44362 (35211 - 55455)	50.5 (39.9 - 62.6)	25.3 (20.1 - 31.2)	529.2 (415.5 - 665.8)
Czechia	8839 (7288 - 10730)	4678 (3864 - 5626)	91540 (75040 - 111253)	42.1 (34.5 - 51.1)	21.6 (17.8 – 26.0)	452.1 (370.1 - 551.2)
Hungary	9873 (8137 - 11905)	5646 (4699 - 6700)	115101 (94609 - 138287)	52.2 (43.0 - 62.9)	28.6 (23.6 – 34.0)	630.3 (519.2 - 763.5)

Location	Incidence - number of cases (95% UI)	Mortality - number of deaths (95% UI)	DALYs (95% UI)	Age-standardised incidence rate (95% UI)	Age-standardised Mortality rate (95% UI)	Age-standardised DALY rate (95% UI)
Montenegro	299 (245 - 362)	168 (137 - 200)	3663 (3014 - 4386)	30.5 (25.0 - 36.8)	17.2 (14.1 - 20.4)	377.3 (309.5 - 451.7)
North Macedonia	1135 (898 - 1416)	683 (545 - 850)	15695 (12333 - 19727)	35.4 (28.2 - 43.9)	22.1 (17.8 - 27.3)	487.5 (386.3 - 610.7)
Poland	24277 (20748 - 28769)	17768 (15129 - 20758)	350819 (296650 - 412619)	35.0 (29.8 - 41.6)	24.7 (21.0 – 29.0)	518.0 (437.3 - 610.6)
Romania	13039 (10746 - 15541)	7577 (6281 - 9022)	163169 (133789 - 196693)	36.2 (29.5 - 43.3)	20.2 (16.6 - 24.1)	474.4 (387.3 - 575.8)
Serbia	6789 (5452 - 8446)	4018 (3269 - 4943)	84596 (67490 - 105391)	43.6 (35.1 - 54.3)	25.4 (20.6 – 31.0)	554.8 (441.7 - 693.8)
Slovakia	5203 (4087 - 6574)	2433 (1932 - 3038)	51875 (40791 - 65885)	56.4 (44.4 – 71.0)	26.3 (21.0 - 32.8)	571.6 (449.1 - 723.9)
Slovenia	1726 (1347 - 2222)	913 (720 - 1163)	16809 (13088 - 21737)	41.1 (31.9 - 52.9)	19.8 (15.6 - 25.4)	408.2 (317.5 - 532.1)
**Central Latin America**	**37542 (32211 - 43870)**	**22470 (19542 - 25997)**	**539638 (465200 - 627069)**	**15.9 (13.7 - 18.6)**	**9.7 (8.4 - 11.2)**	**223.7 (193.1 - 259.5)**
Colombia	9046 (7010 - 11553)	5138 (3990 - 6532)	116742 (90051 - 150159)	17.1 (13.3 - 21.9)	9.6 (7.5 - 12.2)	222.2 (171.3 - 285.6)

Location	Incidence - number of cases (95% UI)	Mortality - number of deaths (95% UI)	DALYs (95% UI)	Age-standardised incidence rate (95% UI)	Age-standardised Mortality rate (95% UI)	Age-standardised DALY rate (95% UI)
Costa Rica	1496 (1161 - 1902)	788 (622 - 986)	17593 (13679 - 22430)	29.2 (22.7 - 37.2)	15.4 (12.2 - 19.3)	340.4 (264.8 - 432.6)
El Salvador	844 (644 - 1096)	552 (426 - 710)	12658 (9679 - 16508)	14.1 (10.7 - 18.3)	9.1 (7.0 - 11.7)	213.5 (162.6 – 279.0)
Guatemala	1290 (1020 - 1619)	965 (771 - 1208)	24462 (19260 - 30978)	11.5 (9.2 - 14.4)	9.1 (7.3 - 11.3)	203.1 (160.2 – 256.0)
Honduras	574 (398 - 812)	430 (303 - 605)	10631 (7442 - 15137)	9.7 (6.7 - 13.6)	7.7 (5.3 - 10.7)	167.7 (117.1 - 239.1)
Mexico	17470 (15042 - 20060)	10518 (9036 - 12022)	260198 (223549 - 299468)	14.9 (12.9 - 17.1)	9.2 (8.0 - 10.5)	215.3 (185.1 - 247.2)
Nicaragua	689 (571 - 820)	430 (362 - 502)	10192 (8311 - 12295)	16.2 (13.4 – 19.0)	10.9 (9.2 - 12.5)	223.6 (184.7 - 266.1)
Panama	772 (599 - 979)	455 (357 - 576)	9974 (7720 - 12719)	18.6 (14.4 - 23.6)	10.9 (8.5 - 13.8)	239.9 (185.6 - 305.4)
Venezuela (Bolivarian Republic of)	5361 (4064 - 7020)	3194 (2439 - 4131)	77189 (57827 - 102452)	18.6 (14.1 - 24.2)	11.3 (8.7 - 14.6)	261.1 (197.5 - 344.8)
**Central Sub Saharan Africa**	**3957 (3015 - 5113)**	**3544 (2705 - 4609)**	**100988 (75749 - 131447)**	**7.7 (5.9 - 10.1)**	**7.4 (5.7 - 9.9)**	**169.3 (129.2 - 220.2)**

Location	Incidence - number of cases (95% UI)	Mortality - number of deaths (95% UI)	DALYs (95% UI)	Age-standardised incidence rate (95% UI)	Age-standardised Mortality rate (95% UI)	Age-standardised DALY rate (95% UI)
Angola	1083 (829 - 1389)	952 (738 - 1212)	27809 (20727 - 35928)	10.0 (8.1 - 12.5)	9.7 (7.8 – 12.0)	218.4 (170.0 - 277.4)
Central African Republic	138 (98 - 192)	128 (92 - 178)	4004 (2786 - 5605)	6.3 (4.6 - 8.7)	6.4 (4.7 - 8.8)	156.2 (112.9 - 216.3)
Congo	303 (219 - 405)	266 (197 - 352)	7545 (5344 - 10226)	11.9 (9.1 - 15.4)	11.4 (8.8 - 14.5)	257.2 (189.0 - 341.6)
Democratic Republic of the Congo	2194 (1480 - 3261)	1995 (1339 - 2956)	56267 (38083 - 83820)	6.4 (4.2 - 9.6)	6.2 (4.1 - 9.5)	141.4 (95.1 - 210.5)
Equatorial Guinea	73 (45 - 108)	61 (38 - 89)	1653 (1002 - 2514)	15.6 (9.8 - 22.4)	14.2 (9.1 – 20.0)	305.6 (189.9 - 449.5)
Gabon	166 (119 - 210)	141 (103 - 177)	3710 (2597 - 4832)	16.4 (12.1 - 20.3)	14.9 (11.3 - 18.2)	330.6 (237.6 - 418.6)
**Eastern Asia**	**637096 (548895 - 738549)**	**275604 (238238 - 317886)**	**6712862 (5774277 - 7735907)**	**30.9 (26.8 - 35.7)**	**14.1 (12.2 - 16.2)**	**325.2 (280.7 - 373.2)**
China	607900 (521805 - 708420)	261777 (224404 - 303318)	6394918 (5462291 - 7408703)	30.6 (26.4 - 35.5)	13.9 (11.9 – 16.0)	320.6 (275.4 - 370.7)
Democratic People's Republic	4986	3442	92302	15.4	10.9	282.7

Location	Incidence - number of cases (95% UI)	Mortality - number of deaths (95% UI)	DALYs (95% UI)	Age-standardised incidence rate (95% UI)	Age-standardised Mortality rate (95% UI)	Age-standardised DALY rate (95% UI)
of Korea	(3708 - 6464)	(2576 - 4380)	(67639 - 122531)	(11.5 - 19.9)	(8.1 - 13.8)	(207.2 - 376.7)
Taiwan (Province of China)	24210 (19139 - 31206)	10386 (8282 - 13153)	225642 (176874 - 291070)	62.0 (48.9 - 80.1)	26.3 (20.9 - 33.3)	588.4 (461.5 - 757.8)
**Eastern Europe**	**106017 (96250 - 117074)**	**63476 (57180 - 70011)**	**1419105 (1287540 - 1571374)**	**31.1 (28.2 - 34.4)**	**18.3 (16.5 - 20.2)**	**423.7 (384.0 - 469.3)**
Belarus	5396 (4322 - 6837)	2734 (2230 - 3445)	60384 (47970 - 78137)	34.1 (27.1 - 43.4)	17.0 (13.9 - 21.5)	388.7 (308.8 - 500.2)
Estonia	1118 (892 - 1391)	528 (425 - 660)	9665 (7667 - 12205)	42.2 (33.4 - 52.7)	18.5 (14.8 - 23.2)	384.2 (304.2 - 486.6)
Latvia	1214 (1015 - 1461)	746 (626 - 891)	14162 (11810 - 17165)	30.5 (25.4 – 37.0)	17.6 (14.8 - 21.2)	377.4 (314.6 - 460.4)
Lithuania	1669 (1385 - 2008)	1024 (857 - 1221)	19673 (16149 - 23901)	29.2 (24.0 - 35.4)	16.9 (14.1 - 20.3)	365.6 (299.1 - 446.1)
Republic of Moldova	1691 (1472 - 1927)	1028 (897 - 1164)	24957 (21689 - 28419)	29.2 (25.4 - 33.1)	17.7 (15.4 – 20.0)	436.3 (378.9 - 496.1)
Russian Federation	71542 (62884 - 81644)	42834 (37637 - 48395)	939798 (822798 - 1069392)	30.8 (27.0 - 35.1)	18.2 (16.0 - 20.5)	409.0 (357.7 – 466.0)

Location	Incidence - number of cases (95% UI)	Mortality - number of deaths (95% UI)	DALYs (95% UI)	Age-standardise d incidence rate (95% UI)	Age-standardised Mortality rate (95% UI)	Age-standardised DALY rate (95% UI)
Ukraine	23388 (19879 - 27253)	14582 (12548 - 16939)	350467 (300711 - 412417)	31.3 (26.6 - 36.5)	19.2 (16.5 - 22.4)	485.3 (416.3 - 572.3)
**Eastern Sub Saharan Africa**	**14227 (12130 - 16886)**	**12717 (10940 - 15001)**	**356433 (301931 - 425606)**	**8.8 (7.6 - 10.4)**	**8.5 (7.4 - 9.9)**	**193.9 (166.0 - 229.6)**
Burundi	335 (237 - 484)	303 (216 - 438)	8860 (6210 - 12874)	7.3 (5.3 - 10.4)	7.2 (5.2 - 10.1)	168.4 (119.7 - 243.2)
Comoros	43 (31 - 55)	39 (29 - 50)	996 (702 - 1311)	9.0 (6.6 - 11.4)	8.6 (6.4 - 10.8)	194.4 (139.5 - 252.2)
Djibouti	70 (49 - 97)	59 (43 - 82)	1739 (1181 - 2490)	11.9 (9.1 - 15.8)	11.2 (8.7 - 14.7)	253.1 (184.4 - 346.6)
Eritrea	279 (211 - 365)	247 (189 - 322)	7615 (5659 - 10148)	10.3 (8.1 - 13.2)	10.0 (7.9 - 12.7)	239.0 (183.7 - 310.2)
Ethiopia	3199 (2401 - 4461)	2855 (2128 - 4003)	79045 (58535 - 109671)	7.7 (5.8 - 10.7)	7.3 (5.5 - 10.4)	168.6 (124.8 – 236.0)
Kenya	1784 (1423 - 2206)	1628 (1282 - 2038)	45322 (35340 - 57167)	8.2 (6.7 – 10.0)	8.1 (6.5 – 10.0)	183.8 (144.5 - 230.4)
Madagascar	802 (582 - 1078)	714 (528 - 957)	21577 (15499 - 29044)	7.3 (5.4 - 9.7)	7.1 (5.3 - 9.3)	167.6 (123.7 - 224.6)

Location	Incidence - number of cases (95% UI)	Mortality - number of deaths (95% UI)	DALYs (95% UI)	Age-standardised incidence rate (95% UI)	Age-standardised Mortality rate (95% UI)	Age-standardised DALY rate (95% UI)
Malawi	442 (335 - 562)	402 (308 - 507)	10733 (7920 - 13894)	6.3 (4.9 - 7.8)	6.1 (4.7 - 7.5)	134.7 (102.1 - 170.7)
Mozambique	900 (649 - 1189)	832 (607 - 1092)	22365 (15783 - 29824)	8.7 (6.4 - 11.4)	8.7 (6.5 - 11.2)	189.2 (136.7 - 248.6)
Rwanda	549 (424 - 704)	480 (377 - 608)	13346 (9965 - 17536)	9.3 (7.4 - 11.7)	8.8 (7.1 - 10.8)	199.2 (154.8 - 253.2)
Somalia	330 (206 - 615)	310 (197 - 580)	9587 (6070 - 17896)	5.0 (3.1 - 9.2)	5.0 (3.2 - 9.3)	121.2 (76.6 – 226.0)
South Sudan	367 (236 - 552)	346 (223 - 528)	9502 (5890 - 14741)	9.9 (6.6 - 14.7)	10.0 (6.6 - 14.8)	223.4 (142.4 - 340.6)
Uganda	1737 (1348 - 2150)	1519 (1194 - 1857)	43114 (32533 - 54450)	12.3 (9.8 - 14.8)	11.6 (9.2 - 13.8)	267.3 (208.9 - 329.3)
United Republic of Tanzania	2457 (1926 - 3175)	2180 (1732 - 2794)	59014 (45123 - 78208)	10.1 (8.1 - 12.7)	9.5 (7.7 - 11.8)	217.1 (171.0 - 282.2)
Zambia	924 (671 - 1201)	791 (581 - 1018)	23332 (16632 - 30748)	13.6 (10.1 - 17.4)	12.6 (9.5 – 16.0)	296.8 (215.9 – 383.0)
**High-income Asia Pacific**	**196371 (166417 - 225643)**	**76929 (64821 - 83603)**	**1327823 (1186117 - 1414814)**	**44.6 (38.4 - 51.1)**	**15.3 (13.4 - 16.4)**	**323.9 (298.6 - 342.1)**

Location	Incidence - number of cases (95% UI)	Mortality - number of deaths (95% UI)	DALYs (95% UI)	Age-standardised incidence rate (95% UI)	Age-standardised Mortality rate (95% UI)	Age-standardised DALY rate (95% UI)
Brunei Darussalam	138 (122 - 159)	72 (64 - 82)	2013 (1771 - 2280)	49.4 (43.4 - 55.9)	30.3 (26.6 - 34.1)	626.1 (552.5 – 710.0)
Japan	160211 (130730 - 186831)	63742 (53256 - 69584)	1055345 (938927 - 1126078)	47.6 (40.2 - 55.6)	15.9 (13.9 - 16.9)	343.0 (316.0 - 361.5)
Republic of Korea	32929 (27484 - 39146)	12004 (10649 - 13220)	246590 (222651 - 270174)	37.2 (31.1 – 44.0)	13.9 (12.2 - 15.3)	280.8 (253.9 - 307.5)
Singapore	3093 (2471 - 3847)	1111 (991 - 1197)	23876 (21790 - 25814)	39.9 (31.9 - 49.5)	14.9 (13.2 - 16.1)	303.7 (276.8 - 328.4)
**High-income North America**	**260911 (229909 - 295693)**	**95664 (88321 - 99688)**	**1987109 (1895869 - 2059774)**	**42.7 (37.6 - 48.6)**	**14.9 (13.9 - 15.5)**	**339.9 (325.9 - 351.9)**
Canada	33633 (26448 - 42308)	11616 (10433 - 12532)	225956 (207794 - 242471)	49.6 (38.8 - 62.8)	16.2 (14.7 - 17.4)	346.0 (320.1 - 370.6)
Greenland	32 (26 - 38)	20 (16 - 24)	482 (390 - 583)	47.4 (39.6 - 55.7)	31.4 (26.0 - 37.1)	680.3 (555.7 - 812.4)
United States of America	227242 (197022 - 261375)	84026 (77987 - 87516)	1760640 (1681318 - 1826205)	41.9 (36.1 - 48.2)	14.8 (13.9 - 15.3)	338.9 (324.9 - 350.8)

Location	Incidence - number of cases (95% UI)	Mortality - number of deaths (95% UI)	DALYs (95% UI)	Age-standardised incidence rate (95% UI)	Age-standardised Mortality rate (95% UI)	Age-standardised DALY rate (95% UI)
**North Africa and Middle East**	**60010 (53354 - 67555)**	**39147 (34761 - 44107)**	**1013634 (896161 - 1146526)**	**13.9 (12.3 - 15.6)**	**9.8 (8.7 – 11.0)**	**218.7 (194.1 - 246.5)**
Afghanistan	1168 (749 - 1606)	1046 (686 - 1420)	33295 (20504 - 46503)	8.7 (6.0 - 11.5)	8.4 (6.0 - 11.2)	209.3 (137.2 - 281.8)
Algeria	3407 (2668 - 4282)	2384 (1889 - 2951)	57596 (45214 - 72440)	10.5 (8.3 – 13.0)	8.0 (6.4 - 9.8)	166.0 (130.8 - 206.5)
Bahrain	163 (123 - 211)	84 (64 - 106)	2476 (1882 - 3176)	17.0 (13.2 - 21.1)	11.4 (9.1 – 14.0)	223.7 (175.0 – 280.0)
Egypt	6520 (4676 - 9006)	4557 (3300 - 6275)	133038 (95340 - 183321)	9.8 (7.1 - 13.4)	7.4 (5.4 - 10.1)	184.4 (133.6 - 253.6)
Iran (Islamic Republic of)	10183 (9381 - 11029)	6413 (5903 - 6908)	159445 (148776 - 171672)	13.9 (12.8 - 15.1)	9.3 (8.5 - 10.1)	206.7 (192.2 - 222.1)
Iraq	2647 (2045 - 3379)	1797 (1401 - 2258)	51191 (39091 - 66602)	11.1 (8.7 - 13.8)	8.3 (6.6 - 10.2)	195.2 (151.5 - 247.4)
Jordan	1258 (1037 - 1520)	754 (628 - 901)	20411 (16995 - 24617)	19.1 (16.0 - 22.9)	13.1 (10.9 - 15.5)	282.7 (235.5 - 339.7)
Kuwait	454 (377 - 545)	223 (187 - 265)	5693 (4768 - 6824)	17.8 (14.6 - 21.3)	10.1 (8.4 – 12.0)	202.3 (168.8 - 242.2)

Location	Incidence - number of cases (95% UI)	Mortality - number of deaths (95% UI)	DALYs (95% UI)	Age-standardised incidence rate (95% UI)	Age-standardised Mortality rate (95% UI)	Age-standardised DALY rate (95% UI)
Lebanon	1555 (1259 - 1991)	910 (740 - 1199)	19495 (15743 - 24699)	29.8 (24.1 - 38.1)	17.6 (14.3 - 23.1)	372.5 (300.3 - 471.8)
Libya	901 (658 - 1180)	620 (452 - 801)	17140 (12334 - 22493)	17.0 (12.4 - 21.8)	12.5 (9.1 - 15.8)	298.1 (217.6 – 386.0)
Morocco	3213 (2395 - 4072)	2482 (1856 - 3110)	63650 (47435 - 81128)	10.3 (7.7 – 13.0)	8.5 (6.3 - 10.5)	193.6 (145.4 – 245.0)
Oman	251 (200 - 333)	133 (108 - 167)	3789 (2998 - 5029)	15.3 (12.6 - 18.6)	10.2 (8.5 – 12.0)	199.1 (162.3 - 243.4)
Palestine	616 (521 - 716)	419 (358 - 487)	11254 (9574 - 13095)	26.1 (22.1 - 30.3)	19.6 (16.7 - 22.7)	434.7 (368.8 - 503.9)
Qatar	191 (137 - 258)	83 (61 - 110)	2644 (1936 - 3535)	25.1 (19.3 - 31.9)	16.5 (12.9 - 20.5)	283.0 (218.2 - 354.5)
Saudi Arabia	3167 (2418 - 4019)	1629 (1263 - 2045)	53803 (40724 - 69784)	15.4 (12.2 - 18.8)	9.7 (7.8 - 11.6)	223.1 (175.7 – 276.0)
Sudan	1564 (1105 - 2336)	1256 (901 - 1851)	34670 (23958 - 50931)	8.2 (6.0 - 12.3)	7.1 (5.3 - 10.6)	164.4 (116.9 - 242.6)
Syrian Arab Republic	1028 (744 - 1375)	685 (499 - 908)	17974 (12946 - 24204)	8.5 (6.3 - 11.3)	6.3 (4.7 - 8.2)	140.0 (102.1 - 185.7)

Location	Incidence - number of cases (95% UI)	Mortality - number of deaths (95% UI)	DALYs (95% UI)	Age-standardised incidence rate (95% UI)	Age-standardised Mortality rate (95% UI)	Age-standardised DALY rate (95% UI)
Tunisia	1801<br>(1300 - 2441)	1154<br>(843 - 1546)	26506<br>(19101 - 36063)	14.5<br>(10.5 - 19.5)	9.7<br>(7.2 – 13.0)	208.1<br>(151.0 - 280.7)
Turkey	18104<br>(14441 - 22119)	11194<br>(9055 - 13549)	259024<br>(205968 - 318284)	20.6<br>(16.4 – 25.0)	13.1<br>(10.6 - 15.8)	289.2<br>(230.4 - 354.8)
United Arab Emirates	762<br>(521 - 1030)	449<br>(306 - 608)	15820<br>(10845 - 21393)	21.4<br>(14.7 - 29.8)	17.4<br>(11.9 - 24.3)	327.1<br>(223.9 - 452.3)
Yemen	996<br>(736 - 1350)	833<br>(626 - 1119)	23690<br>(17133 - 32022)	7.4<br>(5.6 – 10.0)	6.7<br>(5.0 - 9.1)	156.0<br>(116.2 - 211.9)
**Oceania**	**691<br>(555 - 855)**	**551<br>(443 - 682)**	**16315<br>(12915 - 20556)**	**10.0<br>(8.2 - 12.1)**	**8.8<br>(7.2 - 10.7)**	**203.6<br>(163.6 - 252.5)**
American Samoa	10<br>(8 - 12)	7<br>(6 - 9)	180<br>(148 - 219)	20.8<br>(17.6 - 24.7)	16.6<br>(14.1 - 19.6)	368.5<br>(306.7 - 443.4)
Cook Islands	3<br>(3 - 4)	2<br>(2 - 2)	40<br>(33 - 49)	12.4<br>(10.3 - 15.1)	7.8<br>(6.5 - 9.3)	168.3<br>(136.5 - 206.9)
Fiji	95<br>(75 - 120)	75<br>(59 - 93)	2006<br>(1563 - 2523)	13.5<br>(10.9 - 16.6)	11.7<br>(9.5 - 14.2)	258.1<br>(204.7 - 320.4)
Guam	40<br>(33 - 47)	27<br>(22 - 31)	645<br>(539 - 769)	21.0<br>(17.6 - 24.8)	14.2<br>(12.0 - 16.7)	341.6<br>(286.5 - 407.5)

Location	Incidence - number of cases (95% UI)	Mortality - number of deaths (95% UI)	DALYs (95% UI)	Age-standardised incidence rate (95% UI)	Age-standardised Mortality rate (95% UI)	Age-standardised DALY rate (95% UI)
Kiribati	8 (6 - 10)	7 (5 - 9)	209 (154 - 281)	11.4 (8.7 - 15.1)	10.8 (8.3 – 14.0)	263.4 (197.6 - 350.1)
Marshall Islands	5 (3 - 6)	4 (3 - 5)	117 (85 - 155)	13.7 (10.5 - 17.4)	12.3 (9.5 - 15.4)	293.4 (220.2 - 379.3)
Micronesia (Federated States of)	11 (7 - 14)	8 (6 - 11)	241 (158 - 329)	15.4 (10.9 – 20.0)	13.1 (9.5 - 16.9)	310.1 (213.8 – 411.0)
Nauru	1 (1 - 1)	1 (0 - 1)	22 (14 - 29)	21.4 (14.8 - 27.8)	16.5 (11.7 - 21.2)	398.5 (268.2 - 519.9)
Niue	0 (0 - 1)	0 (0 - 0)	7 (5 - 8)	20.4 (15.7 - 26.1)	13.6 (10.8 – 17.0)	307.5 (230.0 - 399.4)
Northern Mariana Islands	15 (12 - 17)	8 (7 - 9)	220 (181 - 261)	28.9 (24.5 - 33.1)	18.3 (15.9 - 20.9)	404.7 (339.9 – 472.0)
Palau	4 (3 - 5)	2 (2 - 3)	62 (48 - 80)	19.9 (15.7 - 24.7)	13.7 (10.9 - 16.7)	291.3 (228.3 – 363.0)
Papua New Guinea	382 (273 - 515)	318 (229 - 425)	9813 (6952 - 13274)	8.1 (5.9 - 10.5)	7.5 (5.5 - 9.6)	175.7 (126.6 - 233.2)
Samoa	18 (15 - 23)	14 (11 - 17)	355 (274 - 445)	12.8 (10.3 - 15.7)	10.1 (8.2 - 12.3)	232.9 (183.1 - 289.1)

Location	Incidence - number of cases (95% UI)	Mortality - number of deaths (95% UI)	DALYs (95% UI)	Age-standardised incidence rate (95% UI)	Age-standardised Mortality rate (95% UI)	Age-standardised DALY rate (95% UI)
Solomon Islands	41<br>(26 - 56)	31<br>(20 - 42)	1060<br>(646 - 1441)	12.4<br>(8.4 - 16.1)	10.4<br>(7.4 - 13.3)	273.0<br>(177.7 – 361.0)
Tokelau	0<br>(0 - 0)	0<br>(0 - 0)	3<br>(2 - 4)	14.1<br>(10.5 - 18.4)	10.9<br>(8.1 – 14.0)	245.6<br>(178.5 - 323.8)
Tonga	7<br>(5 - 8)	5<br>(4 - 7)	120<br>(93 - 154)	8.3<br>(6.4 - 10.5)	6.9<br>(5.4 - 8.7)	149.3<br>(115.1 - 190.4)
Tuvalu	1<br>(1 - 2)	1<br>(1 - 1)	27<br>(19 - 35)	13.0<br>(9.6 - 16.9)	10.9<br>(8.1 – 14.0)	254.3<br>(184.4 - 332.4)
Vanuatu	17<br>(13 - 23)	15<br>(11 - 19)	417<br>(301 - 557)	10.1<br>(7.6 - 13.1)	9.3<br>(7.1 - 11.9)	220.3<br>(161.6 - 289.7)
**South Asia**	**113711<br>(98190 - 129352)**	**94846<br>(81524 - 109075)**	**2419098<br>(2078019 - 2782570)**	**8.3<br>(7.2 - 9.4)**	**7.3<br>(6.2 - 8.3)**	**165.1<br>(141.7 - 189.9)**
Bangladesh	7167<br>(4965 - 10209)	6023<br>(4166 - 8611)	143863<br>(99756 - 205795)	5.6<br>(3.9 – 8.0)	4.9<br>(3.4 - 7.1)	107.4<br>(74.6 - 152.7)
Bhutan	43<br>(26 - 59)	36<br>(22 - 49)	838<br>(495 - 1146)	8.0<br>(4.9 - 10.9)	7.0<br>(4.4 - 9.4)	145.1<br>(87.2 - 196.7)
India	95112<br>(79687 - 110631)	79098<br>(67137 - 92724)	1998681<br>(1694556 -	8.6<br>(7.2 - 9.9)	7.5<br>(6.3 - 8.8)	169.1<br>(143.8 – 199.0)

Location	Incidence - number of cases (95% UI)	Mortality - number of deaths (95% UI)	DALYs (95% UI)	Age-standardised incidence rate (95% UI)	Age-standardised Mortality rate (95% UI)	Age-standardised DALY rate (95% UI)
			2358983)			
Nepal	1249 (891 - 1727)	1084 (781 - 1501)	26399 (18642 - 36362)	5.9 (4.2 - 8.1)	5.4 (3.9 - 7.4)	115.8 (82.1 - 158.5)
Pakistan	10140 (8100 - 12938)	8605 (6927 - 10776)	249316 (199384 - 312981)	9.1 (7.3 - 11.6)	8.3 (6.7 - 10.4)	198.3 (159.2 - 248.7)
**Southeast Asia**	**117010 (96631 - 136244)**	**82024 (67617 - 94606)**	**2142434 (1780490 - 2482287)**	**19.3 (16.0 - 22.4)**	**14.4 (11.9 - 16.6)**	**334.0 (276.6 - 386.4)**
Cambodia	1983 (1563 - 2416)	1561 (1240 - 1881)	42135 (32919 - 52153)	16.7 (13.3 – 20.0)	14.0 (11.2 - 16.5)	329.1 (260.7 - 401.7)
Indonesia	39110 (26895 - 49833)	30242 (20642 - 38807)	807988 (557416 - 1039823)	18.5 (12.6 - 23.4)	15.6 (10.5 - 19.9)	350.6 (240.6 – 450.0)
Lao People's Democratic Republic	656 (465 - 874)	545 (389 - 721)	15427 (10929 - 20823)	14.9 (10.6 - 19.5)	13.3 (9.6 - 17.2)	315.1 (224.2 - 419.9)
Malaysia	7627 (5970 - 9403)	4838 (3829 - 5962)	114326 (88561 - 142867)	29.6 (23.3 - 36.5)	20.3 (16.1 - 24.8)	422.0 (331.7 - 524.8)
Maldives	41 (34 - 49)	24 (19 - 28)	566 (465 - 676)	13.8 (11.3 - 16.4)	8.7 (7.1 - 10.4)	173.8 (142.0 - 207.3)

Location	Incidence - number of cases (95% UI)	Mortality - number of deaths (95% UI)	DALYs (95% UI)	Age-standardised incidence rate (95% UI)	Age-standardised Mortality rate (95% UI)	Age-standardised DALY rate (95% UI)
Mauritius	344 (278 - 423)	215 (176 - 261)	5091 (4106 - 6239)	19.8 (16.1 - 24.2)	12.8 (10.5 - 15.5)	290.7 (235.6 - 355.5)
Myanmar	6913 (5085 - 9011)	5582 (4135 - 7152)	148391 (107637 - 195590)	15.0 (11.1 - 19.3)	12.8 (9.6 - 16.2)	302.6 (221.4 - 393.4)
Philippines	15220 (12313 - 18708)	11193 (9309 - 13497)	320716 (265900 - 389975)	18.9 (15.4 - 23.1)	14.9 (12.5 - 17.8)	370.3 (308.3 - 447.4)
Seychelles	39 (34 - 44)	26 (23 - 29)	625 (548 - 714)	35.7 (31.4 - 40.6)	25.3 (22.2 - 28.7)	554.2 (485.7 - 629.5)
Sri Lanka	2546 (1896 - 3346)	1510 (1129 - 1967)	34803 (25537 - 45949)	10.2 (7.6 - 13.2)	6.4 (4.8 - 8.3)	137.1 (101.1 - 179.7)
Thailand	17397 (12834 - 22839)	10533 (7839 - 13572)	243610 (178190 - 319798)	17.2 (12.7 - 22.5)	10.5 (7.9 - 13.6)	241.9 (177.4 - 316.6)
Timor-Leste	112 (76 - 144)	93 (64 - 119)	2400 (1611 - 3135)	13.9 (9.7 - 17.8)	12.3 (8.5 - 15.8)	280.8 (190.6 - 365.2)
Viet Nam	24869 (19211 - 30956)	15557 (12175 - 18985)	403551 (306601 - 505645)	26.4 (20.6 - 32.4)	17.5 (13.9 - 21.1)	409.3 (316.3 - 505.6)
**Southern Latin America**	**26866 (21480 - 33612)**	**17930 (16774 - 18975)**	**366436 (347729 - 385441)**	**32.2 (25.7 - 40.4)**	**21.2 (19.9 - 22.4)**	**447.6 (424.7 - 470.5)**

Location	Incidence - number of cases (95% UI)	Mortality - number of deaths (95% UI)	DALYs (95% UI)	Age-standardised incidence rate (95% UI)	Age-standardised Mortality rate (95% UI)	Age-standardised DALY rate (95% UI)
Argentina	18697 (14880 - 23362)	12886 (12008 - 13759)	267946 (252346 - 283535)	34.7 (27.6 - 43.4)	23.6 (22.0 - 25.1)	506.6 (477.2 - 536.6)
Chile	6006 (4800 - 7551)	3524 (3224 - 3795)	70896 (65757 - 75714)	25.1 (20.1 - 31.6)	14.7 (13.5 - 15.8)	298.5 (277.5 - 318.7)
Uruguay	2162 (1734 - 2690)	1520 (1389 - 1630)	27575 (25661 - 29468)	39.5 (31.6 - 49.5)	26.1 (24.1 - 27.8)	533.4 (498.2 - 568.5)
**Southern Sub Saharan Africa**	**7106 (6389 - 7882)**	**5922 (5329 - 6580)**	**147780 (132439 - 165539)**	**13.1 (11.8 - 14.5)**	**11.5 (10.4 - 12.7)**	**250.4 (225.1 - 279.3)**
Botswana	247 (171 - 334)	189 (133 - 252)	5194 (3524 - 7135)	18.8 (13.5 - 24.5)	15.8 (11.6 - 20.5)	346.2 (240.6 - 464.5)
Eswatini	80 (53 - 111)	70 (47 - 96)	1924 (1264 - 2738)	14.4 (9.8 - 19.7)	13.5 (9.3 - 18.2)	304.7 (203.6 - 426.8)
Lesotho	146 (104 - 194)	132 (96 - 174)	3600 (2529 - 4837)	12.0 (8.8 - 15.5)	11.7 (8.7 - 15.1)	266.1 (190.7 - 354.1)
Namibia	133 (103 - 172)	113 (89 - 143)	2774 (2114 - 3679)	9.7 (7.7 - 12.3)	8.7 (7.0 - 10.8)	188.2 (145.6 - 244.8)
South Africa	5566 (5002 - 6289)	4601 (4159 - 5198)	111491 (100297 - 126629)	12.9 (11.6 - 14.5)	11.2 (10.1 - 12.6)	240.5 (216.9 - 273.5)

Location	Incidence - number of cases (95% UI)	Mortality - number of deaths (95% UI)	DALYs (95% UI)	Age-standardised incidence rate (95% UI)	Age-standardised Mortality rate (95% UI)	Age-standardised DALY rate (95% UI)
Zimbabwe	935 (709 - 1183)	818 (623 - 1031)	22797 (17078 - 29260)	13.8 (10.6 - 17.2)	12.9 (9.9 - 16.2)	295.1 (224.1 - 372.2)
**Tropical Latin America**	**42891 (40118 - 44928)**	**27704 (25668 - 29090)**	**660129 (625562 - 687740)**	**17.8 (16.6 - 18.6)**	**11.7 (10.8 - 12.3)**	**268.3 (253.7 - 279.8)**
Brazil	41935 (39320 - 43999)	27052 (25085 - 28403)	644732 (611426 - 672419)	17.8 (16.6 - 18.6)	11.7 (10.8 - 12.3)	268.3 (254.0 - 279.8)
Paraguay	955 (731 - 1218)	652 (507 - 824)	15396 (11739 - 19750)	17.4 (13.4 - 22.1)	12.2 (9.5 - 15.3)	268.6 (206.4 - 343.2)
**Western Europe**	**382442 (332800 - 432448)**	**172454 (155345 - 181815)**	**3008234 (2815060 - 3152895)**	**42.4 (37.1 - 48.3)**	**17.3 (15.8 - 18.1)**	**351.2 (332.0 - 366.8)**
Andorra	79 (60 - 101)	34 (26 - 42)	649 (497 - 829)	56.6 (42.8 - 71.9)	23.0 (17.9 - 28.9)	466.3 (355.3 - 596.4)
Austria	5847 (4783 - 7109)	2603 (2355 - 2811)	46854 (43158 - 50277)	33.1 (26.7 - 40.6)	13.5 (12.4 - 14.5)	275.4 (254.2 - 294.8)
Belgium	8994 (7126 - 11223)	4325 (3880 - 4687)	73674 (67681 - 79242)	39.3 (30.9 - 49.5)	17.1 (15.6 - 18.4)	337.5 (312.9 – 361.0)
Cyprus	739 (638 - 843)	265 (230 - 301)	5205 (4555 - 5928)	38.1 (33.1 - 43.4)	14.3 (12.4 - 16.3)	273.6 (239.2 - 311.5)

Location	Incidence - number of cases (95% UI)	Mortality - number of deaths (95% UI)	DALYs (95% UI)	Age-standardised incidence rate (95% UI)	Age-standardised Mortality rate (95% UI)	Age-standardised DALY rate (95% UI)
Denmark	5577 (4364 - 7017)	2647 (2396 - 2872)	47755 (43566 - 51734)	48.4 (37.8 - 61.1)	21.7 (19.8 - 23.5)	431.1 (395.7 - 464.9)
Finland	3799 (2973 - 4805)	1616 (1455 - 1756)	28876 (26509 - 31339)	31.5 (24.6 – 40.0)	12.3 (11.3 - 13.4)	253.6 (233.4 - 275.1)
France	52277 (40682 - 65962)	25497 (22330 - 27996)	422215 (381664 - 458064)	38.5 (30.1 - 49.3)	16.4 (14.7 - 17.8)	328.7 (300.7 - 354.4)
Germany	78951 (62925 - 101417)	37552 (34131 - 40326)	647921 (598987 - 694996)	41.4 (32.7 - 53.8)	18.0 (16.5 - 19.3)	360.7 (336.7 - 385.9)
Greece	7903 (6276 - 9940)	4000 (3600 - 4305)	66397 (60982 - 71241)	33.2 (26.1 - 42.1)	14.9 (13.7 - 15.9)	297.9 (277.0 - 318.6)
Iceland	169 (147 - 195)	70 (61 - 78)	1291 (1159 - 1436)	30.7 (26.8 - 35.4)	11.8 (10.5 - 13.2)	241.2 (216.5 - 267.5)
Ireland	3431 (2687 - 4295)	1377 (1242 - 1498)	26562 (24283 - 28836)	46.0 (35.9 - 57.7)	18.0 (16.3 - 19.5)	362.7 (332.2 - 392.6)
Israel	3893 (3050 - 4900)	2008 (1797 - 2172)	36384 (33302 - 39185)	33.6 (26.3 - 42.6)	16.6 (14.9 - 17.8)	323.0 (296.7 - 346.8)
Italy	60514 (50073 - 71460)	24877 (22016 - 26457)	436750 (402100 - 459967)	43.5 (36.0 - 51.6)	15.8 (14.4 - 16.7)	331.0 (309.6 - 347)

Location	Incidence - number of cases (95% UI)	Mortality - number of deaths (95% UI)	DALYs (95% UI)	Age-standardised incidence rate (95% UI)	Age-standardised Mortality rate (95% UI)	Age-standardised DALY rate (95% UI)
Luxembourg	372<br>(302 - 451)	170<br>(147 - 192)	3075<br>(2665 - 3506)	37.2<br>(30.3 - 45.1)	16.0<br>(13.9 - 18.1)	313.5<br>(273.1 - 358.2)
Malta	303<br>(256 - 361)	136<br>(120 - 154)	2547<br>(2254 - 2880)	32.9<br>(27.8 - 39.1)	14.1<br>(12.4 - 15.9)	289.7<br>(256.5 - 327.3)
Monaco	57<br>(46 - 68)	25<br>(21 - 30)	436<br>(350 - 523)	60.7<br>(48.5 - 73.6)	24.3<br>(19.6 - 28.8)	494.1<br>(392.6 - 604.4)
Netherlands	18774<br>(14703 - 23598)	7774<br>(7036 - 8444)	142372<br>(130387 - 153821)	55.4<br>(43.3 - 69.8)	21.8<br>(19.8 - 23.5)	435.3<br>(402.3 - 469.4)
Norway	4746<br>(4026 - 5535)	2035<br>(1838 - 2176)	36432<br>(33544 - 38790)	49.5<br>(41.9 - 57.7)	19.8<br>(18.0 - 21.1)	393.9<br>(364.5 - 418.9)
Portugal	10245<br>(8094 - 12946)	5189<br>(4700 - 5586)	92569<br>(85464 - 99118)	45.3<br>(35.2 - 57.7)	20.1<br>(18.5 - 21.5)	421.3<br>(391.8 - 449.5)
San Marino	32<br>(25 - 42)	15<br>(11 - 21)	256<br>(172 - 363)	49.5<br>(37.9 - 65.5)	21.2<br>(14.3 - 29.6)	413.5<br>(272.8 - 603.3)
Spain	47432<br>(37493 - 60137)	20011<br>(17768 - 21746)	350229<br>(320237 - 377643)	50.1<br>(39.3 - 63.8)	18.8<br>(17.1 - 20.2)	387.3<br>(355.2 - 416.4)
Sweden	7720<br>(6590 - 8930)	3623<br>(3273 - 3863)	63293<br>(59047 - 67075)	37.0<br>(31.4 - 42.8)	16.0<br>(14.7 – 17.0)	322.7<br>(303.5 - 341.3)

Location	Incidence - number of cases (95% UI)	Mortality - number of deaths (95% UI)	DALYs (95% UI)	Age-standardised incidence rate (95% UI)	Age-standardised Mortality rate (95% UI)	Age-standardised DALY rate (95% UI)
Switzerland	5824<br>(4529 - 7396)	2234<br>(1980 - 2431)	40285<br>(36400 - 43855)	33.6<br>(25.9 - 42.7)	11.9<br>(10.7 - 12.8)	244.8<br>(222.3 - 265.1)
United Kingdom	54429<br>(45870 - 63942)	24220<br>(22210 - 25338)	433585<br>(409710 - 451674)	43.6<br>(36.6 - 51.5)	18.1<br>(16.8 - 18.8)	365.2<br>(347.8 - 379.1)
**Western Sub Saharan Africa**	**15321<br>(12895 - 17824)**	**13773<br>(11698 - 16069)**	**353242<br>(295571 - 420704)**	**8.7<br>(7.4 – 10.0)**	**8.4<br>(7.3 - 9.7)**	**176.1<br>(149.0 - 206.2)**
Benin	360<br>(283 - 465)	331<br>(261 - 423)	8549<br>(6539 - 11330)	7.8<br>(6.3 - 9.8)	7.6<br>(6.2 - 9.5)	161.5<br>(126.5 - 209.1)
Burkina Faso	641<br>(501 - 816)	585<br>(461 - 742)	15422<br>(11862 - 19947)	7.4<br>(5.9 - 9.4)	7.3<br>(5.8 - 9.1)	156.0<br>(122.4 - 198.6)
CÃ´te d'Ivoire	946<br>(724 - 1199)	850<br>(663 - 1066)	23373<br>(17376 - 30280)	9.6<br>(7.6 - 11.9)	9.4<br>(7.6 - 11.5)	199.3<br>(155.1 - 250.8)
Cabo Verde	56<br>(45 - 66)	47<br>(38 - 56)	951<br>(779 - 1124)	13.4<br>(10.7 - 15.7)	11.4<br>(9.0 - 13.4)	218.2<br>(177.2 - 258.2)
Cameroon	1267<br>(933 - 1684)	1114<br>(838 - 1460)	30027<br>(21577 - 40868)	11.2<br>(8.6 - 14.6)	10.6<br>(8.3 - 13.8)	229.3<br>(170.0 - 303.2)
Chad	391<br>(300 - 513)	369<br>(285 - 480)	9761<br>(7359 - 12987)	7.3<br>(5.7 - 9.4)	7.4<br>(5.8 - 9.3)	159.6<br>(122.0 - 210.8)

Location	Incidence - number of cases (95% UI)	Mortality - number of deaths (95% UI)	DALYs (95% UI)	Age-standardised incidence rate (95% UI)	Age-standardised Mortality rate (95% UI)	Age-standardised DALY rate (95% UI)
Gambia	64<br>(46 - 85)	58<br>(43 - 78)	1463<br>(1032 - 1978)	6.8<br>(5.0 - 9.2)	6.6<br>(4.8 - 8.8)	142.7<br>(101.6 - 191.7)
Ghana	1495<br>(1165 - 1901)	1273<br>(1003 - 1615)	34863<br>(26475 - 45098)	9.5<br>(7.6 - 11.9)	8.8<br>(7.0 - 10.9)	194.2<br>(152.1 - 246.9)
Guinea	390<br>(296 - 510)	370<br>(282 - 480)	9411<br>(7035 - 12395)	7.3<br>(5.6 - 9.4)	7.2<br>(5.5 - 9.2)	159.6<br>(120.6 - 209.5)
Guinea-Bissau	65<br>(49 - 83)	59<br>(44 - 74)	1692<br>(1241 - 2181)	9.3<br>(7.1 - 11.7)	9.1<br>(6.9 - 11.3)	205.8<br>(153.2 - 262.3)
Liberia	132<br>(89 - 189)	120<br>(81 - 171)	3163<br>(2078 - 4534)	6.8<br>(4.7 - 9.7)	6.7<br>(4.6 - 9.5)	140.6<br>(95.2 - 199.9)
Mali	675<br>(529 - 853)	615<br>(487 - 773)	16327<br>(12540 - 21004)	8.1<br>(6.4 - 10.1)	7.8<br>(6.3 - 9.7)	175.0<br>(136.7 - 220.6)
Mauritania	173<br>(130 - 220)	156<br>(119 - 195)	3583<br>(2606 - 4671)	8.8<br>(6.8 - 11.1)	8.3<br>(6.5 - 10.3)	168.0<br>(124.0 - 213.6)
Niger	406<br>(295 - 556)	374<br>(276 - 510)	10059<br>(7150 - 14047)	5.6<br>(4.2 - 7.6)	5.6<br>(4.2 - 7.5)	119.8<br>(87.9 – 164.0)
Nigeria	7084<br>(5312 - 8958)	6382<br>(4884 - 8138)	157285<br>(116487 - 205168)	8.9<br>(6.9 – 11.0)	8.6<br>(6.7 - 10.8)	174.7<br>(132.5 – 224.0)

Location	Incidence - number of cases (95% UI)	Mortality - number of deaths (95% UI)	DALYs (95% UI)	Age-standardised incidence rate (95% UI)	Age-standardised Mortality rate (95% UI)	Age-standardised DALY rate (95% UI)
Sao Tome and Principe	16 (12 - 22)	14 (10 - 19)	351 (246 - 476)	16.5 (12.0 - 22.2)	15.2 (11.2 - 20.6)	315.4 (225.4 - 427.9)
Senegal	635 (499 - 800)	585 (467 - 731)	14439 (11000 - 18603)	8.9 (7.2 - 11.1)	8.7 (7.1 - 10.8)	183.4 (144.5 - 232.7)
Sierra Leone	244 (185 - 316)	225 (172 - 290)	5780 (4264 - 7593)	7.1 (5.5 - 9.1)	7.0 (5.4 - 8.9)	148.5 (112.4 - 192.3)
Togo	280 (205 - 368)	246 (181 - 320)	6738 (4823 - 9014)	8.2 (6.1 - 10.5)	7.9 (5.9 – 10.0)	169.4 (124.2 - 221.4)
**Global**	**2.17 million (2.0 – 2.34 million)**	**1.09 million (1.02 - 1.15 million)**	**24.3 million (22.6 – 25.7 million)**	**26.7 (24.6 – 28.9)**	**13.7 (12.6 – 14.5)**	**295.5 (275.2 – 313.0)**

Numbers in parenthesis represent 95% uncertainty intervals (UIs). DALYs=Disability-adjusted Life Years. The age-standardised incidence rate, age-standardised mortality rate, and age-standardised DALY rate are shown per 100 000 person-years. Source: Global Burden of Diseases, Injuries and Risk Factors Study 2019.

Supplementary Table 4 Country-wise Percentage Change in Colorectal Cancer Burden, 1990-2019

Location	Incidence - number of cases (95% UI)	Mortality - number of deaths (95% UI)	DALYs (95% UI)	Age-standardised incidence rate (95% UI)	Age-standardised Mortality rate (95% UI)	Age-standardised DALY rate (95% UI)
**Andean Latin America**	**448.8 (336.0 to 586.4)**	**269.8 (201.9 to 349.8)**	**230.2 (163.3 to 312.7)**	**100.0 (59.6 to 148.7)**	**30.9 (7.4 to 59.2)**	**27.0 (1.8 to 58.4)**
Bolivia	378.8 (261.8 to 526.7)	280.7 (192.3 to 389.3)	241.8 (153.7 to 357.1)	74.6 (33.9 to 123.9)	38.5 (6.2 to 76.0)	29.9 (-2.7 to 71.9)
Ecuador	521.3 (393.3 to 692.3)	337.5 (251.4 to 450.1)	312.5 (224.8 to 429.8)	119.4 (74.7 to 178.8)	53.8 (24.4 to 91.9)	53.1 (21.1 to 95.4)
Peru	439.2 (290.5 to 642.2)	238.8 (148.3 to 359.6)	194.2 (112.3 to 309.1)	100.4 (45.2 to 175.9)	20.3 (-11.7 to 63.3)	16.0 (-16.6 to 61.4)
**Australasia**	**96.8 (63.0 to 138.6)**	**48.6 (38.9 to 57.9)**	**29.4 (22.0 to 37.2)**	**-6.2 (-22.7 to 14.7)**	**-33.5 (-37.2 to -29.9)**	**-36.3 (-39.6 to -32.5)**
Australia	100.7 (58.7 to 153.9)	49.7 (38.9 to 59.9)	30.8 (21.8 to 39.8)	-5.2 (-25.3 to 20.5)	-33.7 (-37.9 to -29.6)	-36.0 (-40.2 to -31.5)
New Zealand	81.1 (49.4 to 115.3)	44.4 (33.0 to 55.0)	24.0 (15.6 to 32.5)	-9.9 (-25.8 to 7.0)	-31.4 (-36.1 to -26.7)	-36.6 (-40.7 to -32.4)
**Caribbean**	**194.5 (153.9 to 237.7)**	**143.3 (112.2 to 175.9)**	**126.5 (94.7 to 160.3)**	**46.9 (26.5 to 68.6)**	**17.8 (2.4 to 33.9)**	**16.6 (0.2 to 34.0)**
Antigua and Barbuda	188.5 (142.4 to 239.3)	126.5 (92.2 to 165.6)	141.1 (102.2 to 183.9)	59.9 (35.1 to 86.8)	34.7 (15.7 to 56.7)	25.4 (5.9 to 47.1)

Location	Incidence - number of cases (95% UI)	Mortality - number of deaths (95% UI)	DALYs (95% UI)	Age-standardised incidence rate (95% UI)	Age-standardised Mortality rate (95% UI)	Age-standardised DALY rate (95% UI)
Bahamas	200.9 (139.3 to 273.1)	165.8 (113.3 to 229.9)	152.5 (100.8 to 214.8)	21.5 (-2.8 to 50.3)	6.8 (-13.6 to 31.6)	4.4 (-16.7 to 29.8)
Barbados	179.9 (130.4 to 233.1)	125.8 (88.6 to 165.5)	126.1 (83.3 to 170.9)	67.2 (37.9 to 98.9)	37.2 (13.7 to 60.7)	31.7 (7.3 to 57.6)
Belize	422.7 (341.2 to 516.9)	331.4 (267.5 to 406.9)	396.0 (319.2 to 486.8)	74.6 (47.9 to 106.0)	48.5 (27.1 to 74.0)	59.7 (35.4 to 88.6)
Bermuda	122.7 (83.5 to 173.1)	58.1 (32.2 to 90.9)	33.3 (9.6 to 62.9)	4.9 (-13.3 to 28.3)	-31.0 (-42.2 to -16.8)	-33.1 (-45.0 to -18.0)
Cuba	173.7 (123.0 to 230.7)	121.5 (82.7 to 164.7)	97.1 (60.9 to 141.5)	48.1 (20.4 to 79.2)	15.0 (-5.1 to 38.1)	9.9 (-10.5 to 34.5)
Dominica	60.4 (31.5 to 99.1)	52.3 (25.4 to 87.5)	52.6 (23.2 to 89.7)	29.0 (5.3 to 60.0)	23.4 (1.8 to 51.4)	19.8 (-4.0 to 50.0)
Dominican Republic	416.7 (267.9 to 598.7)	330.7 (208.8 to 474.5)	299.6 (181.2 to 439.4)	104.4 (44.5 to 175.0)	64.2 (18.2 to 118.6)	69.3 (19.4 to 128.0)
Grenada	137.9 (108.4 to 170.4)	85.7 (63.8 to 110.5)	109.1 (82.1 to 139.3)	60.3 (40.7 to 81.5)	35.8 (20.1 to 53.6)	29.2 (12.3 to 47.7)
Guyana	111.8 (58.3 to 176.8)	87.4 (42.1 to 142.0)	88.8 (39.6 to 149.3)	29.1 (-1.7 to 66.3)	15.1 (-10.4 to 46.9)	17.1 (-12.2 to 53.4)
Haiti	137.7 (59.0 to 252.3)	122.6 (48.7 to 226.8)	114.9 (43.0 to 218.5)	11.9 (-24.5 to 62.5)	5.0 (-29.0 to 51.4)	0.6 (-32.9 to 48.2)

Location	Incidence - number of cases (95% UI)	Mortality - number of deaths (95% UI)	DALYs (95% UI)	Age-standardised incidence rate (95% UI)	Age-standardised Mortality rate (95% UI)	Age-standardised DALY rate (95% UI)
Jamaica	205.8 (143.7 to 282.0)	159.0 (110.1 to 219.9)	183.5 (124.0 to 256.8)	85.0 (47.0 to 130.8)	55.2 (25.0 to 92.1)	69.2 (33.8 to 113.3)
Puerto Rico	207.2 (138.5 to 289.5)	133.0 (86.4 to 192.4)	105.3 (58.4 to 163.6)	63.3 (25.7 to 109.0)	12.8 (-11.7 to 43.3)	16.8 (-10.7 to 51.1)
Saint Kitts and Nevis	109.4 (70.8 to 152.9)	55.7 (30.2 to 84.6)	74.8 (38.0 to 114.3)	22.3 .(1.7 to 45.5)	-3.1 (-17.9 to 13.1)	-6.8 (-24.9 to 13.0)
Saint Lucia	203.0 (152.9 to 263.1)	151.6 (111.1 to 200.2)	143.1 (101.9 to 194.1)	22.3 (2.0 to 46.6)	-0.3 .(-16.0 to 18.2)	-0.2 (-16.8 to 20.3)
Saint Vincent and the Grenadines	151.5 (116.6 to 196.0)	126.0 (96.6 to 162.6)	128.5 (95.4 to 168.6)	35.6 (17.1 to 59.1)	21.9 (6.1 to 40.7)	23.4 (5.9 to 45.3)
Suriname	247.5 (184.5 to 322.1)	204.5 (152.3 to 266.7)	196.0 (143.2 to 262.1)	51.0 (24.5 to 82.6)	31.9 (10.0 to 58.9)	31.7 (8.3 to 60.5)
Trinidad and Tobago	146.5 (87.7 to 221.8)	107.7 (59.8 to 168.1)	101.5 (51.0 to 166.5)	10.7 (-15.7 to 43.9)	-9.8 (-30.4 to 16.0)	-5.1 (-28.8 to 25.2)
United States Virgin Islands	264.7 (190.6 to 348.7)	213.6 (152.6 to 281.6)	169.6 (111.2 to 233.1)	62.8 (30.1 to 100.4)	34.3 (8.8 to 63.3)	33.2 (4.9 to 66.3)
**Central Asia**	**62.3 (49.0 to 78.9)**	**45.8 (33.7 to 60.4)**	**35.2 (23.2 to 49.9)**	**8.8 (-0.3 to 19.1)**	**2.3 (-6.2 to 11.5)**	**-12.3 (-19.6 to -3.2)**
Armenia	75.1 (46.3 to 106.2)	57.5 (33.0 to 84.3)	24.5 (4.1 to 47.2)	17.9 (-1.7 to 39.0)	2.4 (-13.1 to 19.8)	-11.6 (-25.9 to 4.7)

Location	Incidence - number of cases (95% UI)	Mortality - number of deaths (95% UI)	DALYs (95% UI)	Age-standardised incidence rate (95% UI)	Age-standardised Mortality rate (95% UI)	Age-standardised DALY rate (95% UI)
Azerbaijan	124.6 (84.1 to 178.4)	96.6 (59.3 to 142.7)	80.9 (48.2 to 122.1)	25.1 (0.6 to 54.2)	15.6 (-8.7 to 43.3)	-1.3 (-19.2 to 21.2)
Georgia	12.9 (-6.9 to 34.6)	16.2 (-3.5 to 37.6)	-1.9 (-19.5 to 17.8)	22.2 (1.6 to 45.7)	19.1 (-1.1 to 41.0)	11.2 (-8.7 to 33.8)
Kazakhstan	37.0 (18.6 to 56.8)	16.4 (1.0 to 32.7)	6.9 (-8.2 to 22.7)	2.5 (-11.0 to 16.8)	-10.9 (-22.3 to 0.9)	-19.9 (-30.6 to -8.4)
Kyrgyzstan	9.8 (-5.7 to 26.9)	0.9 (-12.9 to 16.2)	-8.6 (-21.2 to 5.6)	-24.6 (-35.1 to -12.8)	-27.3 (-37.1 to -16.6)	-39.6 (-48.1 to -30.5)
Mongolia	154.8 (89.9 to 243.7)	117.1 (62.9 to 189.5)	128.6 (68.5 to 212.5)	17.1 (-11.7 to 55.3)	6.3 (-18.0 to 39.8)	-1.0 (-26.1 to 33.1)
Tajikistan	69.7 (34.4 to 112.4)	64.4 (31.4 to 103.3)	53.3 (20.2 to 91.7)	8.6 (-12.5 to 33.9)	15.2 (-6.5 to 40.5)	-12.2 (-29.9 to 9.1)
Turkmenistan	88.9 (51.8 to 136.4)	74.0 (40.2 to 115.3)	50.3 (19.5 to 89.3)	2.3 (-17.2 to 26.0)	-3.9 (-22.0 to 17.3)	-18.9 (-34.6 to 1.5)
Uzbekistan	124.6 (89.3 to 164.6)	98.8 (68.6 to 132.3)	92.8 (61.4 to 127.7)	44.8 (24.3 to 66.2)	47.1 (27.5 to 67.2)	12.0 (-4.5 to 29.5)
**Central Europe**	**103.1 (80.1 to 128.7)**	**67.3 (49.0 to 85.8)**	**46.8 (29.5 to 64.7)**	**40.4 (24.4 to 58.4)**	**9.5 (-2.7 to 21.8)**	**5.4 (-7.3 to 18.4)**
Albania	186.7	126.7	83.7	43.9	6.0	3.2

**Location**	**Incidence - number of cases (95% UI)**	**Mortality - number of deaths (95% UI)**	**DALYs (95% UI)**	**Age-standardised incidence rate (95% UI)**	**Age-standardised Mortality rate (95% UI)**	**Age-standardised DALY rate (95% UI)**
	(112.4 to 279.0)	(69.8 to 195.1)	(35.1 to 143.0)	(7.2 to 89.9)	(-19.5 to 38.3)	(-23.6 to 36.9)
Bosnia and Herzegovina	184.8<br>(122.5 to 261.0)	142.6<br>(90.1 to 203.1)	97.4<br>(53.5 to 149.0)	93.8<br>(50.5 to 144.5)	55.0<br>(21.7 to 91.8)	46.3<br>(14.1 to 84.5)
Bulgaria	76.2<br>(40.1 to 119.6)	55.2<br>(24.9 to 90.8)	32.2<br>(4.7 to 64.9)	55.7<br>(23.4 to 95.2)	29.4<br>(4.2 to 59.5)	22.2<br>(-3.6 to 53.1)
Croatia	100.0<br>(57.1 to 151.1)	66.5<br>(32.2 to 106.8)	41.5<br>(10.4 to 78.8)	44.0<br>(13.1 to 81.9)	11.4<br>(-11.8 to 38.7)	7.5<br>(-16.7 to 36.2)
Czechia	31.5<br>(7.0 to 59.7)	0.7<br>(-17.1 to 21.4)	-10.2<br>(-26.9 to 8.9)	-13.6<br>(-30.3 to 5.2)	-36.1<br>(-47.5 to -22.8)	-39.7<br>(-51.2 to -26.4)
Hungary	60.3<br>(32.8 to 92.4)	27.7<br>(7.1 to 50.8)	20.2<br>(-0.8 to 43.9)	23.7<br>(1.7 to 48.7)	-7.0<br>(-22.5 to 10.2)	-4.8<br>(-21.9 to 14.6)
Montenegro	121.6<br>(76.9 to 180.1)	96.1<br>(58.3 to 147.2)	78.9<br>(42.9 to 125.5)	39.9<br>(13.0 to 76.8)	21.3<br>(-1.6 to 51.7)	16.6<br>(-6.5 to 47.1)
North Macedonia	215.3<br>(143.8 to 298.6)	153.2<br>(99.1 to 219.1)	128.4<br>(77.4 to 190.6)	83.5<br>(42.7 to 131.2)	47.1<br>(17.1 to 84.3)	37.5<br>(7.6 to 74.5)
Poland	130.7<br>(97.4 to 171.9)	93.5<br>(65.1 to 125.6)	68.2<br>(42.2 to 97.6)	44.3<br>(23.4 to 69.7)	15.3<br>(-1.7 to 34.7)	8.4<br>(-8.6 to 27.5)
Romania	168.1<br>(122.1 to 222.1)	113.5<br>(78.3 to 155.7)	78.4<br>(46.5 to 115.6)	109.2<br>(72.3 to 151.7)	57.0<br>(30.3 to 88.6)	46.2<br>(20.2 to 77.1)

Location	Incidence - number of cases (95% UI)	Mortality - number of deaths (95% UI)	DALYs (95% UI)	Age-standardised incidence rate (95% UI)	Age-standardised Mortality rate (95% UI)	Age-standardised DALY rate (95% UI)
Serbia	120.7 (72.1 to 187.2)	76.6 (39.8 to 129.4)	51.0 (18.0 to 96.1)	59.4 (24.2 to 108.9)	20.3 (-5.2 to 56.4)	13.5 (-11.7 to 48.1)
Slovakia	128.0 (80.6 to 188.3)	72.5 (37.9 to 116.3)	56.0 (23.2 to 97.0)	47.9 (17.2 to 87.1)	10.7 (-11.2 to 39.2)	2.4 (-19.3 to 29.8)
Slovenia	114.8 (48.3 to 198.0)	73.7 (23.8 to 140.7)	46.6 (0.9 to 106.7)	24.6 (-13.9 to 74.0)	-8.5 (-35.3 to 26.7)	-13.3 (-40.5 to 22.2)
**Central Latin America**	**402.1 (332.9 to 483.0)**	**292.0 (241.0 to 349.4)**	**264.2 (214.1 to 322.9)**	**77.0 (52.8 to 105.3)**	**33.0 (15.8 to 52.5)**	**37.8 (19.0 to 59.4)**
Colombia	342.1 (239.6 to 462.6)	232.7 (160.3 to 322.8)	192.7 (123.9 to 277.5)	45.3 (11.9 to 85.8)	2.1 (-20.1 to 29.6)	6.2 (-19.0 to 36.7)
Costa Rica	484.1 (351.4 to 649.2)	380.5 (276.7 to 505.4)	349.0 (246.4 to 482.0)	99.7 (54.4 to 156.3)	59.0 (24.3 to 99.9)	60.3 (23.5 to 107.4)
El Salvador	396.4 (272.8 to 546.4)	290.5 (196.0 to 402.4)	242.3 (157.4 to 349.5)	150.1 (86.4 to 227.7)	87.9 (41.8 to 142.9)	84.4 (37.7 to 143.4)
Guatemala	572.4 (418.2 to 761.1)	474.7 (349.9 to 635.1)	419.2 (299.7 to 572.6)	112.8 (65.1 to 170.1)	77.9 (41.0 to 122.9)	75.1 (35.4 to 125.0)
Honduras	412.6 (266.6 to 609.0)	351.5 (225.8 to 523.6)	293.4 (182.7 to 438.7)	84.3 (31.9 to 155.2)	62.7 (16.9 to 124.4)	46.5 (5.1 to 102.2)
Mexico	421.4 (347.6 to 498.1)	304.5 (251.4 to 364.8)	289.2 (237.1 to 349.7)	89.2 (63.1 to 116.8)	41.7 (23.6 to 62.3)	51.3 (31.4 to 74.8)

Location	Incidence - number of cases (95% UI)	Mortality - number of deaths (95% UI)	DALYs (95% UI)	Age-standardised incidence rate (95% UI)	Age-standardised Mortality rate (95% UI)	Age-standardised DALY rate (95% UI)
Nicaragua	507.0 (372.7 to 675.2)	371.5 (268.6 to 507.3)	317.4 (226.6 to 425.0)	120.5 (72.0 to 188.3)	73.6 (34.9 to 128.3)	58.3 (23.5 to 101.5)
Panama	337.5 (237.3 to 461.1)	257.2 (178.1 to 352.8)	225.9 (148.9 to 320.1)	57.3 (21.5 to 101.7)	24.4 (-2.7 to 57.4)	22.3 (-6.8 to 57.6)
Venezuela	405.8 (279.7 to 564.9)	299.1 (203.6 to 417.0)	269.6 (175.4 to 390.9)	70.3 (28.6 to 122.2)	30.7 (-0.1 to 68.6)	32.3 (-0.8 to 75.3)
**Central Sub Saharan Africa**	**145.4 (67.6 to 245.4)**	**133.8 (60.6 to 223.7)**	**131.7 (58.5 to 224.8)**	**3.5 (-27.8 to 40.3)**	**-1.4 (-30.5 to 32.4)**	**-2.6 (-32.8 to 33.1)**
Angola	260.0 (143.6 to 436.2)	234.5 (132.2 to 394.3)	224.6 (119.4 to 394.0)	27.7 (-10.9 to 81.5)	20.5 (-12.4 to 71.0)	14.4 (-20.5 to 69.2)
Central African Republic	61.5 (13.4 to 124.0)	58.8 (11.9 to 119.3)	64.5 (14.8 to 131.0)	-13.7 (-38.2 to 16.6)	-14.3 (-38.4 to 14.5)	-14.7 (-39.6 to 17.1)
Congo	144.6 (59.2 to 278.8)	130.1 (50.7 to 253.6)	127.1 (45.6 to 264.1)	1.4 (-32.3 to 50.8)	-3.1 (-34.3 to 41.8)	-9.0 (-40.1 to 40.4)
Democratic Republic of the Congo	117.4 (37.5 to 216.2)	110.6 (33.1 to 204.9)	108.7 (31.4 to 206.3)	-5.7 (-39.1 to 34.2)	-9.3 (-41.5 to 28.2)	-9.2 (-42.3 to 30.8)
Equatorial Guinea	484.6 (202.0 to 827.1)	405.3 (162.0 to 697.3)	380.2 (148.2 to 686.9)	144.6 (28.5 to 288.9)	118.3 (14.9 to 241.5)	93.0 (0.2 to 203.6)
Gabon	106.0 (31.3 to 203.9)	88.4 (21.4 to 175.5)	89.2 (17.4 to 183.2)	11.1 (-27.6 to 60.4)	3.4 (-31.8 to 48.5)	-1.1 (-37.3 to 46.3)

Location	Incidence - number of cases (95% UI)	Mortality - number of deaths (95% UI)	DALYs (95% UI)	Age-standardised incidence rate (95% UI)	Age-standardised Mortality rate (95% UI)	Age-standardised DALY rate (95% UI)
**Eastern Asia**	**467.2 (367.8 to 585.9)**	**230.8 (174.1 to 295.5)**	**181.4 (132.4 to 238.1)**	**142.3 (101.1 to 190.9)**	**37.1 (15.1 to 62.3)**	**30.8 (8.5 to 56.7)**
China	474.0 (369.2 to 600.9)	230.0 (172.0 to 298.3)	181.6 (129.7 to 242.3)	144.1 (100.0 to 195.5)	36.2 (13.4 to 62.7)	30.5 (7.2 to 58.0)
Democratic People's Republic of Korea	114.1 (40.8 to 207.4)	109.6 (39.8 to 197.3)	86.3 (19.4 to 174.4)	10.7 (-25.7 to 55.5)	2.6 (-30.4 to 42.7)	1.9 (-33.8 to 46.9)
Taiwan (Province of China)	492.6 (364.8 to 661.8)	343.3 (254.8 to 454.2)	249.3 (174.0 to 345.9)	149.2 (95.4 to 219.8)	69.0 (35.2 to 111.4)	56.5 (23.1 to 99.4)
**Eastern Europe**	**50.6 (37.4 to 66.6)**	**27.4 (15.6 to 39.7)**	**15.2 (4.7 to 27.1)**	**24.0 (13.0 to 37.2)**	**1.6 (-7.8 to 11.6)**	**-3.4 (-12.2 to 6.6)**
Belarus	59.3 (26.3 to 103.4)	21.7 (-1.5 to 53.6)	7.4 (-15.1 to 38.0)	30.3 (3.5 to 66.8)	-2.5 (-21.0 to 23.3)	-11.0 (-29.9 to 14.7)
Estonia	76.3 (39.7 to 120.9)	33.3 (7.3 to 66.2)	5.0 (-17.7 to 33.3)	36.6 (7.1 to 72.6)	-4.7 (-24.6 to 20.0)	-15.2 (-34.0 to 7.2)
Latvia	34.1 (11.9 to 62.0)	15.9 (-2.4 to 37.9)	-5.6 (-21.1 to 14.3)	20.6 (0.4 to 47.2)	-1.9 (-17.5 to 17.0)	-11.1 (-26 to 8.0)
Lithuania	50.8 (24.5 to 80.9)	37.3 (14.3 to 62.3)	12.0 (-7.7 to 34.9)	18.5 (-2.5 to 42.9)	1.7 (-16.0 to 21.2)	-7.2 (-24.2 to 13.2)
Republic of Moldova	51.9 (32.8 to 73.4)	28.8 (13.0 to 46.3)	15.4 (0.1 to 31.9)	18.5 (3.2 to 35.2)	-2.4 (-14.3 to 10.6)	-7.1 (-19.4 to 6.4)

Location	Incidence - number of cases (95% UI)	Mortality - number of deaths (95% UI)	DALYs (95% UI)	Age-standardised incidence rate (95% UI)	Age-standardised Mortality rate (95% UI)	Age-standardised DALY rate (95% UI)
Russian Federation	71.4 (51.0 to 95.3)	44.2 (27.5 to 62.5)	29.8 (14.3 to 47.2)	33.8 (17.7 to 52.4)	8.8 (-3.9 to 22.5)	3.0 (-9.3 to 17.1)
Ukraine	8.7 (-8.4 to 26.9)	-4.7 (-18.4 to 11.8)	-9.5 (-23.0 to 6.5)	4.1 (-12.3 to 22.1)	-10.6 (-23.7 to 4.9)	-11.7 (-24.8 to 4.2)
**Eastern Sub Saharan Africa**	**173.8 (118.8 to 241.7)**	**159.5 (109.7 to 222.0)**	**154.3 (101.5 to 225.9)**	**25.9 (2.3 to 53.8)**	**20.3 (-1.5 to 45.5)**	**15.9 (-6.6 to 45.2)**
Burundi	78.6 (15.4 to 185.6)	68.1 (8.7 to 166.6)	75.9 (11.2 to 186.2)	-7.7 (-39.9 to 43.7)	-10.5 (-41.2 to 37.5)	-14.0 (-44.7 to 38.5)
Comoros	156.0 (64.3 to 371.4)	141.0 (55.8 to 322.4)	137.0 (48.5 to 398.4)	15.9 (-24.2 to 98.7)	8.8 (-27.9 to 79.8)	8.1 (-31.5 to 108.6)
Djibouti	461.3 (262.4 to 733.4)	428.9 (246.4 to 681.5)	403.3 (218.1 to 664.1)	32.5 (-10.1 to 88.5)	24.3 (-14.1 to 75.1)	22.8 (-18.9 to 80.2)
Eritrea	318.2 (187.4 to 532.8)	297.5 (172.5 to 495.2)	280.3 (160.6 to 478.3)	59.0 (11.7 to 132.1)	52.2 (6.3 to 119.8)	44.6 (-0.8 to 115.7)
Ethiopia	113.2 (54.1 to 242.1)	103.1 (45.6 to 216.4)	81.5 (25.9 to 200.5)	5.1 (-22.8 to 55.3)	0.0 (-26.3 to 44.4)	-9.6 (-35.7 to 42.6)
Kenya	316.9 (227.8 to 457.3)	315.2 (229.3 to 447.9)	327.5 (232.5 to 464.2)	56.7 (25.6 to 108.6)	58.7 (27.8 to 107.6)	57.2 (24.3 to 107.3)
Madagascar	127.7 (59.9 to 215.9)	114.8 (53.6 to 194.5)	126.1 (59.4 to 216.1)	7.8 (-23.2 to 45.8)	4.2 (-24.9 to 40.7)	2.0 (-27.5 to 40.6)

Location	Incidence - number of cases (95% UI)	Mortality - number of deaths (95% UI)	DALYs (95% UI)	Age-standardised incidence rate (95% UI)	Age-standardised Mortality rate (95% UI)	Age-standardised DALY rate (95% UI)
Malawi	151.2 (83.1 to 235.5)	143.4 (78.7 to 221.3)	134.9 (65.3 to 222.1)	32.1 (-2.1 to 72.5)	27.3 (-4.6 to 64.6)	24.7 (-9.6 to 65.8)
Mozambique	234.4 (146.0 to 350.2)	213.2 (129.8 to 317.9)	221.6 (131.9 to 341.1)	78.3 (31.2 to 136.3)	68.4 (24.2 to 121.3)	70.5 (24.7 to 128.2)
Rwanda	115.2 (50.3 to 254.2)	98.5 (41.0 to 219.7)	89.7 (27.0 to 221.4)	6.6 (-23.1 to 66.6)	0.1 (-26.3 to 51.9)	-7.8 (-35.5 to 51.0)
Somalia	147.2 (58.6 to 275.4)	146.5 (62.2 to 271.0)	147.0 (59.0 to 278.8)	-5.7 (-37.1 to 40.9)	-5.8 (-36.3 to 38.3)	-6.6 (-38.5 to 40.4)
South Sudan	56.6 (12.6 to 133.2)	52.1 (8.3 to 126.4)	58.2 (8.8 to 145.2)	-1.6 (-27.3 to 42.0)	-2.8 (-28.4 to 39.3)	-4.6 (-32.5 to 42.1)
Uganda	309.7 (205.6 to 435.7)	275.7 (184.3 to 383.2)	300.4 (193.2 to 437.8)	82.3 (39.6 to 131.3)	70.7 (31.8 to 114.9)	73.5 (29.6 to 126.2)
United Republic of Tanzania	191.0 (127.1 to 265.1)	175.9 (118.5 to 242.2)	175.1 (109.8 to 256.6)	27.1 (1.8 to 55.7)	20.5 (-2.9 to 46.1)	19.8 (-6.3 to 50.6)
Zambia	216.1 (124.6 to 328.5)	187.0 (105.9 to 287.2)	195.3 (103.7 to 309.0)	31.7 (-3.6 to 74.2)	21.1 (-10.2 to 58.3)	21.2 (-13.7 to 64.2)
**High-income Asia Pacific**	**154.4 (120.1 to 189.2)**	**124.0 (98.7 to 138.2)**	**64.7 (52.3 to 72.6)**	**15.2 (0.4 to 31.1)**	**-14.8 (-20.6 to -10.9)**	**-18.7 (-22.4 to -15.6)**
Brunei Darussalam	262.8 (181.0 to 355.2)	187.2 (125.8 to 254.2)	177.6 (115.1 to 245.8)	21.2 (-3.2 to 50.6)	0.0 (-19.9 to 22.6)	-3.9 (-24.4 to 18.3)

Location	Incidence - number of cases (95% UI)	Mortality - number of deaths (95% UI)	DALYs (95% UI)	Age-standardised incidence rate (95% UI)	Age-standardised Mortality rate (95% UI)	Age-standardised DALY rate (95% UI)
Japan	125.1 (90.2 to 160.9)	108.3 (83.2 to 122.8)	50.0 (37.6 to 57.4)	12.8 (-4.2 to 31.5)	-15.2 (-21.1 to -11.5)	-17.8 (-21.6 to -14.9)
Republic of Korea	555.1 (445.1 to 681.2)	274.7 (233.7 to 317.1)	176.1 (147.7 to 206.0)	119.0 (83.6 to 160.3)	17.5 (5.4 to 30.3)	3.1 (-7.2 to 13.9)
Singapore	232.2 (163.7 to 313.0)	121.4 (103.0 to 139.0)	89.0 (74.7 to 105.0)	-7.0 (-25.6 to 15.0)	-40.0 (-44.8 to -35.3)	-44.1 (-48.2 to -39.5)
**High-income North America**	**55.4 (36.7 to 76.0)**	**33.0 (28.9 to 36.5)**	**31.7 (28.1 to 35.4)**	**-10.0 (-21.1 to 2.5)**	**-25.3 (-27.3 to -23.5)**	**-22.9 (-24.9 to -20.7)**
Canada	128.5 (78.1 to 191.0)	84.0 (71.2 to 96.5)	63.7 (53.3 to 75.2)	9.1 (-15.1 to 39.2)	-17.2 (-22.4 to -11.9)	-19.7 (-24.6 to -14.2)
Greenland	150.3 (101.7 to 209.7)	113.5 (70.4 to 163.0)	83.7 (44.8 to 132.1)	25.5 (2.5 to 54.5)	5.4 (-14.4 to 29.3)	-1.1 (-21.3 to 23.1)
United States of America	48.4 (28.9 to 70.8)	28.1 (24.3 to 31.6)	28.5 (24.9 to 32.1)	-12.1 (-23.9 to 1.5)	-26.3 (-28.2 to -24.4)	-23.2 (-25.4 to -20.9)
**North Africa and Middle East**	**289.0 (209.8 to 396.0)**	**199.3 (140.5 to 280.1)**	**177.6 (120.1 to 250.0)**	**54.5 (24.4 to 98.0)**	**19.9 (-2.7 to 53.9)**	**13.4 (-9.5 to 43.1)**
Afghanistan	107.4 (45.4 to 204.1)	94.1 (36.3 to 181.7)	118.6 (50.7 to 228.2)	9.9 (-23.5 to 56.5)	7.1 (-25.0 to 51.7)	3.3 (-28.9 to 52.8)
Algeria	283.0 (177.0 to 412.9)	220.4 (132.4 to 328.8)	185.1 (105.7 to 283.5)	34.7 (-1.6 to 78.4)	11.2 (-17.7 to 45.8)	5.9 (-23.2 to 40.9)

Location	Incidence - number of cases (95% UI)	Mortality - number of deaths (95% UI)	DALYs (95% UI)	Age-standardised incidence rate (95% UI)	Age-standardised Mortality rate (95% UI)	Age-standardised DALY rate (95% UI)
Bahrain	555.1 (370.5 to 849.5)	340.9 (216.0 to 525.4)	337.3 (213.6 to 518.3)	19.8 (-12.4 to 69.8)	-8.2 (-32.1 to 25.7)	-16.8 (-39.1 to 17.4)
Egypt	261.5 (156.4 to 407.0)	193.6 (110.1 to 311.0)	184.3 (101.8 to 296.5)	64.9 (17.7 to 131.0)	36.5 (-1.0 to 90.0)	33.6 (-4.4 to 86.1)
Iran	370.9 (270.7 to 499.0)	285.2 (206.4 to 392.6)	229.5 (162.7 to 315.7)	66.3 (31.5 to 112.5)	29.9 (3.8 to 66.7)	24.8 (-0.4 to 57.7)
Iraq	319.0 (173.2 to 531.4)	232.1 (120.7 to 394.5)	248.8 (126.1 to 424.8)	38.7 (-7.4 to 107.0)	15.5 (-21.4 to 70.9)	13.6 (-25.3 to 69.5)
Jordan	555.6 (373.3 to 781.7)	389.7 (262.3 to 552.4)	356.1 (238.1 to 511.0)	35.7 (-1.3 to 83.1)	5.3 (-21.6 to 41.4)	-2.5 (-27.9 to 29.7)
Kuwait	610.1 (482.6 to 764.1)	456.7 (370.0 to 564.5)	380.0 (300.8 to 479.6)	75.7 (46.3 to 114.6)	37.8 (16.4 to 65.1)	26.4 (6.4 to 52.8)
Lebanon	361.9 (226.1 to 579.6)	231.5 (135.4 to 392.3)	186.6 (99.1 to 318.6)	92.9 (35.6 to 179.7)	30.7 (-7.0 to 92.2)	27.1 (-11.5 to 82.9)
Libya	237.9 (86.9 to 464.4)	187.0 (60.4 to 375.5)	190.0 (58.5 to 386.0)	21.4 (-32.6 to 101.6)	4.4 (-41.6 to 72.3)	3.9 (-42.6 to 72.9)
Morocco	255.2 (156.0 to 373.6)	207.4 (122.0 to 309.2)	192.6 (109.7 to 288.8)	55.2 (12.6 to 107.7)	35.6 (-0.4 to 80.4)	30.9 (-5.5 to 73.6)
Oman	330.7 (166.2 to 572.6)	198.0 (85.7 to 365.2)	195.5 (82.6 to 358.2)	70.1 (9.5 to 166.2)	32.0 (-13.8 to 108.1)	16.7 (-26.3 to 82.0)

Location	Incidence - number of cases (95% UI)	Mortality - number of deaths (95% UI)	DALYs (95% UI)	Age-standardised incidence rate (95% UI)	Age-standardised Mortality rate (95% UI)	Age-standardised DALY rate (95% UI)
Palestine	314.4 (188.1 to 539.4)	241.7 (137.9 to 418.3)	246.7 (139.7 to 432.6)	53.2 (6.4 to 135.9)	32.5 (-7.1 to 101.5)	24.7 (-14.1 to 91.8)
Qatar	1140.6 (692.4 to 1771.6)	630.5 (380.0 to 970.3)	644.2 (377.3 to 1021.1)	60.8 (10.1 to 135.0)	17.3 (-18.4 to 75.1)	4.1 (-28.5 to 48.8)
Saudi Arabia	715.3 (405.3 to 1173.1)	343.9 (173.8 to 605.9)	418.1 (212.3 to 734.7)	131.1 (46.7 to 259.6)	42.8 (-10.5 to 127.1)	47.1 (-7.9 to 132.5)
Sudan	222.9 (125.0 to 353.2)	181.0 (98.0 to 294.7)	179.4 (88.2 to 299.7)	58.8 (14.7 to 118.8)	41.6 (3.9 to 91.5)	35.5 (-6.1 to 91.9)
Syrian Arab Republic	199.8 (91.6 to 355.3)	142.3 (57.2 to 267.0)	112.9 (34.9 to 224.2)	37.7 (-10.6 to 108.9)	13.8 (-25.0 to 69.5)	3.5 (-33.7 to 56.8)
Tunisia	309.1 (180.7 to 489.4)	219.5 (121.6 to 359.5)	195.2 (101.6 to 330.5)	58.1 (9.4 to 127.4)	20.4 (-15.3 to 71.6)	20.5 (-16.5 to 74.7)
Turkey	238.4 (142.1 to 364.5)	144.6 (77.4 to 230.5)	107.6 (46.5 to 181.7)	39.5 (1.0 to 91.5)	-1.0 (-27.7 to 34.8)	-9.3 (-35.0 to 23.0)
United Arab Emirates	946.5 (488.4 to 1621.7)	702.7 (359.1 to 1219.1)	772.9 (389.7 to 1351.2)	8.2 (-36.0 to 71.2)	-6.2 (-44.1 to 48.0)	-9.8 (-47.6 to 44.9)
Yemen	260.8 (121.9 to 492.5)	233.7 (108.0 to 441.3)	229.0 (98.6 to 453.0)	30.6 (-17.7 to 109.0)	21.5 (-22.9 to 92.9)	19.2 (-26.1 to 94.5)
**Oceania**	**181.0 (125.4 to 247.3)**	**167.4 (114.8 to 230.1)**	**163.0 (108.4 to 230.1)**	**19.7 (-2.0 to 45.5)**	**13.8 (-6.1 to 37.7)**	**11.7 (-10.2 to 37.9)**

Location	Incidence - number of cases (95% UI)	Mortality - number of deaths (95% UI)	DALYs (95% UI)	Age-standardised incidence rate (95% UI)	Age-standardised Mortality rate (95% UI)	Age-standardised DALY rate (95% UI)
American Samoa	134.5 (81.9 to 202.7)	122.6 (74.2 to 182.8)	98.7 (53.1 to 156.7)	11.8 (-12.7 to 42.2)	1.5 (-19.8 to 28.0)	2.2 (-20.9 to 31.2)
Cook Islands	121.2 (69.9 to 190.7)	92.1 (51.7 to 146.3)	63.1 (23.6 to 118.0)	13.4 (-13.0 to 48.6)	-7.4 (-26.8 to 18.9)	-9.9 (-32.0 to 19.5)
Fiji	160.7 (90.4 to 258.6)	149.0 (82.8 to 237.6)	124.2 (60.7 to 209.4)	31.9 (-2.7 to 76.0)	24.5 (-6.5 to 65.2)	19.8 (-12.1 to 62.9)
Guam	148.6 (96.0 to 212.4)	148.1 (97.4 to 211.4)	115.4 (71.4 to 168.8)	-3.4 (-23.6 to 22.0)	-17.2 (-34.0 to 5.9)	-5.4 (-24.6 to 19.1)
Kiribati	82.3 (28.2 to 171.0)	72.8 (21.7 to 155.9)	71.0 (19.5 to 155.5)	2.2 (-26.9 to 48.6)	-0.8 (-28.3 to 43.6)	-6.3 (-33.6 to 37.7)
Marshall Islands	146.8 (78.4 to 234.3)	124.7 (62.7 to 202.4)	137.0 (71.0 to 221.0)	19.5 (-11.0 to 58.7)	12.7 (-15.9 to 48.7)	12.7 (-17.6 to 51.7)
Micronesia	90.1 (23.6 to 167.0)	66.2 (9.3 to 131.5)	65.3 (3.5 to 134.3)	28.5 (-14.0 to 76.6)	15.8 (-20.1 to 56.1)	11.7 (-27.1 to 55.8)
Nauru	14.5 (-18.6 to 58.1)	-1.4 (-29.2 to 35.4)	4.3 (-26.2 to 43.6)	1.4 (-25.9 to 36.3)	-8.8 (-33.1 to 21.4)	-9.2 (-34.3 to 22.5)
Niue	27.4 (-2.6 to 65.2)	-0.2 (-22.6 to 27.6)	3.3 (-23.5 to 35.8)	31.2 (-0.4 to 72.4)	6.4 (-17.5 to 36.4)	4.4 (-23.5 to 39.3)
Northern Mariana Islands	192.5 (118.3 to 281.8)	167.5 (101.9 to 240.9)	115.1 (56.9 to 188.7)	8.3 (-16.2 to 33.1)	-6.8 (-26.7 to 14.0)	-8.4 (-30.5 to 14.6)

Location	Incidence - number of cases (95% UI)	Mortality - number of deaths (95% UI)	DALYs (95% UI)	Age-standardised incidence rate (95% UI)	Age-standardised Mortality rate (95% UI)	Age-standardised DALY rate (95% UI)
Palau	146.7 (71.5 to 244.7)	102.2 (42.1 to 179.2)	104.1 (40.1 to 184.2)	16.4 (-17.9 to 60.0)	-0.6 (-29.6 to 35.4)	-2.8 (-31.7 to 34.6)
Papua New Guinea	216.1 (135.9 to 327.9)	200.7 (125.4 to 306.4)	203.5 (124.7 to 313.9)	23.1 (-6.1 to 64.4)	18.2 (-8.9 to 55.5)	16.5 (-12.7 to 56.7)
Samoa	80.4 (34.8 to 141.4)	62.1 (23.6 to 111.7)	59.8 (16.1 to 119.0)	7.9 (-18.2 to 41.6)	-3.2 (-24.8 to 25.4)	-3.8 (-28.5 to 29.0)
Solomon Islands	199.3 (112.0 to 308.9)	173.5 (95.8 to 271.5)	174.4 (91.9 to 274.1)	32.6 (-2.7 to 79.3)	23.0 (-9.0 to 65.2)	19.4 (-14.3 to 62.4)
Tokelau	26.9 (-5.6 to 68.9)	4.6 (-20.4 to 36.2)	6.6 (-22.8 to 44.3)	29.7 (-3.5 to 71.9)	9.8 (-16.9 to 43.4)	6.1 (-23.2 to 44.7)
Tonga	83.6 (41.6 to 139.5)	77.5 (38.5 to 130.3)	57.0 (18.7 to 108.0)	24.6 (-3.0 to 62.3)	14.4 (-10.0 to 47.5)	12.3 (-14.2 to 46.6)
Tuvalu	78.6 (32.1 to 143.2)	62.2 (20.4 to 119.3)	51.1 (10.1 to 108.2)	19.9 (-11.3 to 61.1)	8.1 (-20.0 to 43.9)	4.1 (-23.7 to 42.6)
Vanuatu	232.6 (138.7 to 364.3)	220.2 (131.0 to 338.6)	211.3 (117.2 to 347.1)	26.7 (-7.4 to 71.4)	20.8 (-10.0 to 61.7)	22.1 (-13.0 to 70.8)
**South Asia**	**279.8 (201.2 to 353.4)**	**247.3 (177.9 to 314.5)**	**207.5 (146.3 to 267.0)**	**52.6 (20.6 to 81.6)**	**36.6 (8.5 to 62.8)**	**31.7 (5.0 to 57.6)**
Bangladesh	231.1 (125.5 to 380.0)	194.3 (101.7 to 323.2)	157.3 (72.8 to 289.3)	20.9 (-16.1 to 70.6)	6.8 (-25.4 to 48.4)	-1.0 (-32.3 to 44.3)

Location	Incidence - number of cases (95% UI)	Mortality - number of deaths (95% UI)	DALYs (95% UI)	Age-standardised incidence rate (95% UI)	Age-standardised Mortality rate (95% UI)	Age-standardised DALY rate (95% UI)
Bhutan	298.5 (187.7 to 456.3)	254.2 (157.7 to 386.4)	186.1 (102.6 to 316.8)	74.6 (27.9 to 135.5)	51.3 (12.3 to 103.6)	36.6 (-1.4 to 89.2)
India	291.1 (202.9 to 374.9)	260.0 (184.6 to 334.0)	210.5 (144.4 to 274.7)	53.6 (19.6 to 85.8)	37.1 (7.9 to 64.7)	32.1 (4.1 to 59.8)
Nepal	273.6 (167.2 to 412.1)	246.1 (150.8 to 375.1)	190.4 (102.5 to 306.4)	62.7 (18.1 to 122.2)	49.1 (9.8 to 104.0)	33.8 (-4.8 to 83.4)
Pakistan	226.0 (149.2 to 343.1)	190.0 (121.2 to 291.9)	220.9 (145.9 to 338.0)	66.4 (28.2 to 125.0)	53.5 (17.6 to 106.6)	55.3 (19.3 to 110.9)
**Southeast Asia**	**319.4 (249.4 to 385.2)**	**247.0 (189.6 to 300.1)**	**218.4 (166.6 to 265.8)**	**78.5 (47.8 to 106.9)**	**46.7 (21.8 to 70.0)**	**41.6 (18.3 to 62.8)**
Cambodia	362.4 (232.6 to 514.1)	300.7 (187.9 to 423.9)	264.4 (158.3 to 403.5)	78.8 (29.8 to 129.9)	55.2 (12.7 to 96.4)	45.9 (3.1 to 94.8)
Indonesia	311.3 (221.0 to 401.8)	265.8 (187.6 to 354.5)	230.9 (159.7 to 310.9)	94.5 (51.4 to 140.8)	74.1 (35.8 to 117.2)	61.4 (26.8 to 100.2)
Lao People's Democratic Republic	195.0 (100.8 to 313.9)	164.9 (80.9 to 264.0)	151.7 (70.0 to 265.7)	42.7 (-1.7 to 93.0)	30.4 (-9.4 to 75.0)	20.4 (-18.2 to 68.0)
Malaysia	364.3 (244.6 to 511.7)	253.7 (162.0 to 363.0)	229.2 (145.6 to 331.4)	61.1 (19.0 to 112.2)	24.9 (-8.0 to 64.2)	18.8 (-11.7 to 54.9)
Maldives	373.7 (233.8 to 634.2)	227.4 (131.9 to 395.1)	174.8 (92.5 to 340.8)	37.9 (-1.5 to 98.7)	-5.4 (-31.7 to 32.4)	-17.2 (-41.6 to 25.4)

Location	Incidence - number of cases (95% UI)	Mortality - number of deaths (95% UI)	DALYs (95% UI)	Age-standardised incidence rate (95% UI)	Age-standardised Mortality rate (95% UI)	Age-standardised DALY rate (95% UI)
Mauritius	351.5 (264.6 to 459.6)	282.7 (215.6 to 368.5)	243.9 (176.7 to 323.2)	90.0 (54.3 to 135.6)	55.5 (28.5 to 89.7)	53.5 (24.4 to 89.1)
Myanmar	216.3 (107.2 to 335.9)	180.6 (78.6 to 280.8)	151.4 (62.0 to 252.8)	62.8 (6.0 to 120.7)	43.8 (-7.7 to 94.3)	33.5 (-14.7 to 83.9)
Philippines	226.4 (155.7 to 324.6)	197.5 (137.9 to 284.8)	183.5 (126.7 to 264.7)	26.8 (0.3 to 63.1)	13.3 (-8.2 to 44.4)	15.4 (-7.4 to 48.6)
Seychelles	256.8 (205.8 to 314.3)	178.6 (140.4 to 221.9)	188.3 (145.8 to 235.3)	86.5 (60.1 to 115.0)	53.5 (32.5 to 77.2)	45.4 (24.9 to 68.3)
Sri Lanka	327.4 (205.3 to 469.8)	216.1 (129.3 to 316.5)	174.1 (95.2 to 266.6)	81.4 (31.0 to 139.4)	31.2 (-3.6 to 71.9)	25.9 (-9.3 to 68.1)
Thailand	285.3 (179.8 to 419.7)	194.9 (117.5 to 292.7)	144.8 (77.8 to 231.3)	36.5 (-1.0 to 83.2)	-2.2 (-28.0 to 30.2)	-4.0 (-30.6 to 29.7)
Timor-Leste	405.2 (230.0 to 629.9)	368.9 (209.3 to 578.3)	285.6 (144.3 to 469.5)	83.0 (22.4 to 164.1)	64.4 (10.5 to 136.8)	56.9 (2.2 to 127.8)
Viet Nam	524.7 (360.8 to 730.1)	346.6 (230.2 to 480.0)	356.1 (226.7 to 510.7)	165.4 (96.6 to 248.0)	94.9 (46.2 to 151.7)	92.9 (40.5 to 154.3)
**Southern Latin America**	**145.8 (96.6 to 204.8)**	**103.1 (92.7 to 114.2)**	**87.6 (77.9 to 97.7)**	**33.9 (6.8 to 66.2)**	**6.4 (1.2 to 12.0)**	**6.0 (0.8 to 11.8)**
Argentina	131.9 (84.8 to 189.1)	95.4 (83.1 to 108.6)	82.9 (71.8 to 94.9)	37.1 (8.8 to 70.7)	11.5 (5.0 to 18.9)	11.8 (4.9 to 18.9)

Location	Incidence - number of cases (95% UI)	Mortality - number of deaths (95% UI)	DALYs (95% UI)	Age-standardised incidence rate (95% UI)	Age-standardised Mortality rate (95% UI)	Age-standardised DALY rate (95% UI)
Chile	299.9 (217.1 to 404.1)	202.0 (179.8 to 226.1)	167.0 (147.0 to 188.5)	61.0 (28.0 to 102.6)	15.9 (8.1 to 24.8)	13.9 (5.6 to 22.7)
Uruguay	58.6 (27.4 to 98.7)	42.2 (32.8 to 52.2)	24.2 (16.4 to 32.9)	12.7 (-9.9 to 42.0)	-5.1 (-10.8 to 1.2)	-8.3 (-14.0 to -1.9)
**Southern Sub Saharan Africa**	**147.7 (121.4 to 184.0)**	**130.5 (105.2 to 168.9)**	**125.0 (98.8 to 158.7)**	**21.8 (8.1 to 41.1)**	**13.3 (-0.3 to 33.3)**	**12.5 (-0.3 to 30.2)**
Botswana	312.9 (177.6 to 477.3)	252.4 (142.1 to 388.1)	263.5 (136.0 to 420.2)	69.2 (18.0 to 128.7)	47.4 (3.8 to 98.7)	47.2 (-0.1 to 106.4)
Eswatini	177.9 (94.6 to 293.2)	161.5 (85.4 to 268.7)	169.2 (81.5 to 288.9)	38.7 (-0.8 to 92.2)	32.1 (-5.4 to 79.4)	35.0 (-5.5 to 91.1)
Lesotho	140.6 (54.4 to 250.0)	128.9 (49.2 to 231.9)	150.8 (57.3 to 269.4)	84.7 (21.9 to 165.6)	79.1 (20.0 to 153.7)	87.6 (19.7 to 174.3)
Namibia	226.0 (134.9 to 353.4)	193.2 (114.5 to 298.5)	180.3 (95.0 to 312.1)	67.8 (23.2 to 129.1)	51.1 (12.9 to 104.4)	43.7 (2.4 to 105.0)
South Africa	145.7 (117.7 to 182.1)	126.6 (99.6 to 163.5)	116.0 (91.9 to 146.8)	16.8 (2.1 to 36.1)	7.9 (-6.3 to 26.7)	5.1 (-6.5 to 20.8)
Zimbabwe	126.1 (65.4 to 195.4)	125.7 (66.1 to 195.0)	139.8 (70.8 to 216.5)	29.1 (-4.2 to 67.5)	29.2 (-2.9 to 68.6)	34.8 (-2.1 to 76.9)
**Tropical Latin America**	**300.2 (277.8 to 319.0)**	**226.9 (208.2 to 241.9)**	**197.2 (182.4 to 211.0)**	**48.0 (40.2 to 54.8)**	**15.6 (10.1 to 20.6)**	**18.3 (12.5 to 23.6)**

Location	Incidence - number of cases (95% UI)	Mortality - number of deaths (95% UI)	DALYs (95% UI)	Age-standardised incidence rate (95% UI)	Age-standardised Mortality rate (95% UI)	Age-standardised DALY rate (95% UI)
Brazil	297.3 (274.9 to 315.5)	224.4 (205.7 to 239.6)	194.6 (179.6 to 208.4)	46.6 (38.7 to 53.1)	14.4 (8.8 to 19.4)	17.2 (11.5 to 22.4)
Paraguay	495.1 (336.3 to 685.7)	384.3 (260.2 to 530.9)	373.7 (244.4 to 531.0)	138.0 (75.2 to 213.9)	91.9 (42.7 to 149.2)	94.7 (42.3 to 158.5)
**Western Europe**	**66.7 (47.6 to 87.9)**	**31.7 (25.0 to 36.7)**	**16.0 (11.2 to 20.1)**	**7.2 (-5.6 to 21.6)**	**-22.1 (-25.0 to -19.6)**	**-24.0 (-26.5 to -21.4)**
Andorra	177.6 (91.8 to 282.2)	137.9 (65.5 to 221.5)	101.0 (37.8 to 176.1)	6.3 (-26.5 to 44.2)	-20.1 (-43.3 to 5.4)	-19.9 (-44.8 to 9.3)
Austria	-0.6 (-18.6 to 21.4)	-20.5 (-26.4 to -14.8)	-29.7 (-34.4 to -25.0)	-34.1 (-46.7 to -19.1)	-50.2 (-53.3 to -46.8)	-53.2 (-56.2 to -50.0)
Belgium	37.0 (8.1 to 72.8)	8.2 (0.5 to 16.6)	-1.8 (-8.5 to 5.3)	-7.3 (-27.0 to 16.6)	-33.2 (-37.5 to -28.6)	-32.4 (-36.8 to -27.7)
Cyprus	361.7 (275.7 to 454.6)	176.5 (128.6 to 228.4)	151.6 (108.5 to 198.4)	86.5 (52.3 to 122.4)	7.8 (-11.4 to 28.6)	5.4 (-12.1 to 24.6)
Denmark	76.5 (38.0 to 120.7)	29.4 (19.7 to 39.2)	18.1 (9.1 to 27.7)	24.1 (-3.7 to 56.2)	-11.2 (-17.7 to -4.7)	-17.5 (-23.9 to -10.8)
Finland	103.4 (58.5 to 159.8)	53.9 (41.9 to 67.1)	34.9 (24.2 to 46.4)	20.3 (-6.8 to 54.5)	-15.7 (-21.8 to -9.0)	-17.3 (-23.8 to -9.9)
France	61.8 (28.5 to 104.3)	25.8 (15.1 to 36.1)	10.7 (2.3 to 19.4)	-0.6 (-21.9 to 26.0)	-30.3 (-35.2 to -25.2)	-30.7 (-35.8 to -25.4)

Location	Incidence - number of cases (95% UI)	Mortality - number of deaths (95% UI)	DALYs (95% UI)	Age-standardised incidence rate (95% UI)	Age-standardised Mortality rate (95% UI)	Age-standardised DALY rate (95% UI)
Germany	41.7 (11.6 to 81.6)	18.2 (9.8 to 26.4)	3.5 (-3.4 to 10.8)	-5.0 (-25.7 to 23.6)	-26.2 (-30.8 to -21.7)	-28.6 (-33.0 to -23.7)
Greece	105.6 (63.9 to 159.3)	87.6 (74.3 to 102.0)	56.6 (45.5 to 68.5)	31.7 (3.4 to 67.1)	4.8 (-2.0 to 11.8)	5.2 (-1.8 to 12.6)
Iceland	99.8 (70.7 to 132.3)	62.0 (43.9 to 82.3)	49.2 (32.3 to 68.3)	3.7 (-11.5 to 20.6)	-19.7 (-28.6 to -9.7)	-22.0 (-30.8 to -12.3)
Ireland	92.9 (50.8 to 142.7)	33.0 (22.5 to 44.3)	22.2 (12.2 to 32.7)	5.0 (-18.1 to 32.7)	-29.9 (-35.2 to -24.1)	-33.5 (-38.9 to -27.8)
Israel	153.2 (100.2 to 221.7)	93.8 (78.3 to 109.8)	73.8 (61.0 to 88.0)	5.6 (-16.8 to 34.8)	-24.8 (-30.1 to -18.9)	-26.1 (-31.4 to -20.1)
Italy	78.3 (48.3 to 109.7)	43.3 (32.0 to 50.1)	21.3 (14.4 to 26.4)	13.5 (-6.1 to 34.6)	-18.7 (-23.1 to -15.4)	-20.9 (-24.5 to -17.9)
Luxembourg	51.8 (23.1 to 83.8)	15.4 (1.0 to 29.9)	4.2 (-9.3 to 18.2)	-17.2 (-33.0 to 0.3)	-40.9 (-48.0 to -33.4)	-43.0 (-50.3 to -35.4)
Malta	153.8 (108.1 to 205.1)	93.6 (68.9 to 121.1)	70.8 (48.8 to 94.9)	17.1 (-3.6 to 40.0)	-17.2 (-27.3 to -5.8)	-17.1 (-27.7 to -5.8)
Monaco	78.8 (35.6 to 132.8)	48.5 (15.6 to 87.5)	41.5 (6.9 to 86.4)	33.0 (-1.5 to 76.5)	6.9 (-17.4 to 37.6)	4.4 (-22.5 to 40.4)
Netherlands	114.2 (68.6 to 168.2)	66.9 (55.0 to 79.3)	52.3 (41.9 to 64.2)	26.1 (-1.4 to 58.6)	-5.6 (-11.8 to 1.0)	-9.1 (-15.2 to -2.2)

Location	Incidence - number of cases (95% UI)	Mortality - number of deaths (95% UI)	DALYs (95% UI)	Age-standardised incidence rate (95% UI)	Age-standardised Mortality rate (95% UI)	Age-standardised DALY rate (95% UI)
Norway	70.0 (46.0 to 97.1)	26.3 (18.6 to 33.6)	17.9 (10.8 to 25.0)	21.1 (3.4 to 40.6)	-12.6 (-17.7 to -7.5)	-17.8 (-22.9 to -13.0)
Portugal	134.7 (85.2 to 195.6)	74.9 (61.0 to 87.8)	49.3 (38.3 to 59.6)	41.5 (10.5 to 79.8)	-9.3 (-15.6 to -3.4)	-8.3 (-14.9 to -1.8)
San Marino	116.9 (60.8 to 189.9)	96.1 (31.0 to 176.5)	71.5 (9.8 to 151.8)	11.7 (-18.4 to 50.6)	-10.1 (-41.2 to 28.4)	-9.2 (-42.6 to 35.2)
Spain	148.4 (96.8 to 213.9)	94.6 (79.4 to 109.2)	64.8 (52.6 to 77.4)	42.1 (10.9 to 81.3)	-0.6 (-6.9 to 6.2)	-3.9 (-10.9 to 3.1)
Sweden	52.9 (31.2 to 76.9)	26.9 (19.6 to 34.3)	17.2 (11.0 to 23.8)	10.4 (-5.2 to 27.6)	-12.3 (-16.7 to -7.5)	-15.3 (-19.7 to -10.7)
Switzerland	81.6 (41.7 to 129.8)	47.7 (35.2 to 58.7)	33.5 (22.5 to 43.8)	9.4 (-14.6 to 39.4)	-15.7 (-22.2 to -9.7)	-18.9 (-25.5 to -12.8)
United Kingdom	40.6 (18.7 to 65.5)	7.5 (3.1 to 11.4)	-2.3 (-5.5 to 0.7)	2.0 (-14.3 to 20.6)	-26.1 (-28.5 to -23.8)	-28.6 (-30.8 to -26.5)
**Western Sub Saharan Africa**	**181.9 (127.7 to 251.4)**	**165.4 (117.3 to 232.0)**	**165.7 (113.9 to 238.6)**	**33.2 (9.2 to 64.7)**	**27.3 (5.3 to 58.1)**	**22.4 (-0.1 to 53.9)**
Benin	189.1 (117.9 to 268.3)	171.9 (107.0 to 242.8)	182.9 (108.3 to 272.1)	22.0 (-5.6 to 52.2)	18.0 (-7.7 to 45.3)	13.2 (-14.4 to 45.6)
Burkina Faso	170.1 (117.3 to 241.5)	156.5 (107.8 to 221.9)	163.2 (104.5 to 239.3)	28.7 (5.5 to 59.9)	23.8 (2.0 to 51.5)	21.5 (-2.7 to 53.3)

Location	Incidence - number of cases (95% UI)	Mortality - number of deaths (95% UI)	DALYs (95% UI)	Age-standardised incidence rate (95% UI)	Age-standardised Mortality rate (95% UI)	Age-standardised DALY rate (95% UI)
CÃ´te d'Ivoire	181.2 (100.3 to 275.0)	176.5 (100.6 to 265.0)	159.2 (81.0 to 253.5)	8.3 (-18.5 to 39.0)	5.9 (-19.7 to 34.2)	1.0 (-26.7 to 33.2)
Cabo Verde	405.3 (302.4 to 509.1)	347.3 (250.7 to 439.7)	310.2 (233.1 to 396.7)	180.6 (121.1 to 237.3)	152.9 (97.3 to 204.0)	114.2 (71.8 to 159.4)
Cameroon	229.5 (128.3 to 356.9)	206.0 (116.0 to 321.4)	209.6 (111.0 to 343.8)	22.7 (-12.4 to 66.3)	15.7 (-16.6 to 55.9)	12.8 (-21.2 to 56.3)
Chad	172.5 (109.2 to 250.3)	160.7 (101.7 to 231.9)	178.7 (112.3 to 263.0)	40.4 (10.3 to 77.2)	38.2 (9.6 to 71.9)	34.5 (3.6 to 71.4)
Gambia	298.7 (156.0 to 497.7)	290.2 (154.2 to 475.9)	265.7 (130.3 to 463.2)	44.5 (-4.9 to 109.0)	39.0 (-6.2 to 100.1)	37.4 (-11.5 to 106.8)
Ghana	285.1 (178.6 to 426.6)	260.0 (161.7 to 389.0)	245.4 (145.4 to 382.8)	49.4 (9.4 to 101.1)	39.6 (3.7 to 86.6)	36.2 (-1.2 to 86.3)
Guinea	118.5 (56.8 to 205.8)	109.6 (49.2 to 191.1)	115.3 (52.2 to 202.2)	30.6 (-6.2 to 80.9)	26.0 (-9.0 to 73.6)	25.5 (-10.9 to 75.2)
Guinea-Bissau	88.7 (35.0 to 164.4)	80.2 (29.6 to 151.9)	84.2 (28.7 to 162)	8.2 (-21.0 to 48.7)	5.9 (-22.2 to 44.4)	0.5 (-28.5 to 40.2)
Liberia	84.6 (26.2 to 159.7)	71.3 (17.2 to 139.2)	83.7 (21.7 to 162.7)	2.4 (-29.7 to 41.9)	-2.0 (-31.6 to 35.1)	-6.1 (-35.5 to 32.3)
Mali	159.8 (99.2 to 243.2)	147.5 (91.9 to 223.6)	142.6 (82.6 to 226.7)	25.0 (-2.4 to 61.1)	19.9 (-6.1 to 53.8)	15.3 (-11.4 to 52.4)

Location	Incidence - number of cases (95% UI)	Mortality - number of deaths (95% UI)	DALYs (95% UI)	Age-standardised incidence rate (95% UI)	Age-standardised Mortality rate (95% UI)	Age-standardised DALY rate (95% UI)
Mauritania	108.4 (49.1 to 207.1)	94.7 (41.5 to 185.2)	78.0 (23.6 to 166.8)	3.5 (-24.4 to 50.4)	-2.6 (-27.8 to 39.1)	-12.3 (-38.0 to 30.8)
Niger	187.6 (116.6 to 279.2)	178.2 (112.6 to 263.2)	164.2 (93.6 to 255.9)	6.1 (-17.8 to 34.8)	3.4 (-20.0 to 30.7)	-2.2 (-25.6 to 28.1)
Nigeria	173.9 (102.7 to 287.1)	156.7 (85.4 to 263.7)	158.8 (82.6 to 278.3)	41.6 (6.5 to 95.3)	35.0 (-1.0 to 88.6)	29.8 (-6.8 to 85.2)
Sao Tome and Principe	172.3 (91.7 to 277.1)	143.5 (74.5 to 235.0)	154.9 (74.3 to 263.0)	69.6 (21.6 to 134.3)	55.1 (13.5 to 112.8)	52.4 (6.3 to 112.2)
Senegal	172.2 (105.0 to 256.1)	161.4 (99.7 to 238.0)	150.7 (83.0 to 235.0)	18.9 (-7.8 to 52.4)	14.4 (-9.5 to 46.0)	9.5 (-18.1 to 43.8)
Sierra Leone	116.4 (58.2 to 192.3)	102.2 (49.6 to 170.1)	115.4 (54.8 to 194.0)	17.5 (-12.7 to 57.0)	12.9 (-15.1 to 48.9)	10.5 (-19.6 to 49.3)
Togo	227.9 (144.9 to 333.1)	213.2 (134.8 to 311.5)	210.4 (127.8 to 317.7)	15.3 (-12.2 to 49.7)	11.9 (-14.1 to 44.4)	8.3 (-19.3 to 42.6)
**Global**	**157.2 (139.5 - 177.2)**	**109.6 (96.2 - 121.7)**	**95.7 (82.1 - 108.9)**	**20.1 (12.1 - 29.2)**	**-4.4 (-10.0 to 0.9)**	**-4.2 (-10.7 to 1.9)**

Numbers in parenthesis represent 95% uncertainty intervals (UIs). DALYs=Disability-adjusted Life Years. The age-standardised incidence rate, age-standardised mortality rate, and age-standardised DALY rate are shown per 100 000 person-years. Source: Global Burden of Diseases, Injuries and Risk Factors Study 2019.
